# Connecting the ruminant microbiome to climate change: insights from current ecological and evolutionary concepts

**DOI:** 10.3389/fmicb.2024.1503315

**Published:** 2024-12-02

**Authors:** A. Nathan Frazier, Matthew R. Beck, Heidi Waldrip, Jacek A. Koziel

**Affiliations:** ^1^Conservation and Production Research Laboratory, United States Department of Agriculture, Agricultural Research Service, Bushland, TX, United States; ^2^Department of Animal Science, Texas A&M University, College Station, TX, United States

**Keywords:** greenhouse gases, enteric methane, methanogenesis, cattle, inhibition, rumen microbiome, evolution, ecology

## Abstract

Ruminant livestock provide meat, milk, wool, and other products required for human subsistence. Within the digestive tract of ruminant animals, the rumen houses a complex and diverse microbial ecosystem. These microbes generate many of the nutrients that are needed by the host animal for maintenance and production. However, enteric methane (CH_4_) is also produced during the final stage of anaerobic digestion. Growing public concern for global climate change has driven the agriculture sector to enhance its investigation into CH_4_ mitigation. Many CH_4_ mitigation methods have been explored, with varying outcomes. With the advent of new sequencing technologies, the host–microbe interactions that mediate fermentation processes have been examined to enhance ruminant enteric CH_4_ mitigation strategies. In this review, we describe current knowledge of the factors driving ruminant microbial assembly, how this relates to functionality, and how CH_4_ mitigation approaches influence ecological and evolutionary gradients. Through the current literature, we elucidated that many ecological and evolutionary properties are working in tandem in the assembly of ruminant microbes and in the functionality of these microbes in methanogenesis. Additionally, we provide a conceptual framework for future research wherein ecological and evolutionary dynamics account for CH_4_ mitigation in ruminant microbial composition. Thus, preparation of future research should incorporate this framework to address the roles ecology and evolution have in anthropogenic climate change.

## 1 Introduction

Ruminants have evolved a unique digestive tract, composed of four distinct compartments: the reticulum, rumen, omasum, and abomasum. The first three compartments are considered the forestomach, whereas the abomasum represents the “true stomach” (analogous to monogastric stomachs) where acid and chemical digestion occurs. The reticulum and rumen are only separated by the reticulo-rumen fold and are often referred together as the reticulo-rumen. However, they do differ in terms of epithelial cell structure, where the reticulum epithelium is composed of 4–6 sided structures that appear to be “honey-combed” in shape, whereas the rumen epithelium is composed of papillae (Church, 1979). The rumen is a specialized fermenting foregut of the animal that houses a diverse symbiotic microbial community, responsible for the fermentation of ingested feedstuffs and production of volatile fatty acids (VFA) used by the host to meet its energy requirements. For example, while VFA provide 5–10% of energy requirements for monogastric species, they provide as much as 70% of energy requirements in ruminants (Bergman, 1990; Russell, 1998). Furthermore, microbial crude protein provides around 50–80% of the metabolizable protein requirements of ruminants (Storm and Ørskov, 1983). The microbial ecosystem within the rumen works to digest plant materials due to a plethora of encoded functional genes responsible for nutrient exploitation and digestion (Luckey, 2012; Hobson and Stewart, 2012; Mizrahi et al., 2021). Because of this unique symbiotic partnership, the ruminant host-microbe system is a well-studied model for the ecological and evolutionary relationships between hosts and their symbiotic microbial partners.

**Figure 1:**
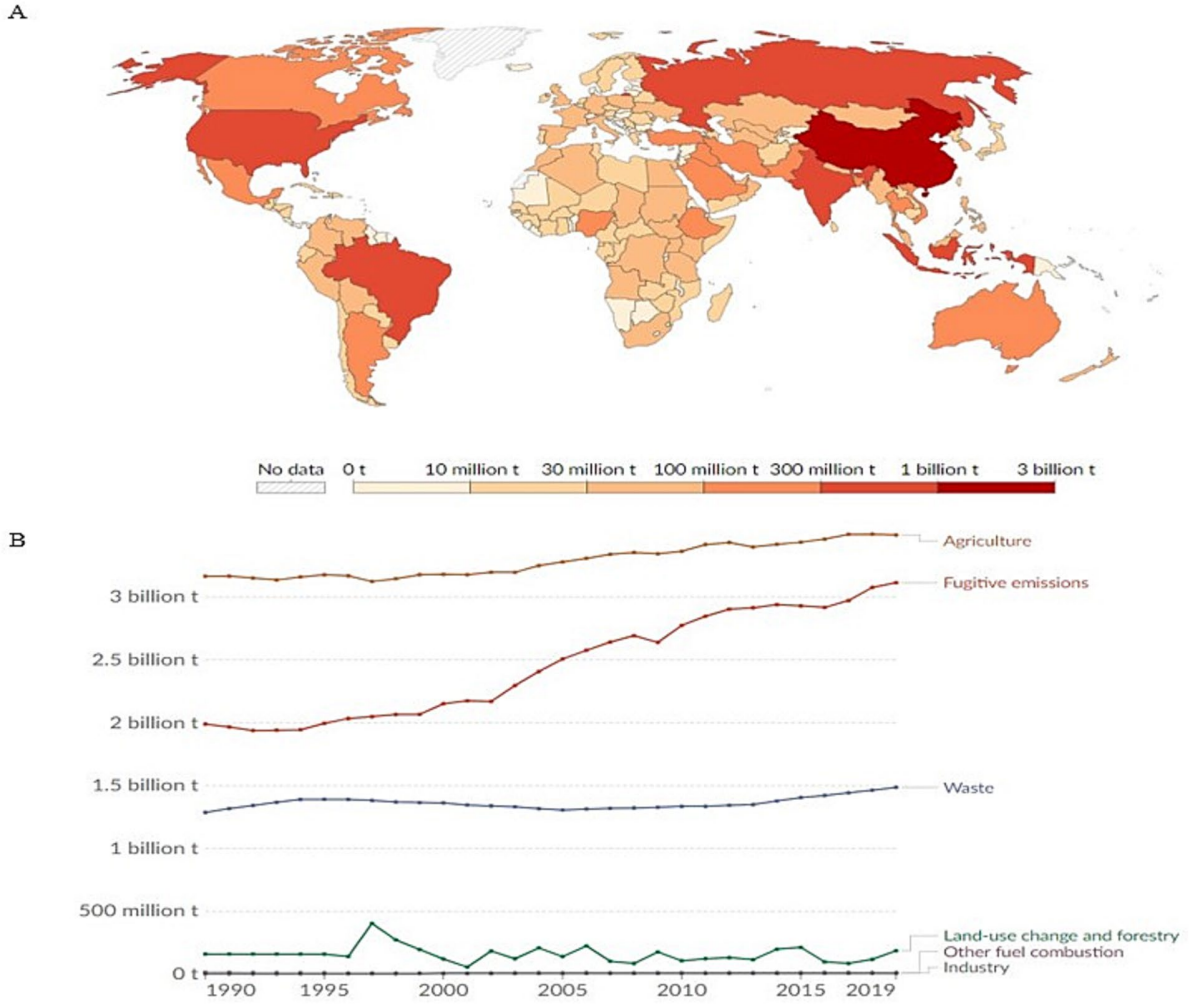
Global methane emissions. In panel A, the global CH_4_ emissions from 2021 are displayed measured in tons of CO_2_-equivalents. Data was sourced from Jones et al. (2024) and the graph was generated from ourworldindata.org/co2-and-greenhouse-gas-emissions (accessed 8/18/2023). Panel B displays the methane emissions by sector as measured in tons of CO_2_-equivalents and was generated from the Climate Analysis Indicators Tool from ourworldindata.org/co2-and-greenhouse-gas-emissions (accessed 8/18/2023).

The ruminant fermentation process responsible for nutrient production is a complex series of biochemical processes resulting in the release of carbon dioxide (CO_2_) and dihydrogen (H_2_) into the rumen headspace at the final step of anaerobic digestion (AD). Ungerfeld (2020) provides a detailed account of the biochemical principles in ruminant fermentation. Accumulation of H_2_ within the rumen has been reported to decrease animal health and productivity (Janssen, 2010; Leng, 2014). Therefore, a process called methanogenesis is necessary, where methanogens utilize various metabolite inputs to produce methane (CH_4_; see Supplementary material). Methaneis an important greenhouse gas (GHG) and is released into the atmosphere from the ruminant through eructation, respiration, flatulence, manure, and by ruminant feed production. Methane emissions from agriculture, forestry, and land use change accounts for 23% of GHG emissions with enteric CH_4_ accounting for 5% of the anthropogenic GHG emissions in the agricultural sector (Figure 1; Gerber et al., 2013; United Nations Environment Program and Climate and Clean Air Coalition, 2021; Dhakal et al., 2022). Furthermore, CH_4_ has a global warming potential that is 28–34 times higher than CO_2_ per unit mass and on a 100-y time scale, it is 82 times higher than that of CO_2_ (Edenhofer, 2015; Carnachan et al., 2019). The world’s human population saw a remarkable three-fold increase from the 20th century to 2022 (United Nations, 2023) and such a trend must be met with an increase in food supply to address global food security and poverty (OECD/FAO, 2020; Vollset et al., 2020). Therefore, CH_4_ emissions are expected to continue rising given a possible increase in livestock animal production.

Indeed, nature has methods of removing CH_4_ through natural occurring sinks. Natural sinks are defined as a place where substrates are stored by nature, such as plants, the atmosphere and soil. The largest natural sink for CH_4_ is the atmosphere itself where tropospheric hydroxyl radicals (OH) react with CH_4_ breaking down CH_4_ into CO_2_ and water vapor (Maasakkers et al., 2019). Additionally, soil is another primary natural sink for CH_4_ consumption entirely through methanotrophy. Methanotrophic microorganisms in the soil utilize CH_4_ as their sole source of carbon and energy (Flint et al., 2012; Guerrero-Cruz et al., 2021; Ahmadi and Lackner, 2024). However, recent research has indicated that the atmosphere and the soil are becoming less efficient at balancing CH_4_ removal for leveling climate change (Maasakkers et al., 2019; Mizrahi et al., 2021). Indeed, CH_4_ has a relatively short atmospheric lifespan of only 11.8 y (IPCC, 2021). Thus, change in CH_4_ emissions and emission rates can have an impact on climate change. As CH_4_ emissions decrease, atmospheric CH_4_ concentrations can be reduced, at least in the short term. Accordingly, if annual CH_4_ emission rates are reduced greater than −0.32% per year there would be a net-climate cooling effect (Beck et al., 2022a). Subsequently, the natural CH_4_ sinks could become more effective at removing CH_4_ at rates sufficient for climate change improvement. This nuance of CH_4_ as a climate forcer makes its mitigation the most promising mean to limit climate change in the short-term (Ocko et al., 2021; Beck et al., 2022; Beck et al., 2023). As such, there has been a significant amount of research into mitigation strategies and techniques that could prove viable in reducing CH_4_ in the environment (Mizrahi et al., 2021; Beauchemin et al., 2022).

As previously mentioned, an increased demand in food supply could be indicative of an increase in enteric CH_4_ emissions. Accordingly, mitigation strategies have been a focal point for livestock researchers in recent years. These mitigation strategies include inhibitory compounds such as 3-nitrooxypropanol (3-NOP; Pitta et al., 2022) and halogenated bromoform from seaweed (Roque B R, et al., 2019), dietary composition (Han et al., 2019), animal breeding and genetics (de Haas et al., 2017), secondary plant compounds (Kozlowska et al., 2020), and early-life microbiome engineering (Meale et al., 2021). Artificial intelligence approaches could also be harnessed to search for new inhibitory compounds (Chowdhury et al., 2024; Aryee et al., 2024). Methanotrophs offer a natural microbial ecosystem that has been explored for CH_4_ removal in ruminants (Flint et al., 2012). However, there is a lack of evidence *in vivo* to adequately utilize this technique within ruminant livestock (Tseten et al., 2022). Nevertheless, the rumen microbiome holds intriguing promise in addressing anthropogenic climate change due to the obligatory nature the host animal has with its symbiotic microbes. The ruminant microbiome is comprised of intricate microbial networks that provide the animal with nutrients for the host requirements. It is not surprising then that there is an inherent need for researchers to understand how the native microbiome is affected by microbiome engineering techniques resulting in unique changes that reduce enteric CH_4_.

In context of overall microbial ecology, the rapid improvement of sequencing technology has produced a vast abundance of empirical data while microbiome-related hypotheses and theory have staggered (Koskella et al., 2017). It is important to understand how pressures such as CH_4_ mitigation impact microbial ecological and evolutionary patterns in the rumen. The importance of theory cannot be understated as theory allows researchers to classify and interpret the many phenomena occurring in the biological world. Theory is key in microbial ecology and has an essential role in developing an understanding and explaining interactions between microbes and their environments (Prosser et al., 2007). A better grasp of these concepts could provide researchers more insight into how mitigating strategies will work (or fail) in the short- and long-term. Indeed, empirical research is crucial and necessary to test the many theories and hypotheses to interpret the classifications and interpretations made by theory for the complex phenomena within microbial ecology. However, theory is necessary to formulate testable hypotheses. Therefore, the primary goal of this review is to synthesize our current knowledge of the ruminant microbiome’s key drivers of microbial ecology in the ruminant, the relationship composition has to functionality (i.e., methanogenesis) and the effects CH_4_ mitigation strategies have on the native ruminant microbiome. Moreover, we discuss how current microbial evolutionary and ecological concepts apply to the ruminant microbiome and how these concepts can be leveraged for CH_4_ mitigation. We then discuss a potential conceptual framework for future theoretical and empirical studies into ruminant microbial ecology to address enteric-produced GHG emissions.

## 2 Establishment of the ruminant microbiome: stochastic or deterministic?

The microbiome within the rumen is a highly complex community represented by bacteria, archaea, protozoa, and fungi. Recent works have reviewed the ruminant microbial community composition in detail (Tapio et al., 2017; Huws et al., 2018; Mizrahi et al., 2021) and a more detailed description is provided within the Supplementary material. Ruminant microbial composition, diversity, and assemblage are governed by both stochastic and deterministic forces. While deterministic forces including diet, age, and host genetics were traditionally considered the primary forces of community assembly, recent evidence suggests that stochastic forces are important factors in microbiome compositionality and assembly (Evans et al., 2017; Furman et al., 2020). Stochastic processes include historical contingency, drift, and dispersal. Together, both stochastic and deterministic processes have long-lasting effects on ruminant microbial ecology.

### 2.1 Stochastic properties of microbial composition

**Figure 2:**
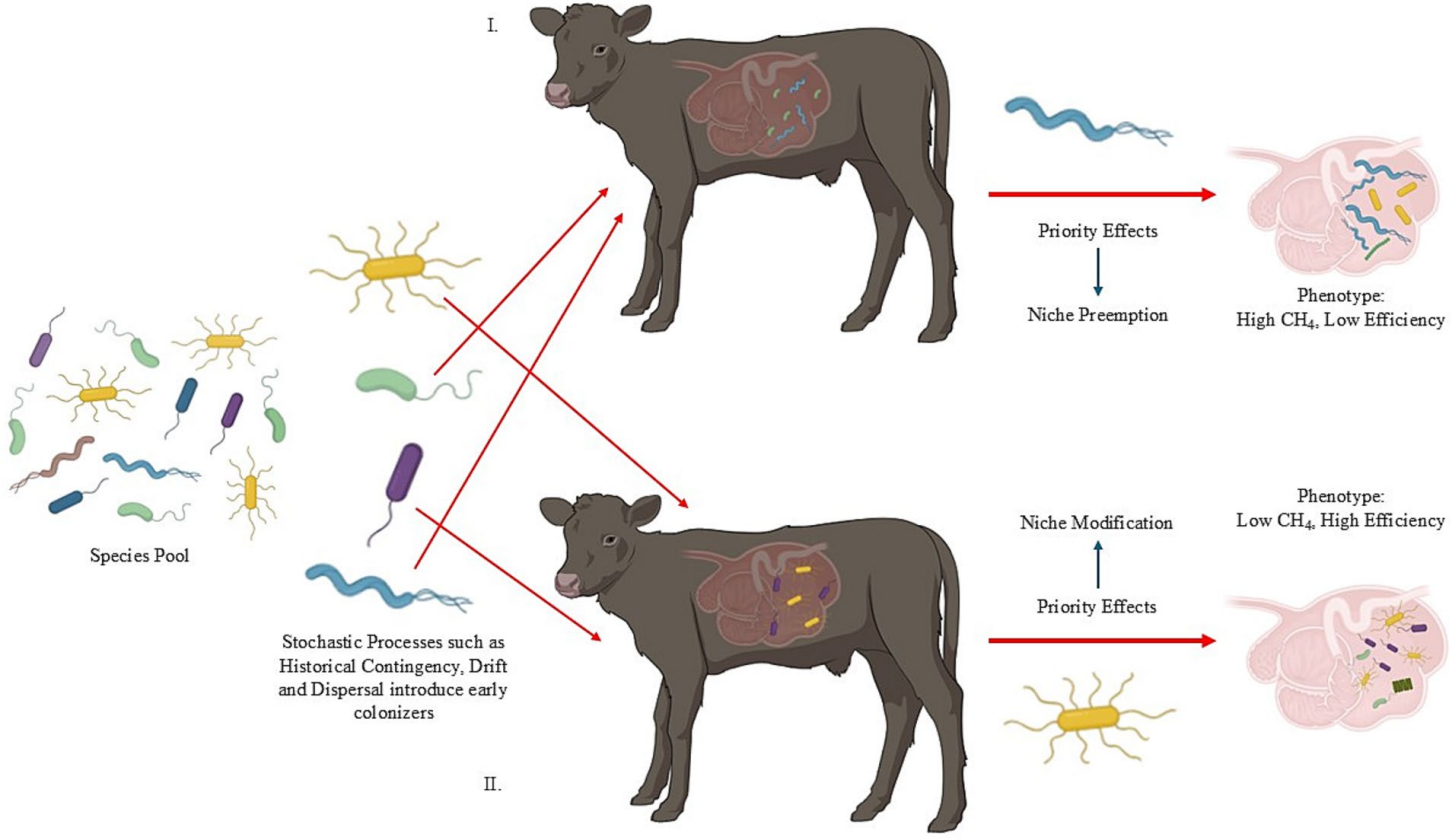
The first microbial colonizers are driven by stochastic processes such as historical contingency, drift, and dispersal. In this example, two scenarios are explored. In scenario I, the early arriving species introduce niche preemption. Here, the blue bacteria inhibit the growth of the green bacteria generating a high CH_4_, low efficiency animal. In scenario 11, the early arriving species introduce niche modification. Here, the yellow bacteria create an environment suitable for colonization and establishment for other microbial members generating a low CH_4_, high efficiency animal phenotype (Adapted from Mizrahi and Jami, 2021).

Stochastic forces (Table 1) are defined as random changes in the community structure that are probabilistic with respect to species identity and/or functional traits (Vellend et al., 2014; Evans et al., 2017; Zhou and Ning, 2017). Stochastic processes such as historical contingency, birth or death events (i.e., drift), and dispersal (i.e., passive) often shape the initial colonization events (Figure 2; Zhou and Ning, 2017; Evans et al., 2017). These unpredictable events often reduce the ability to predict microbial composition. Importantly, early life microbial colonization is partly shaped by random invasion indicated by the vast differences in early life rumen microbial composition (Mizrahi and Jami, 2021). Indeed, evidence reveals community differences between two-day old calves, three- to five-day calves, and six- to 12-day old calves while stating that age-related changes were different taxonomically (Rey et al., 2013); this finding indicates that age, a deterministic driver, could be influenced by stochastic forces. Perturbation or disturbance events such as calf diarrhea further characterize variation in early life microbial composition (Pan et al., 2024). Additionally, treating calf diarrhea with a direct-fed microbial promoted a transition from deterministic forces to stochastic-driven assembly (Pan et al., 2024). These findings suggest that stochastic forces could be more prominent in microbial assembly than originally thought. Thus, stochastic forces are important ecological factors in rumen microbial assembly and could have profound consequences on CH_4_ production.

**Table 1:** Glossary.

Anna Karenina principle	Historical contingency
A hypothesis predicting that certain stressors have stochastic effects on community composition rather than deterministic effects (“all healthy microbiomes are similar; each dysbiotic microbiome is dysbiotic in its own way”)	Events prior to microbiome establishment such as past environmental conditions, species’ arrival order, mutations, and/or population dynamics; these events shape ecological and evolutionary outcomes
Black queen hypothesis	Holobiont concept
A microbial evolutionary theory that describes the idea that stable, positive microbial interactions lead to gene or function loss in an effort to reduce functional redundancy	An evolutionary theory that posits genetic selection acts upon the host along with its microbial partners together
Core microbiome	Hologenome
Microorganisms that are common to a specific microbiome environment found in multiple hosts	The sum of all genetic material from the host and its microbial symbionts
Deterministic forces	Island biogeography theory
In microbial ecology, deterministic effects are those processes that will result in the same community composition given the same initial conditions	Individual hosts or groups of hosts are consider islands and seeks to explain the spatial dynamics affecting dispersal between islands
Dispersal	Keystone species
Movement and successful establishment of organisms across space	A microorganism that has a greater community impact than is estimated by its relative abundance
Drift	Metacommunity theory
Changes to species identity in the relative abundance of various species within the community over time	Identification of interspecies interactions within and between metapopulations
Driver	Metapopulation theory
In this context, a driver is any outside influence (e.g., diet, environment, etc.) that causes changes to microbial population diversity or composition	Microbes spread across a spatial plane identified in patches, metapopulation theory considers the distribution of species among the population patches
Food web/microbial network	Microbial functional groups
The complex interconnections between microorganisms within the same environment or ecosystem and their differing microbial food chains	A cluster of microbes that share or have identical genes encoding for the same function within the ecosystem
Functional group concept	Red queen hypothesis
A microbial ecological approach wherein microorganisms present in each environment are grouped together based on their metabolic inputs and outputs	Proposes an evolutionary arms race in due to perpetual co-evolution resulting from strictly biotic forces
Functional redundancy	Stochastic forces
A characteristic of species within an ecosystem where certain species contribute in equivalent ways to an ecosystem function such that one species may substitute for another	In an ecological and evolutionary context, stochasticity is a random probability of distribution that can be analyzed statistically but may not be predicted with precision

Historical contingency driven by stochastic processes is an important concept in microbial assembly and structure. In this context, historical contingency refers to the effects of past interaction events, either abiotic or biotic, on community assembly while accounting for order and temporal scaling (Fukami, 2015; Zhou and Ning, 2017). Historical contingency is shaped by priority effects—defined as the effects one species has on another depending on the order of arrival and colonization (Fukami, 2015). Priority effects are categorized as either inhibitory or facilitative wherein the early arriving species can negatively or positively affect secondary arriving species, respectively (Jablonski and Sepkoski, 1996; Fukami, 2015). Biotic microbial interactions can determine priority effects through either niche modification (e.g., species A modifies the local environment creating conditions sufficient for colonization by species B; see Jami et al., 2013) or niche preemption (species A depletes resources needed by species B for colonization; see Debray et al., 2022; Debray et al., 2023). These interactions can have short- and long-lasting effects on microbial composition within the rumen environment (Shaani et al., 2018). An example of this within the rumen was demonstrated by Jami et al. (2013), where early aerobic and facultative anaerobic colonizers consumed the total oxygen present and made it possible for anaerobic species to dominate the population. Disruption events at the initial stages of microbial assembly could therefore be major drivers of microbial composition and diversity. In human biology, evidence exists that perturbations, primarily mode of delivery (i.e., C-section vs. vaginal birth), at the time of microbial community assembly, impact digestive system microbiome composition at later stages of development (Bokulich et al., 2016; Yassour et al., 2016).

Other evidence suggests little to no relationship between mode of delivery and microbial population structure (Chu et al., 2017; Stewart et al., 2017). Yet, in ruminants, evidence has suggested that mode of delivery does influence microbial assemblage. The rumen microbiome changes in young animals as they transition from milk to a concentrate-based diet. For example, one study found that in 4-week-old calves, the time when the rumen is transitioning to becoming functional, there were specific differences in rumen microbial profiles between natural born and C-section delivered calves, although they did not differ significantly across all sampling days (Cunningham et al., 2018). This finding suggests that even though immediate microbial composition differences might not be evident, there are long-term effects between vaginal and C-section births on microbial assembly. Furman et al. (2020) more recently demonstrated that calves born vaginally have more homogenous microbiomes and showed that C-section delivered calves are more susceptible to invasion. Additionally, the study showed different microbial profiles associated with either vaginally delivered calves (e.g., *Prevotella* and *Butyrivibrio*) or calves delivered via C-section (e.g., *Peptostreptococcus* and *Dorea*). These studies highlight how priority effects through niche modification or niche preemption are prerequisites for historical context of the rumen microbiome.

Ecological drift is an important concept in community ecology and is defined as changes to species identity in the relative abundance of various species within the community over time because of birth or death events and reproduction (Zhou and Ning, 2017). Drift has been referred to as the only unambiguous stochastic process (Vellend et al., 2014; Zhou and Ning, 2017). Drift plays a more crucial role under weak selection and small, localized communities and has been estimated to reduce diversity and increase compositional distances (i.e., beta-diversity; Fodelianakis et al., 2021). Importantly, rare taxa are more vulnerable to ecological drift events. Given that evidence has suggested that rare taxa disproportionately contribute to temporal changes in microbial diversity (Shade et al., 2014), disruption to these important rare taxa can have long-lasting effects on microbial ecology. These rare taxa can be keystone species—defined as having a greater community impact than estimated from relative abundance (Paine, 1966; Mills et al., 1993). Keystone species tend to have the most links within a particular food web network, and it has been suggested that the loss or gain of these species at initial colonization would result in drastic alterations to microbial community make-up and dynamics (Koskella et al., 2017). In the rumen, *Fibrobacter succinogenes* is a known cellulolytic species with a relative abundance of approximately 0.5% and is considered an important member of the core microbiome, as it fuels the metabolism of other microbial members by producing succinate and soluble sugars from cellulose (Kim et al., 2011; Mizrahi et al., 2021). A recent cohort study determined that *Fibrobacter succinogens* while in low abundance, occurred in all animals enrolled in the study and has been linked to the core heritable microbiome (Wallace et al., 2019), leading to an indication that this species could be a keystone species. Consequently, altering keystone species could impact priority effects, further introducing alternative community compositions (Debray et al., 2022). Moreover, the high functional redundancy seen in the rumen microbiome (see below) permits the population with greater chances for ecological drift (Zhou and Ning, 2017). Thus, drift events in the rumen microbiome environment can have drastic effects on composition and diversity with long-lasting consequences.

Dispersal is fundamental to ecology and evolution (Zhou and Ning, 2017). Defined as movement and successful establishment of organisms across space, dispersal can occur via active or passive mechanisms and is important to understanding enteric CH_4_ mitigation dynamics in ruminant microbial assembly (see below). Active dispersal is a deterministic process such as certain species being better dispersers than others (Nemergut et al., 2013; Lowe and McPeek, 2014; Evans et al., 2017). However, passive dispersal is typically considered a stochastic process, wherein microbes translocate via wind, water or other passive mechanism (Nemergut et al., 2013). Dispersal is often referred to in a manner of limitation due to research suggesting microbes show strong biogeographical patterns (Hanson et al., 2012; Zhou and Ning, 2017). Evidence from human studies show that the lung microbiome supports dispersal limitation as the lung microbiome is an isolated system (Whiteson et al., 2014; Koskella et al., 2017). Given the resilient nature of the adult rumen microbiome, dispersal limitation could apply due to founder effects, a form of genetic drift where the frequency of a given genotype in a population changes due to stochastic properties rather than selection, in which even a fraction of the original microbiome could re-establish itself and prevent the invasion of secondary community members (Weimer et al., 2017; Moraïs and Mizrahi, 2019). Thus, neutral theory could apply in the early life ruminant system as evidence suggests that stochastic properties in microbial composition assembly result in variation within and between ruminant animals in early life (Rey et al., 2013; Jami et al., 2013; Furman et al., 2020; Pan et al., 2024). However, as the ruminant matures, more deterministic, selective pressures (e.g., diet; see below) override neutral processes. Therefore, future work should posit questions on when neutral theory applies, especially in the early life ruminant, and to determine the proper timing of potential microbiome engineering strategies.

### 2.2 Deterministic properties for microbial composition

Deterministic properties influence microbial community assembly in a predictable manner. These properties include diet, age, and host genetics. The stochastic forces that might govern community assembly are often constrained within the strong selective pressures imposed on the animal by deterministic properties (Furman et al., 2020; Mizrahi and Jami, 2021). Furman et al. (2020) explained that diet and animal age often confine the stochastic processes shaping community assembly in ruminants. Therefore, deterministic forces are highly important in shaping ruminant microbial community assembly and dynamics.

Indeed, the nature of selective pressure imposed by deterministic factors produce more pronounced Red Queen (RQ) dynamics. As first described by Van Valen (1973), the RQ hypothesis (RQH) proposes an evolutionary “arms race” in which perpetual co-evolution result from strictly biotic forces. Since then, considerable evidence has mounted that abiotic or stochastic forces do in fact influence microbial community assembly, as discussed previously. As such, macroevolution tends to be viewed through the lenses of either RQH or Court Jester (i.e., speciation, evolution, or extinction only occur due to stochastic changes; Benton, 2009). Yet, in the stripping the RQH to a less restrictive model, RQ dynamics become useful in understanding deterministic microbial biodiversity (Bonachela et al., 2017). For instance, diet is a considerable deterministic and highly controllable factor. Changes in diet across the life stages of a ruminant animal result in predictable changes to microbial composition and rumen function (Mizrahi and Jami, 2021). For instance, Henderson et al. (2015) collected 742 samples from 32 species and sub-species of ruminants and determined that forage-based diets resulted in a higher abundance of unclassified Bacteroidales and Ruminococcaceae and concentrate-based diets revealed a higher abundance of *Prevotella* and unclassified Succinivibrionaceae. In addition, the microbiome structure of forage-fed animals was consistent and distinctly different from concentrate-fed animals. Other studies have shown that changes in dietary protein and carbohydrate content, supplementation of easily digestible carbohydrates, forage preservation, and different types of forage alter rumen microbial ecology (Fernando et al., 2010; Belanche et al., 2012; Huws et al., 2018; Newbold and Ramos-Morales, 2020). Enteric CH_4_ emissions are impacted by the various types of carbohydrate sources (i.e., monosaccharides, disaccharides, starch, etc.) from plant materials indicating that the biochemical pathways in carbohydrate digestion could be influenced by alterations in microbial assemblages (Sun et al., 2019; Beauchemin et al., 2022). Thus, diet as a deterministic selective pressure induces what is known as fluctuating RQ dynamics. Brockhurst et al. (2014) proposed three broad classes of RQ dynamics: fluctuating, escalatory, and chase. Fluctuating RQ describes co-evolutionary oscillations or cycles in which the evolutionary pressures experienced by one species changes due to the other co-evolving species in the community (Brockhurst et al., 2014; Bonachela et al., 2017). In other words, when one ruminant microbial species adapts to changes induced by dietary modulations, other species co-evolve in an opposite direction; when the next dietary change occurs, this can induce another shift along this gradient. Therefore, species are not in a constant “arms race” (escalatory) or “chasing” (chase) each other. Instead, microbial species are adapting an ever-changing environment. Dietary changes that influence ruminant microbial assembly could induce fluctuating RQ dynamics that alter evolutionary rates related to population density (Khibnik and Kondrashove, 1997; Rabajante et al., 2015; Bonachela et al., 2017). However, when metabolite ratio generates inhibition, these evolutionary oscillations are not accompanied by population density (Bonachela et al., 2017). Therefore, dietary changes to enhance ruminant performance or reduce GHG emissions must consider the method in which dietary manipulations are driving selection (population density versus metabolites). Future experiments should implore frameworks such as the one proposed by Bonachela et al. (2017) to investigate the strength of dietary selective pressure and biodiversity. Doing so could enhance the knowledge of implementing dietary changes to select for certain host-microbe phenotypes.

Nevertheless, there is potential where other RQ dynamics could apply. For example, research has suggested that ruminant age independent of diet or sex results in significant clustering effects on microbial assembly (Furman et al., 2020). Age or ruminant developmental stage can therefore be considered a deterministic force for microbial composition. Let us consider the holobiont concept, wherein evolutionary forces act upon both the host and its microbial symbiont, and their combined genomes—termed the ‘hologenome’ (Zilber-Rosenberg and Rosenberg, 2008). As the animal matures, both host genetic factors and microbial genomes are acted upon by selective pressures induced by aging. The immune system matures and becomes more powerful, creating selective pressure on microbes that are more susceptible to host defenses. Therefore, the microbial community selected for is one that is capable of evading or withstanding host immune responses. Additionally, microbes could co-evolve with niches within the rumen as they are modified over time. These scenarios are examples of escalatory RQ dynamics (interaction between host and microbiota result in the so-called “arms race”) or chase RQ dynamics (interacting species evolve in varying directions due to selective pressure), respectively (Brockhurst et al., 2014; Strotz et al., 2018). Past research corroborates this perspective as seen in the composition differences induced by weaning age independent of weaning strategy (Meale et al., 2016; Meale et al., 2017) and by the stability of the ruminant microbiome at just 3 to 4 weeks of age (Rey et al., 2013; Abecia et al., 2014; Guzman et al., 2015; Yáñez-Ruiz et al., 2015). Indeed, age as a deterministic force in microbial assembly should be further investigated through theoretical and empirical models to identify RQ dynamics relative to the holobiont as a unit of selection. The results could provide valuable information for microbiome engineering applications in ruminant production systems.

Host genetics—within species, breed, and sex—have been extensively investigated to link these deterministic factors to the microbiome (Mizrahi and Jami, 2021). Studies have identified differences in microbial profiles between ruminant species (Iqbal et al., 2018), within different breeds of the same ruminant species (Li et al., 2019), and between sexes (Yurkovetskiy et al., 2013; Li et al., 2019). As such, host genetics are an important factor in microbial assembly that can lead to variation between hosts. Interestingly, recent evidence suggests that there is a core microbiome that is heritable and is conserved across numerous ruminant species (Henderson et al., 2015; Wallace et al., 2019). Moreover, while small in terms of species number, the members of the core microbiome contribute to a large proportion of the community relative abundance (Mizrahi and Jami, 2021). This heritable subset of microbes supports several key ecological themes. First, a heritable microbiome implies that the host and its microbiome are symbiotic in nature and therefore, selection acts upon them as a unit—holobiont concept (Zilber-Rosenberg and Rosenberg, 2008; Roughgarden et al., 2017). Second, RQ dynamics could be seen in a subtle fashion as the heritable microbes could play a role in maintaining host fitness against pathogens through outcompeting pathogenic antagonists resulting in host allelic diversity fueling coevolution (Decaestecker et al., 2013). Third, through the lenses of metapopulation theory, metacommunity theory, and island biogeography theory, rumen heritability dynamics can be evaluated (Prosser et al., 2007; Leibold et al., 2004; Koskella et al., 2017; Sarkar et al., 2020; Custer et al., 2022). In metapopulation theory, we consider different “patches” of ruminant microbes spread across a spatial plane and how populations of the same species are distributed among the patches (Prosser et al., 2007). Metacommunity theory on the other hand identifies interspecies interactions within and between metapopulations (Leibold et al., 2004). Alternatively, processes such as social interaction can affect microbial dispersal when treating the individual host as either patches that are habitable by microbes or a group of hosts as a community of various populations and dynamics (Sarkar et al., 2020; Custer et al., 2022). Similarly, island biogeography theory refers to either individual hosts or groups of hosts as “islands” and seeks to address how the spatial dynamics affect microbial dispersal between the islands (Sarkar et al., 2020). In this regard, the ruminant microbial patches, communities, and/or islands are influenced by host genetics and dispersal that can lead to different efficiency phenotypes (Li et al., 2019; McGovern et al., 2020) or perhaps different CH_4_ yield phenotypes (Maman et al., 2020). Therefore, dispersal is treated as a more deterministic factor when evaluating through these theoretical lenses and could impact which microbes are included within a heritable core microbiome. Further research is warranted to elucidate the effects of altering these microbial patches, communities, or islands on ruminant efficiency and CH_4_ emissions.

**Figure 3:**
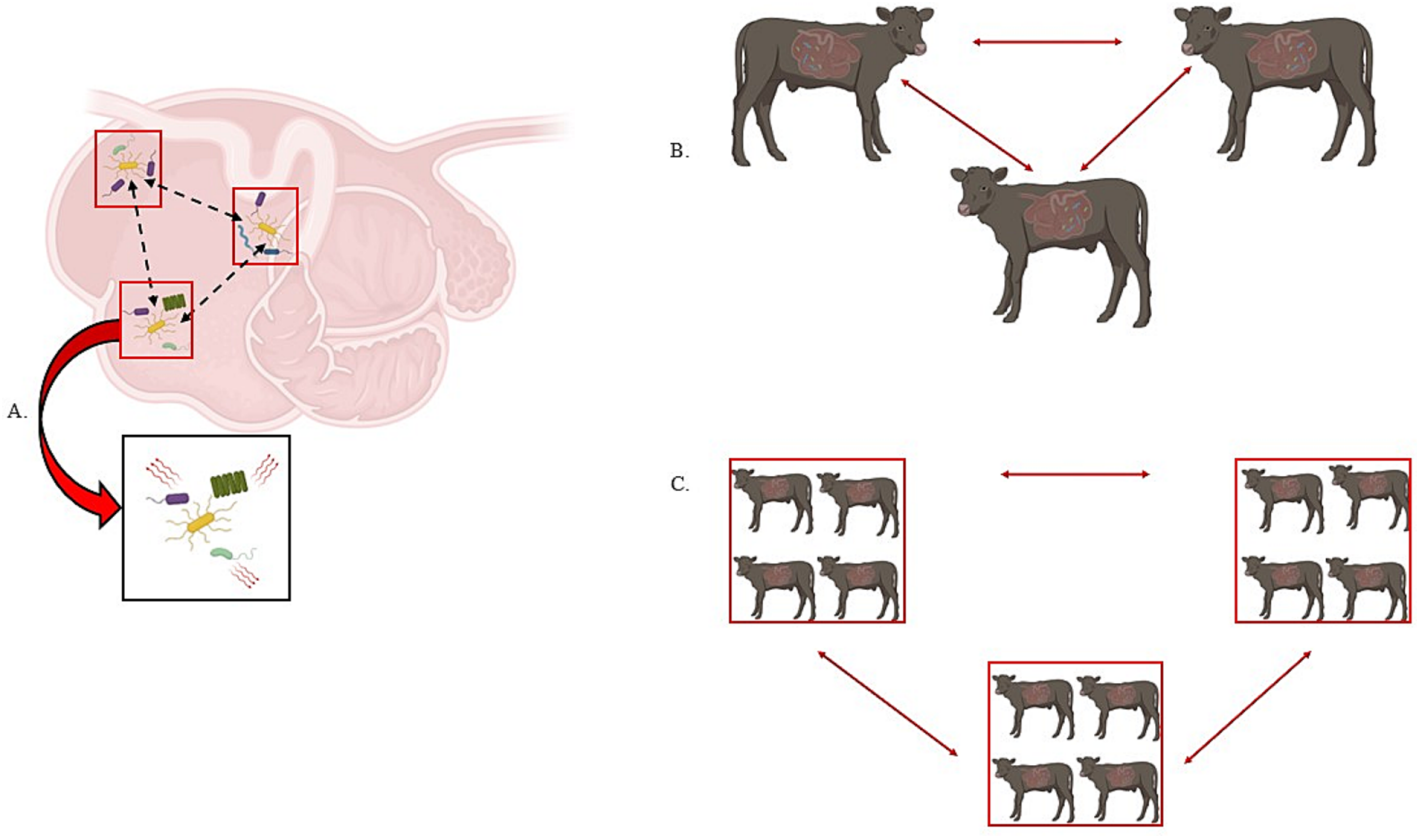
Metapopulation, metacommunity, and island biogeography theories are of interest in ruminant microbial ecology. In panel A, we visualize a metapopulation as three “patches” of microbes (denoted by the red box). The dashed black arrows demonstrate the movement of the yellow bacterial species between the different patches on a spatial gradient – in this case, different areas of the rumen. Additionally, if we zoom into a single patch and can consider interspecies interactions, we incorporate metacommunity theory as the movement of microbes within and between populations. In panel B, we consider the host as the population and the movement of microbes between each host represents metapopulation theory while considering the holobiont. In panel C, we consider a group of hosts as “islands” or “patches” and consider the movement of microbes within and between each island or patch (i.e., island biogeography and/or metacommunity).

In particular, microbial colonization and establishment was thought to occur in a defined and progressive sequence in young ruminants; however, it is now understood that most strict anaerobes dominant in mature animals are actually present in the rumen of animals after only 1 to 2 days after birth, with all major microbial members present by 14 d of age (Fonty et al., 1987; Morvan et al., 1994; Li et al., 2012; Yáñez-Ruiz et al., 2015). Rey et al. (2013) demonstrated that microbial establishment was rapid and sequential in dairy calves—meaning that the dominant bacterial phyla is rapidly replaced by another (i.e., Proteobacteria is replaced by Bacteroidetes as the dominant phyla). By day 15, the microbial communities were achieving stability, as clear temporal patterns were no longer detectable at the phyla level albeit with some variations in relative abundance. Moreover, Yáñez-Ruiz et al. (2015) point out the questions of (1) does rumen development determine which microbes can colonize? or (2) do the microbes shape rumen development through specific signaling? If there is a set of heritable microbes that define community assembly through niche modification or preemption based on priority effects, then the movement of microbes across space and time could be viewed through a metacommunity, metapopulation and island biogeography theoretical framework as discussed above. However, the turnover of genes or species within a given microbiome complicates answering mechanisms of coexistence and heritability (Koskella et al., 2017). Indeed, this could complicate identifying the key microbes involved in community assembly and dynamics (Box 1). Therefore, it is important for future research to address the questions of identity and community assembly with empirical data derived from key ecological and evolutionary concepts (Figure 3).

Box 1 **A born identity**

One of the major challenges to microbiome predictability from animal-to-animal or generation-to-generation is the question of identity. Taxonomy, phylogeny, and functionality are all used to identify members of the given microbial ecosystem. However, in systems such as the rumen where functional redundancy is high, there are blurry lines between taxonomy and functionality. The species concept itself is inconsistent in the microbial landscape and is further hindered by the limited number of culturable species compared to that of data from next-generation sequencing techniques. Additionally, identification is overwhelmed by biological processes such as horizontal gene transfer (HGT) and community mutualism. The exchange of beneficial genes between various species blurs the lines of phylogeny and phenotypic expression (Koskella et al., 2017). While there is evidence that HGT is driven by the specific environment (i.e., rumen vs. distal gut) rather than phylogeny, the addition of perturbations such as antibiotics or CH_4_ mitigation supplements disrupts dynamic neutrality (Smillie et al., 2011). The interactions between species in dynamically changing environment become questionable when attempting to identify the key players in the ecosystem.

A question is therefore raised in using early-life host-associated microbiomes to identify the key members prior to increased diversity as the host ages. In the rumen specifically, a “core microbiome” has been identified across a wide range of hosts and diets (Henderson et al., 2015). There is evidence that the early rumen arrivers become keystone taxa in determining the future of microbial composition (see section on rare taxa). Additionally, the rare taxa that may or may not be keystone taxa have further been identified as disproportionately being causative agents behind temporal changes (Shade et al., 2014). Identification of these rare taxa therefore becomes paramount in understanding life-long composition dynamics and the taxa at the time of birth could represent a key target in untangling functional and taxonomic dynamics. Unfortunately, evidence exists that predicting function from taxonomic identification from early life succession is questionable. A human study in infants revealed that microbial composition did not correlate to functionality, primarily that microbial diversity was not mirrored by functional range (Vallès et al., 2014; Koskella et al., 2017). Thus, functional identity and taxonomic identity could vary even at the time of birth when microbiomes are simplistic making the question of identity even more saturated with uncertainty.

## 3 The ruminant microbiome and ruminant metabolism

### 3.1 Functional redundancy

**Figure 4:**
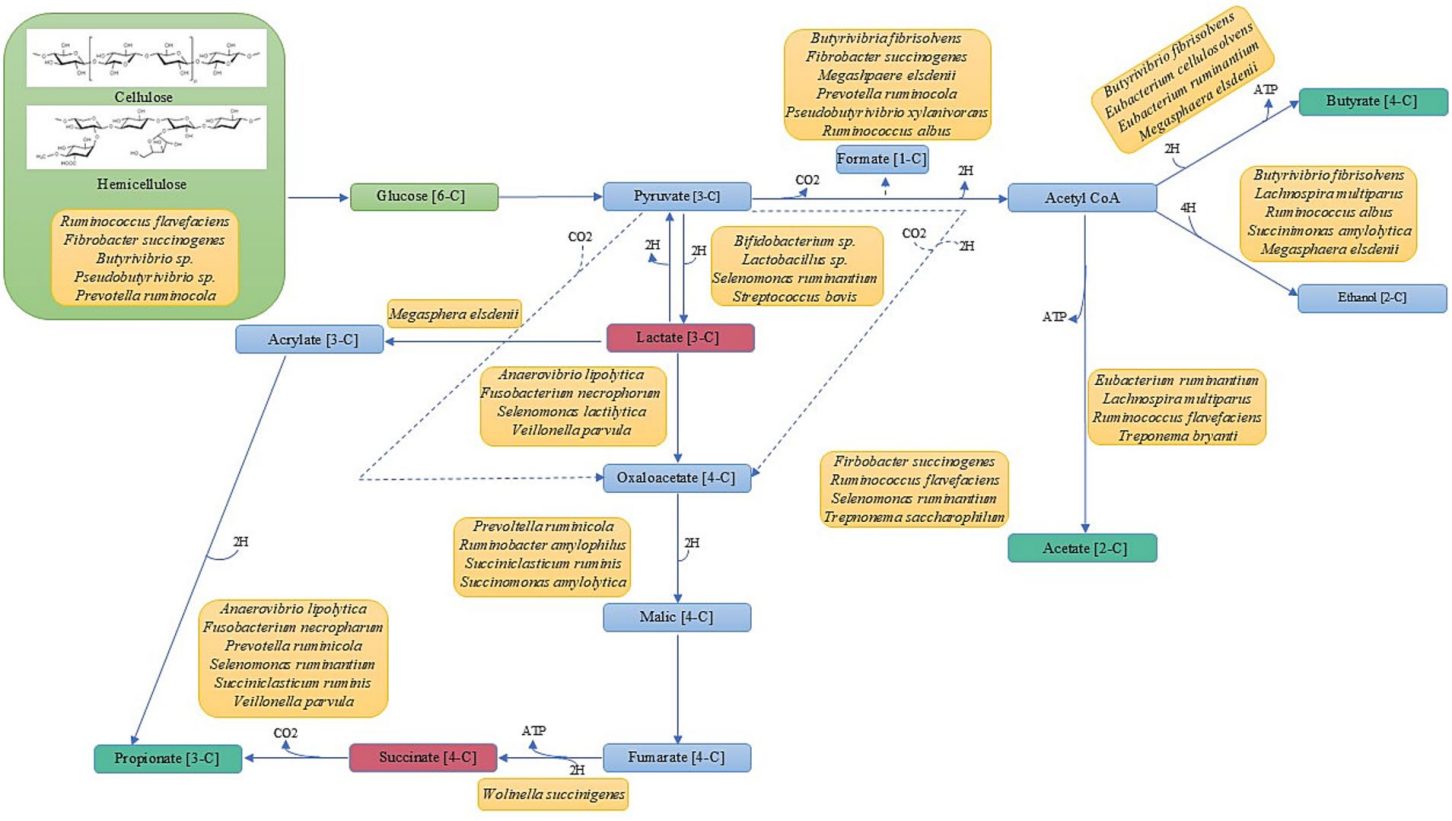
Volatile fatty acid production involves many different microbial species that occupy three different trophic levels. Green represents trophic level 1. Red boxes indicate products involved in level two as end-products and in level three substrates. Teal boxes represent products involved in levels two and three as end-products. Yellow boxes represent known microbial species intricately involved in VFA production (Adapted from Hassan et al., 2020, Mizrahi et al., 2021, and Besharati et al., 2022).

Rumen metabolism and the generation of VFA is conducted by a complex and coordinated microbial community with successive cross-feeding across the different yet interconnected microbial food chains (i.e., food webs), generating three distinct trophic-like levels (Figure 4; Moraïs and Mizrahi, 2019; Mizrahi et al., 2021). Several microbial species are involved in more than one trophic level, indicating the importance of the microbiome in nutrient production for ruminant animals. The first trophic-like level focuses on the degradation and metabolism of cellulose and hemicellulose—the most abundant sugar polymers of the plant cell wall. Here, microbes colonize plant cell wall structures and deconstruct sugar polymers into soluble forms with various glycosyl hydrolase enzymes (Moraïs and Mizrahi, 2019). The second trophic-like level utilizes various pathways such as the Embden-Meyerhoff-Parnas pathway and the pentose phosphate pathway to import the soluble sugars into the microbial cells for the use of hexoses and pentoses, respectively (Matte et al., 1992; Hackmann et al., 2017). Many of these sugars are then subjected to fermentation through AD, resulting in the production of organic acids and VFA as well as other metabolites and gases such as CO_2_ and H_2_ (Mizrahi et al., 2021). Finally, the third trophic-like level involves the metabolites lactate, succinate, H_2_, and CO_2_ being further metabolized into propionate, butyrate, acetate, and CH_4_.

Within these trophic levels, there is evidence of high levels of functional redundancy among the members of the ruminant microbiome (Weimer, 2015). In the context of the functional group concept (McGill et al., 2006), microbial functional groups share a specific metabolic function. Moraïs and Mizrahi (2019) describe in detail the benefits of implementing the functional group concept to describe and comprehend the rumen ecosystem, as this approach describes both an ecosystem’s functionality and composition. For instance, in methanogenesis, there are three different functional groups with varying input metabolites scattered among large phylogenetic distances, which produce only CH_4_ as an output metabolite (Janssen and Kirs, 2008; Hook et al., 2010; Enzmann et al., 2018), indicating functional redundancy within the rumen ecosystem. While there is a general lack of understanding on the role and importance of functional redundancy within the rumen, recent studies have shown that only a small proportion of the core ruminant microbiome is conserved among 90% of animals (Wallace et al., 2019) and that the functions encoded by various species and taxonomic identities are far more conserved (Taxis et al., 2015; Mizrahi et al., 2021). This evidence suggests that while these organisms are of high importance to the host’s metabolism, taxonomic variability could mask the functional similarities between various species and genera. For example, consider two microbial species A and B, and species A and species B both encode the same functional gene. However, species A outnumbers species B 10:1. In this scenario, the functional mechanisms of species B could be masked simply by species A being greater in number. Within ruminant archaeal species, functional redundancy was observed between the methylotrophic *Methanobrevibacter* and the *Methanosphaera* species in the competition for methanol and H_2_ to produce CH_4_ (Söllinger et al., 2018). This finding suggests that the rumen ecosystem functionalities are carried out by various species that perform the same or similar functions (Mizrahi et al., 2021). Now, consider a second example where two ruminant animals have different microbiome compositions however, both animals have equal fermentative and productivity outcomes. This example highlights the fact that despite taxonomic and compositional differences between ruminants, functionality remains the same. Taxis et al. (2015) provides evidence for taxonomic masking as they concluded that two steers were functionally similar yet taxonomically varied while being under the same environmental conditions and fed the same diet. Therefore, a primary concern with functional redundancy is that it could lead to the establishment of an ecosystem containing differing compositions, albeit with the same functionality, which could confound research designed to draw conclusions across ecosystems, host ruminants, and studies. Yet another concern is the apparent disconnect between functionality and species identified, which is exacerbated by the outdated notion that microbes that are tightly associated with an animal’s digestive tract are likely to excrete, transcribe or otherwise dislocate genes perceived by the community as being functionally redundant (Koskella et al., 2017). Possible explanations for this community feature are relaxed selection, genetic drift, or positive selection—as detailed by the Black Queen Hypothesis (BQH) wherein stable, positive microbial interactions lead to the loss of genes or function to reduce functional redundancy (Morris et al., 2012; Koskella et al., 2017).

**Figure 5:**
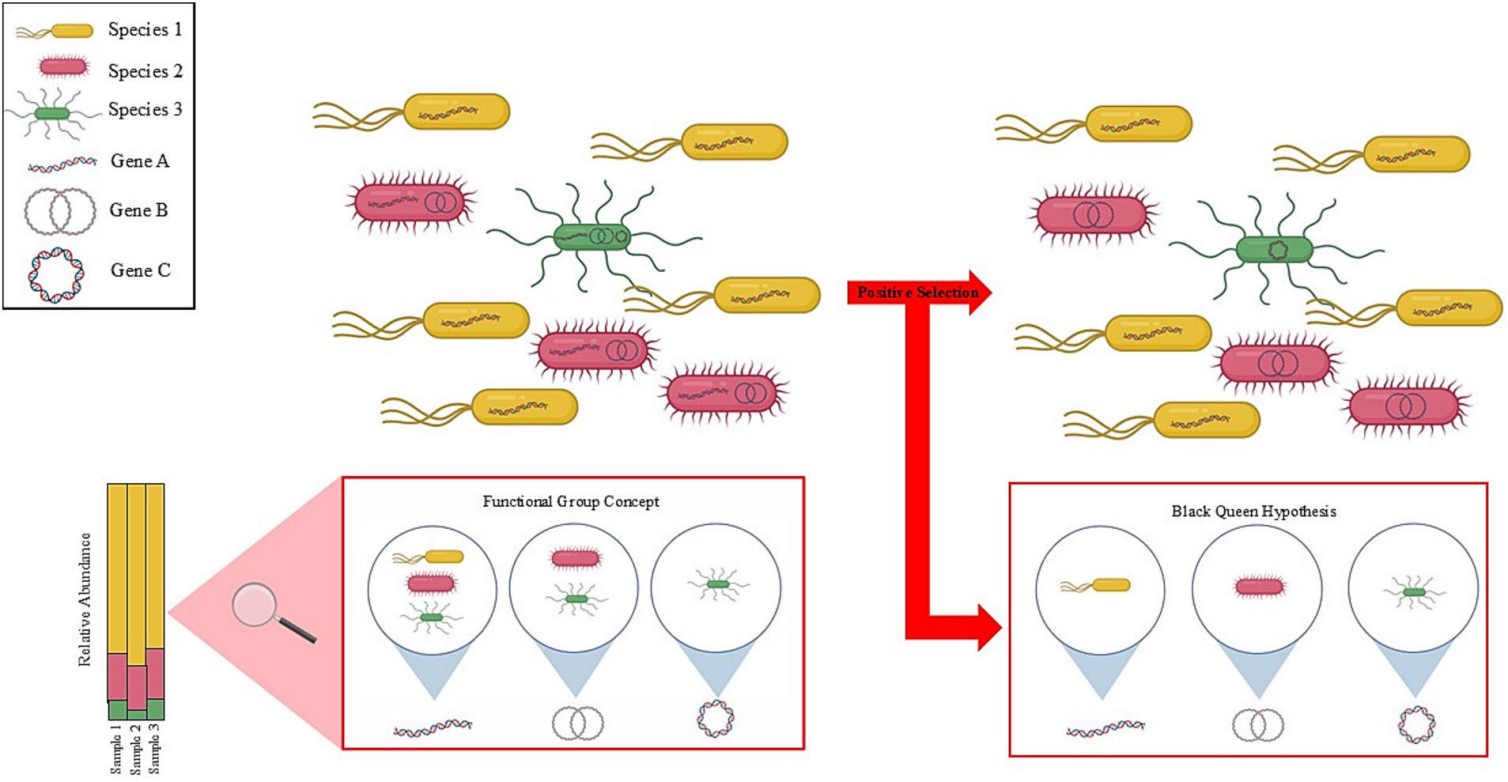
The blurry lines between taxonomy and function. Functional redundancy in the rumen ecosystem arises due to variability in microbial species and their taxonomic identity. Taxonomic analysis could reveal that Species 1 (yellow) is greater in relative abundance compared to Species 2 (pink) and Species 3 (green). Functional analysis could mask the species in lower abundance making taxonomic analysis of the microbiome difficult. Meanwhile, applying the functional group concept enables the various species to be grouped together based on functional genes. In doing so, Species 1, Species 2, and Species 3 are now considered to be “the same” according to function for Gene A. This process can further be applied by grouping microbes for Gene B and Gene C. Another consideration regarding function is that a stable community could be acted upon by positive selection resulting in a loss of genes to reduce functional redundancy (i.e., BQH). Here in this example, Species 2 and Species 3 would lose Gene A because Species 1, which is more abundant, carries the gene. Additionally, Species 3 would lose Gene 2 as Species 2 is more abundant. Therefore, the function encoded by Gene A would still be carried out by Species 1 and the remaining community members are now free to perform other community functions that could be beneficial to its host. Indeed, the same logic goes for gene loss in Species 2 and Species 3. The functional group concept and the BQH are important ecological and evolutionary concepts for understanding microbial community dynamics that could factor into methanogenesis inhibition interventions.

The concepts of functional grouping and BQH within the ruminant microbial system are intriguing in that metabolism in the rumen ecosystem is generally consistent with functionality (Flint, 1997; Mizrahi, 2013). However, the specific species identity related to functional annotation can vary distinctly from study to study, animal to animal, diet to diet, making meaningful taxonomic inferences incredibly difficult. Studies have highlighted this phenomenon by comparing rumen metagenomics data with taxonomic annotation databases, demonstrating similarities at the metabolic level between taxonomically distinct microbial communities (Taxis et al., 2015). These similarities in function among taxonomically distinct microbial populations can potentially be explained by both the functional group concept and the BQH (Figure 5). In another example, two different strains of the same species can have different functionalities (Cohan, 2006; Koeppel et al., 2008). This indicates that species functionality is not ubiquitous among systems; furthermore, positive selection could be reasonably applied if the function is absent or inactive in one of the strains but is present by another species within the community. This example could be indicative of the functional group concept wherein species with differing function, yet taxonomically similar, are now considered “different,” based on overall function and not taxonomy. The BQH would posit that the species in question could be in the presence of other species capable of producing the same genes/function and have lost the ability to produce those duplicate genes/function to improve community fitness. Therefore, metagenomic approaches should be a research priority to investigate functional genes in the rumen microbiome. Alternatively, genetic transfer across organisms could alleviate the need for taxonomic microbe diversity in favor of a more functional diversity. Moving away from taxonomic diversity toward functional diversity could also provide information about environmental and host-derived drivers of microbial diversity. Metagenomic approaches may confound knowledge gained from earlier studies, with those of more modern works. Considerable effort should be used in formulating highly reproducible results with few differences in diet and animal sex, age, and weight, via quantifiable ‘old school’ hand microscopy and quantifiable studies (done in triplicate). The combination of these two techniques provides a synergistic effect and makes for simpler analyses, which are necessary to fully apply current ecological and evolutionary theory to the microbial communities involved in ruminant microbial metabolism.

### 3.2 Methanogenesis and methane mitigation

Of the major metabolic processes in the rumen, methanogenesis is of considerable interest. Mediated by methanogens, methanogenesis is an important metabolic process for the removal of H+ following the fermentation of foodstuffs eaten by the ruminant. Plant cell wall polysaccharides are broken down by fibrolytic microorganisms into VFA, CO_2_, and H_2_. To avoid a high H_2_ partial pressure, H_2_ is quickly utilized by hydrogenotrophic microorganisms, primarily methanogens (Pereira et al., 2022). Accumulation of H_2_ has been posited to have detrimental effects on the fermentation process due to high H_2_ partial pressure within the rumen inhibiting certain microbial dehydrogenases which reduce fermentation, dry matter intake (DMI), and the digestibility of feeds (Janssen, 2010; Leng, 2014). Thus, within the rumen, methanogens play a key role by acting as a H_2_ sinks with CH_4_ production representing the largest H_2_ sink in the ruminant digestive tract (Beauchemin et al., 2020).

Methanogens can be traced back to the Archaean period, more than 2,500 million years ago (Gribaldo and Brochier-Armanet, 2006). It has been suggested that the last common ancestor for archaea was indeed a methanogen (Burggraf et al., 1991; Bapteste et al., 2005). While separated by vast phylogenetic distances, all methanogens are alike in their ability to utilize various metabolites to produce CH_4_ in the last step of AD (Friedman et al., 2017). Indeed, the rumen is an anerobic environment that is specialized for methanogenic growth. As a holobiont, the methanogenesis process is an evolutionary process that uses methanogens for rumen H_2_ maintenance resulting in the release of CH_4_. Therefore, it is not a surprise then that methanogens and their host experience co-evolutionary forces when faced with selective pressures such as changes in diet (Fernando et al., 2010; Belanche et al., 2012; Huws et al., 2018; Newbold and Ramos-Morales, 2020), animal management (Xiang et al., 2016; Roehe et al., 2016; Martinez-Álvaro et al., 2020; Maman et al., 2020; Meale et al., 2021), or CH_4_ mitigating feed additives (Roque B M, et al., 2019; Pitta et al., 2022). Due to the thermodynamic favorability of methanogenesis, an important question arises: how do livestock producers and researchers address CH_4_ emissions. With climate change at the forefront of many research efforts, CH_4_ mitigation in ruminant livestock has been thoroughly investigated and reviewed (Huws et al., 2018; Abbott et al., 2020; Beauchemin et al., 2020; Mizrahi et al., 2021; Yu et al., 2021; Min et al., 2021; Bačėninaitė et al., 2022; Beauchemin et al., 2022; Glasson et al., 2022). A detailed review of these efforts is also provided in the Supplementary material. While many of these methods show promise in CH_4_ mitigation, important questions remain for microbial ecology.

**Figure 6:**
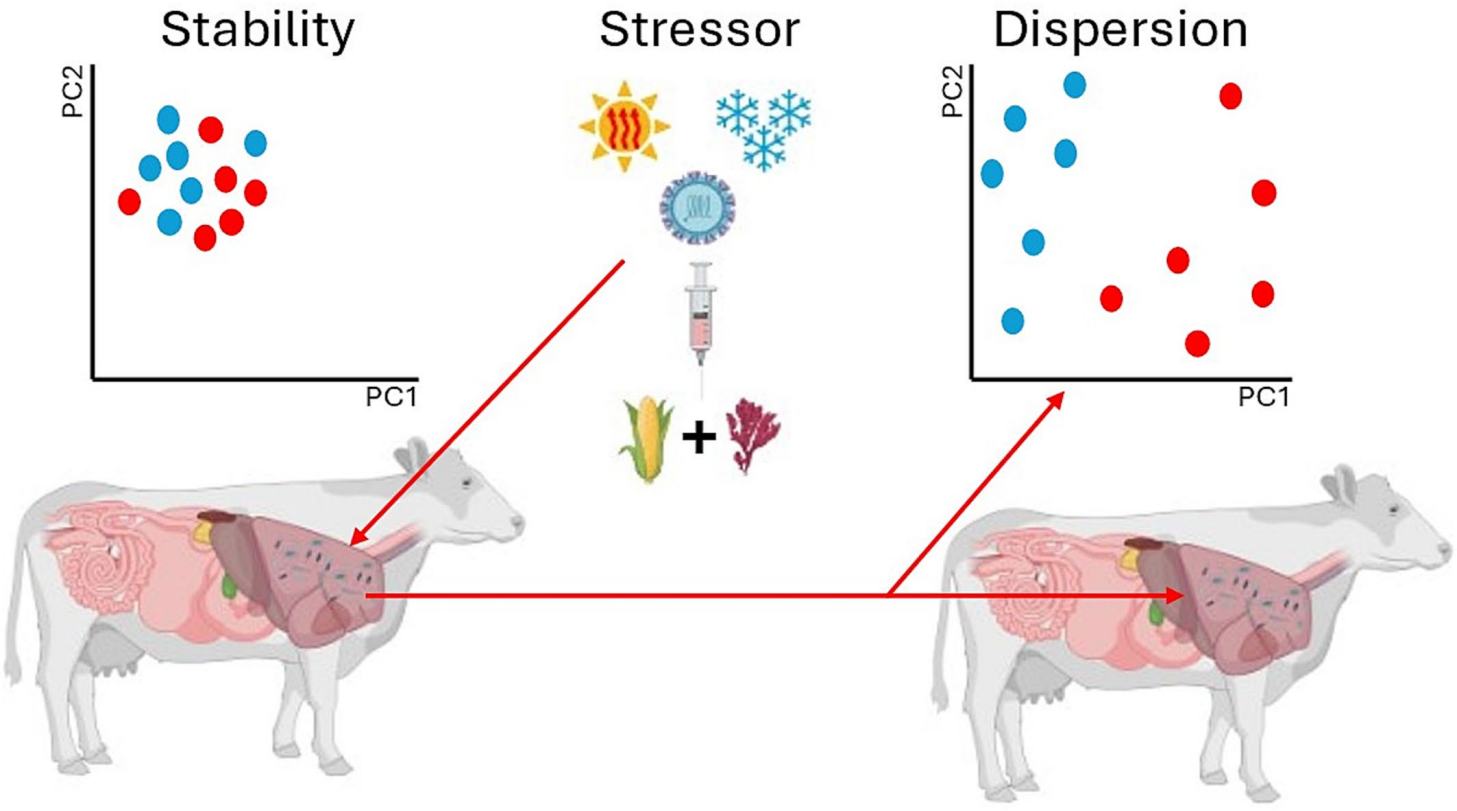
Dispersion of microbial communities can be initiated by many factors. Deterministic factors such as dietary interventions and stochastic forces such as environmental stressors can act to alter or disrupt stable microbiomes. Anna Karenina principles demonstrate that these variability changes are often stochastic in nature as deterministic forces are more localized.

One consistent finding among these works, however, is that some form of microbial dispersion (either stochastic or deterministic) is seen. Research has reported microbial changes in diversity (for example Snelling et al., 2019), composition (for example Roque B M, et al., 2019), and metabolic function (for examples see Ben-Shabat et al., 2016 and Li et al., 2020) when applying CH_4_ mitigation strategies to ruminant models. Beta-diversity (*β*-diversity), or microbial community composition, is of particular interest in microbial ecology. While *β*-diversity has historically been defined as the turnover of species between samples (Whittaker, 1972), it has also been used in ruminant livestock studies to measure temporal changes (for examples see Clemmons et al., 2019; Snelling et al., 2019; O’Hara et al., 2020), health status (for examples see Frazier, 2021; Hu et al., 2022), and CH_4_ emissions and mitigation (for examples see Roque B M, et al., 2019; Meale et al., 2021). Community composition is often visualized using methods such as non-metric multidimensional scaling (NMDS) or principal coordinates analysis (PCoA) which allows for two- or three-dimensional representation of multidimensional data (Zanevald et al., 2017). Within these analyses and subsequent visualizations, community composition is represented by clustering, and dispersion among clusters, or “smearing,” often correlates to differences in health, temporal changes, and other perturbation events such as CH_4_ mitigation strategies that might alter microbial ecology (Figure 6). However, it is important to recognize that these correlations might not indicate the full dynamic possibilities of how the microbiome contributes to changes within the host.

The “smearing” effect on microbiomes could be due to stochastic changes rather than deterministic changes. Stochastic changes induced by stressors alter otherwise stable microbiomes producing a constrained “core microbiome” surrounded by a “smear” of distressed microbes (Figure 6). These observations ultimately translate to Anna Karenina principles (AKP) within host-associated microbiomes. The AKP concept is derived from Leo Tolstoy’s novel entitled Anna Karenina. The first line of the novel reads “*Happy families are all alike; every unhappy family is unhappy in its own way*” and it was made popular for biology by Diamond (1999). Translated into host-associated microbiome AKP, the concept mirrors Tolstoy’s work by stating “all healthy microbiomes are similar; each dysbiotic microbiome is dysbiotic in its own way.” Zanevald et al. (2017) discusses AKP in animal microbiomes in depth and provides the hypothesis that AKP predicts that certain stressors cause stochastic effects (dispersion effects) rather than deterministic effects (location effects). That is, stochastic stressors increase variability within a given microbiome rather than deterministic, creating distinct local clusters. Past research has indicated AKP indeed occur when using *Asparagopsis taxiformis*, a red algae capable of inhibiting CH_4_ up to 99%, as a dietary mitigation strategy (Roque B M, et al., 2019). The local clusters created by AKP can lead to divergent community assemblages making it more difficult to predict the outcomes of CH_4_ mitigation. These altered community states often display resistance to change due to ecological feedback systems unless perturbations are strong enough (Moraïs and Mizrahi, 2019). Thus, mitigation strategies could prove difficult in the face of stochastic properties of assembly due to AKP and neutral theory.

Nevertheless, evidence in other systems suggests microbial assembly following perturbations follow non-neutral processes (Stein et al., 2013; Koskella et al., 2017). Similarly, ruminant nutrition is an important deterministic factor in microbial assemblage as previously discussed. There is significant potential in utilizing diet or dietary manipulations to reduce enteric CH_4_ emissions. These methodologies such as utilizing higher quality forages (Beauchemin et al., 2008; Beauchemin et al., 2020; Beauchemin et al., 2022) or increasing concentrate levels within ruminant diet (Thompson et al., 2019; Snelling et al., 2019) to reduce CH_4_ emissions unsurprisingly result in different microbial compositions (Wang et al., 2020). Additionally, using dietary additives such as 3-NOP or sunflower seed oil along with higher concentrate diets both increase CH_4_ reduction and alter microbial ecology (Bayat et al., 2017; Schilde et al., 2021). However, these deterministic mitigation forces are often constrained by the resilient nature of the rumen microbiome. Indeed, research has shown the ability of the rumen to return to pre-intervention microbiome states and host phenotypes following rumen content exchange between low and high CH_4_-emitting animals (Weimer et al., 2017; Moraïs and Mizrahi, 2019). At the community level, resilience is best defined as the rate of the community’s ability to recover to pre-perturbation function rather than pre-intervention assembly (Song et al., 2015). This particular definition is important in an environment such as the rumen where there are high levels of functional redundancy. As previously stated, several microbial species within the rumen often perform the same metabolic function separated by vast phylogenetic distances. In a scenario where rumen content exchange occurs, pre-intervention microbiomes could create conditions sufficient that if any fraction of the community remains, a return to pre-perturbation ecology is likely. Thus, founder effects mediated by niche modification could decrease the longevity of CH_4_ mitigation methods (Moraïs and Mizrahi, 2019). It is therefore essential that future research answer the question of how resilient the rumen microbiome altered community states are to intervention methodologies.

## 4 Future directions

### 4.1 A conceptual framework for empirical studies

There are limitations to current ecological and evolutionary theory. The challenge in addressing these limitations is acquiring adequate empirical data to support the many theories that are out there. In the context of ruminant CH_4_ emissions, mitigation strategies, and microbial ecology, it is important that future research studies address these gaps between theoretical and empirical data. In doing so, it is possible to enhance current on-farm CH_4_ techniques by allowing a better understanding of the key microbial processes leading to a high- or low-CH_4_ producing phenotype. Furthermore, application of theory to future studies could enhance our understanding of genomes as genomic data’s importance cannot be understated. However, future empirical studies on CH_4_ mitigation and the microbiome should be grounded in an effective theoretical framework that integrates multiple ecological and evolutionary concepts. These studies should aim to address the interactions between microbial communities, CH_4_ production, and the influence of mitigation on both the host and its microbial partners.

To accomplish this, we provide a conceptual theoretical framework to be used and evaluated for future empirical studies investigating CH_4_ mitigation methods on ruminant microbial ecology. The conceptual framework could provide researchers with a roadmap for future research projects in addressing enteric CH_4_ and anthropogenic GHG emissions. These future studies should integrate theoretical concepts into the hypotheses and study design.

1) The Holobiont Concept: research should focus on how CH_4_ mitigation studies alter the microbial ecosystem and the host’s health and performance. Changes to methanogen abundance could alter emissions resulting in differing digestive phenotypes. These changes should be monitored by investigating fiber digestion, nutrient absorption, and energy requirements.
2) Red Queen Dynamics: empirical studies should discern how CH_4_-inhibiting interventions such as dietary manipulations or feed additives effect co-evolutionary dynamics, particularly those microbial species involved in fermentation. FRQ dynamics could be observed by monitoring population shifts on a temporal and spatial scale.
3) Black Queen Dynamics and Functional Redundancy: functional redundancy and positive genetic function loss need to be studied to determine the effect of reducing specialized communities such as methanogens on host maintenance and overall microbial community assembly. It is important to elucidate if other H_2_ sinks or utilizing species are capable/available to maintain H_2_ pressure within the rumen. Studies should aim to answer if reducing methanogens leads to a reduction in fermentation efficiency or nutrient uptake. Furthermore, studies should not only measure CH_4_ output, but also the overall H_2_ dynamics relative to the microbial community shifts due to mitigation strategies.
4) Resilience and Stability Theory: the resilience of the microbial community should be assessed by observing the changes in microbial populations overtime. Here, microbial ecology can be monitored to determine how quickly the rumen microbiome “recovers” from CH_4_ mitigation strategies. Studies should investigate diversity, functional outcomes, and CH_4_ production on a temporal scale for evidence of new altered community states or a return to pre-intervention assemblages.
5) Metapopulation and Island Biogeography Theory: future empirical studies should consider spatial distribution of methanogens and other key microbial species across the differing “patches” or “islands.” Mitigation techniques could result in localized extinctions or migrations. It is important to evaluate how methanogens recolonize after being targeted and suppressed by mitigation techniques and to determine if other species migrate to colonize the niche methanogens ultimately leave behind.
6) Neutral Theory: research should account for the stochastic nature of rumen microbial assembly. Study designs should incorporate methodologies that account for the variability in microbial community responses to CH_4_ mitigation strategies, especially in early-life ruminants. Replication across animal systems could prove beneficial in disentangling stochastic forces from the more deterministic forces in microbial community ecology.
7) Eco-Evolutionary Dynamics: the interplay between ecological changes (i.e., dietary shifts) and evolutionary processes (i.e., microbial adaptation to the new environment created by dietary shifts) should be analyzed for long-term effects on rumen microbial ecology and evolution. Long-term effects could be tracked by identifying genetic adaptions in methanogens, other keystone microbial species, or H_2_-utliziling competitors. Mitigation techniques could alter the evolutionary traits of the holobiont especially if utilized in breeding and genetic programs for low CH_4_-emitting phenotypes.

### 4.2 Areas of interest for future empirical research

As previously mentioned, empirical research is required to test the many microbiological ecological and evolutionary theories. There is a concerted effort to elucidate the properties of the microbiome that could impact climate change through the inhibition or reduction of ruminant CH_4_ emissions. However, there is a clear knowledge gap for empirical data to address should seek to address these issues by incorporating theory into research by using the conceptual framework provided above. For instance, longitudinal and cross-generational studies could be designed to follow microbial community changes over time by tracking short- and long-term responses to CH_4_ mitigation strategies (eco-evolutionary dynamics); additionally, heritable changes to the microbiome could be revealed as well as how methanogens react to mitigation tactics (BQH). Studies have begun in this area where a longitudinal meta-omics approach investigated the temporal rumen microbiome dynamics and functional redundancy during plant biomass degradation in dairy cattle (Söllinger et al., 2018). Comparative analyses across diet and diet type can provide key evidence on how deterministic forces shape CH_4_ production and how the microbiome responds to different environmental pressures (holobiont concept). However, study designs are needed to elucidate how evolution of the rumen microbiome is affected by mitigation strategies (RQH). To accomplish this, studies should seek to specifically manipulate RQ dynamics. Specifically, CH_4_ inhibitors or feed additives such as 3-NOP or *A. taxiformis* could be used in fluctuating patterns to mimic FRQ dynamics while monitoring microbial adaptation across multiple cycles of dietary changes. This type of study will be essential in furthering our understanding of how early-life interventions could impact ruminant microbial evolution and ecology. Additionally, performing meta-analysis type studies can help standardize methodologies that otherwise would result in cross-study variation (Frazier et al., 2024). This approach can help make between study comparisons more feasible and could reanalyze past studies under the conceptual framework provided herein. Furthermore, sound studies grounded in both empirical and theoretical support could lead to enhanced microbial biotechnologies to reduce CH_4_ emissions (Frazier and Yang, 2023). Taken together, these studies could increase our capabilities in reducing CH_4_ emissions from livestock operations. Additionally, these studies could also help address current limitations of theory such as timescales, the question of identity, heritability, and host-associated control of the microbiota (Koskella et al., 2017).

An area of interest that is currently not receiving significant attention in the ruminant livestock scientific community, is the utilization of methanotrophs as natural CH_4_ biocontrol agents. Methanotrophy as previously mentioned was a primary source of microbial energy in early Earth’s history due to the scarcity of oxygen within the atmosphere (Gribaldo and Brochier-Armanet, 2006). Organisms known as methanotrophs oxidize CH_4_ to gain energy under both aerobic and anaerobic environmental conditions (see Supplementary material; Rani et al., 2024). Past research has indicated that methanotrophs have been shown to be native to the rumen and thus provide a natural system in mitigating enteric CH_4_ emissions (Mitsumori et al., 2002; Finn et al., 2012). Within anaerobic conditions such as the rumen, methanotrophs utilize various terminal electron acceptors such as sulfate, nitrate, nitrite and metals to oxidize CH_4_ (Soren et al., 2015; Ahmadi and Lackner, 2024; Rani et al., 2024). However, little work has been done to elucidate the effectiveness of anaerobic methanotrophs to reduce CH_4_ within ruminant livestock. Interestingly, Finn et al. (2012) suggests that CH_4_ emissions can be reduced by methanotrophs without drastically altering native microbial populations. Thus, there are intriguing questions surrounding the ecological and evolutionary importance of methanotrophs within the rumen environment. For example, RQD could be observed between methanogens and methanotrophs under differing availabilities of H_2_. As previously mentioned, dietary changes can drive selection through population density or metabolite availability (Bonachela et al., 2017). If CH_4_ reduction is being selected for by mitigation strategies, the availability of methanotrophs could be reduced, altering microbial populations locally (i.e., patches/islands) or throughout the rumen microbial system. Moreover, if the substrates and metabolites utilized by both methanogens and methanotrophs are similar, then methanogenesis might be reduced (Jeyanathan et al., 2014). These changes should be investigated to determine the strength of selective pressures such as diet on how resource availability influences methanotrophic communities and if RQD are indeed observable in this context. However, there is lack of *in vivo* studies utilizing methanotrophs for enteric CH_4_ mitigation making theoretical predictions difficult (Tseten et al., 2022). Therefore, future research in this area is warranted and could provide key clues to the evolutionary and ecological impacts methanotrophs have on ruminant environments.

## 5 Conclusion

Enteric CH_4_ from livestock operations, primarily from beef and dairy cattle, is a critical concern for total global greenhouse gas emissions. Undeniably, if left unchecked, enteric CH_4_ emissions represent a major anthropogenic contributor to global climate change. It becomes imperative to reduce the amount of enteric CH_4_ escaping into the environment from ruminant animals. However, the entangled mechanisms found with the rumen microbial interaction networks makes this process extremely challenging for ruminant livestock producers. There are several reasons that make methanogenic inhibition challenging due to the intertwined nature of the ruminant microbiome. One of those reasons is the highly resilient nature of the adult microbiome. The fact that the adult microbiome can return to a pre-perturbation ecology following a negative-impacting event makes CH_4_ mitigation strategies difficult to maintain. Additionally, negatively impacting the native microbiome in the ruminant may lead to hindered animal performance. Current research investigating both the mode of action and the effects of CH_4_ mitigation strategies on CH_4_ output have been conducted and have been thoroughly reviewed. Herein, we provided insights from current ecological and evolutionary theory to help guide future researchers in understanding the ruminant microbiome and its role in CH_4_ production. Empirical studies often lack in theoretical foundations limiting the long-term increases in knowledge of microbial ecology. Additionally, empirical studies grounded in theoretical hypotheses should further explore natural microbiome manipulations for CH_4_ inhibition, such as methanotrophs or early-life interventions. These foundations are needed to fully realize the capability of CH_4_ mitigation strategies within the ruminant microbial environment.

## References

[ref1] Abbott D. W. Aasen I. M. Beauchemin K. A. Grondahl F. Gruninger R. Hayes M.. (2020). Seaweed and seaweed bioactives for mitigation of enteric methane: challenges and opportunities. Animals 10:2432. doi: 10.3390/ani10122432, PMID: 33353097 PMC7766277

[ref2] Abecia L. Ramos-Morales E. Martínez-Fernandez G. Arco A. Martín-García A. I. Newbold C. J.. (2014). DR feeding management in early life influences microbial colonisation and fermentation in the rumen of newborn goat kids. Anim. Prod. Sci. 54:1449. doi: 10.1071/AN14337

[ref3] Ahmadi F. Lackner M. (2024). Recent findings in methanotrophs: genetics, molecular ecology, and biopotential. Appl. Microbiol. Biotechnol. 108:60. doi: 10.1007/s00253-023-12978-3, PMID: 38183483 PMC13497199

[ref4] Aryee R. Mohammed N. S. Dey S. B. A. Nadendla S. Sajeevan K. A. Beck M. R.. (2024). Exploring putative enteric methanogenesis inhibitors using molecular simulations and a graph neural network. bioRxiv [Preprint]. doi: 10.1101/2024.09.16.613350, PMID: 39345548 PMC11429904

[ref5] Bačėninaitė D. Džermeikaitė K. Antanaitis R. (2022). Global warming and dairy cattle: how to control and reduce methane emission. Animals 12:2687. doi: 10.3390/ani12192687, PMID: 36230428 PMC9559257

[ref6] Bapteste E. Brochier C. Boucher Y. (2005). Higher-level classification of the Archaea: evolution of methanogenesis and methanogens. Archaea 1, 353–363. doi: 10.1155/2005/859728, PMID: 15876569 PMC2685549

[ref7] Bayat A. R. Ventto L. Kairenius P. Stefański T. Leskinen H. Tapio I.. (2017). Dietary forage to concentrate ratio and sunflower oil supplement alter rumen fermentation ruminal methane emissions, and nutrient utilization in lactating cows. Transl. Anim. Sci. 1, 277–286. doi: 10.2527/tas2017.0032 32704652 PMC7205341

[ref8] Beauchemin K. A. Kreuzer M. O. O’Mara F. McAllister T. (2008). Nutritional management for enteric methane abatement: a review. Aust. J. Exp. Agric. 48, 21–27. doi: 10.1071/EA07199

[ref9] Beauchemin K. A. Ungerfeld E. M. Abdalla A. L. Alvarez C. Arndt C. Becquet P.. (2022). Invited review: current enteric methane mitigation options. J. Dairy Sci. 105, 9297–9326. doi: 10.3168/jds.2022-22091, PMID: 36270879

[ref10] Beauchemin K. A. Ungerfeld E. M. Eckard R. J. Wang M. (2020). Review: fifty years of research on rumen methanogenesis: lessons learned and future challenges for mitigation. Animals 14, S2–S16. doi: 10.1017/S1751731119003100, PMID: 32024560

[ref11] Beck M. R. Thompson L. R. Campbell T. N. Stackhouse-Lawson K. A. Archibeque S. L. (2022). Implied climate warming contributions of enteric methane emissions are dependent on the estimate source and accounting methodology. Appl. Anim. Sci. 38, 639–647. doi: 10.15232/aas.2022-02344

[ref12] Beck M. R. Thompson L. R. Rowntree J. E. Thompson T. N. Koziel J. A. Place S. E.. (2023). U.S. manure methane emissions represent a greater contribution to implied climate warming than enteric methane emissions using the global warming potential methodology. Front. Sustain. Food Sys. 7:1209541. doi: 10.3389/fsufs.2023.1209541

[ref13] Belanche A. Doreau M. Edwards J. E. Moorby J. M. Pinloche E. Newbold C. J. (2012). Shifts in the rumen microbiota due to the type of carbohydrate and level of protein ingested by dairy cattle are associated with changes in rumen fermentation. J. Nutr. 142, 1684–1692. doi: 10.3945/jn.112.159574, PMID: 22833657

[ref14] Ben-Shabat S. K. Sasson G. Doron-Faigenboim A. Durman T. Yaacoby S. Miller M. E. B.. (2016). Specific microbiome-dependent mechanisms underlie the energy harvest efficiency of ruminants. ISME J. 10, 2958–2972. doi: 10.1038/ismej.2016.62, PMID: 27152936 PMC5148187

[ref15] Benton M. J. (2009). The red queen and the court jester: species diversity and the role of biotic and abiotic factors through time. Science 323, 728–732. doi: 10.1126/science.1157719, PMID: 19197051

[ref16] Bergman E. N. (1990). Energy contributions of volatile fatty acids from the gastrointestinal tract in various species. Physiol. Rev. 70, 567–590. doi: 10.1152/physrev.1990.70.2.567, PMID: 2181501

[ref9007] Besharati M. Maggiolino A. Palangi V. Kaya A. Jabbar M. Eseceli H.. (2022). Tannin in ruminant nutrition: Review. Molecules. 27:8273. doi: 10.3390/molecules27238273 36500366 PMC9738529

[ref17] Bokulich N. A. Chung J. Battaglia T. Henderson N. Jay M. Li H.. (2016). Antibiotics, birth mode, and diet shape microbiome maturation during early life. Sci. Transl. Med. 8:343ra82. doi: 10.1126/scitranslmed.aad7121, PMID: 27306664 PMC5308924

[ref18] Bonachela J. A. Wortel M. T. Stenseth N. C. (2017). Eco-evolutionary red queen dynamics regulate biodiversity in a metabolite-driven microbial system. Nat. Sci. Rep. 7:17655. doi: 10.1038/s41598-017-17774-4, PMID: 29247226 PMC5732168

[ref19] Brockhurst M. A. Chapman T. King K. C. Mank J. E. Paterson S. Hurst G. D. D. (2014). Running with the red queen: the role of biotic conflicts in evolution. Proc. R. Soc. B 281:1382. doi: 10.1098/rspb.2014.1382 PMC4240979 25355473

[ref20] Burggraf S. Stetter K. O. Rouviere P. Woese C. R. (1991). *Methanopyrus kandleri*: an archaeal methanogen unrelated to all other methanogens. Syst. Appl. Microbiol. 14, 346–351. doi: 10.1016/S0723-2020(11)80308-5 11540073

[ref21] Carnachan S. M. Bell T. J. Hinkley S. F. Sims I. M. (2019). Polysaccharides from New Zealand native plants: a review of their structure, properties, and potential applications. Plan. Theory 8:163. doi: 10.3390/plants8060163, PMID: 31181819 PMC6630198

[ref22] Chowdhury R. Thompson L. Frazier N. A. Koziel J. A. Beck M. R. (2024). From Fermi calculations to artificial intelligence paradigms for enteric methane mitigation. Ani. Front. [Preprint]. doi: 10.1093/af/vfae025 PMC11700611 39764521

[ref23] Chu D. M. Ma J. Prince A. L. Antony K. M. Seferovic M. D. Aagaard K. M. (2017). Maturation of the infant microbiome community structure and function across multiple body sites and in relation to mode of delivery. Nat. Med. 23, 314–326. doi: 10.1038/nm.4272, PMID: 28112736 PMC5345907

[ref24] Church D. C. (1979). Digestive physiology and nutrition of ruminants. 2nd Edn. Corvallis, OR: O and B Books.

[ref25] Clemmons B. A. Martino C. Powers J. B. Campagna V. Voy B. H. Donohoe D. R.. (2019). Rumen bacteria and serum metabolites predictive of feed efficiency phenotypes in beef cattle. Sci. Rep. 9:19265. doi: 10.1038/s41598-019-55978-y, PMID: 31848455 PMC6917770

[ref26] Cohan F. M. (2006). Towards a conceptual and operational union of bacterial systemics, ecology, and evolution. Philos. Trans. R. Soc. Lond. Ser. B Biol. Sci. 361, 1985–1996. doi: 10.1098/rstb.2006.1918, PMID: 17062416 PMC1764936

[ref27] Cunningham H. C. Austin K. J. Powell S. R. Carpenter K. T. Cammack K. M. (2018). Potential response of the rumen microbiome to mode of delivery from birth through weaning. Transl. Anim. Sci. 2, S35–S38. doi: 10.1093/tas/txy029, PMID: 32704733 PMC7200978

[ref28] Custer G. F. Bresciani L. Dini-Andreote F. (2022). Ecological and evolutionary implications of microbial dispersal. Front. Microbiol. 13:855859. doi: 10.10.3389/fmicb.2022.855859 35464980 PMC9019484

[ref30] Debray R. Conover A. Zhang X. Dewald-Yang E. A. Koskella B. (2023). Within-host adaptation alters priority effects within the tomato phylosphere microbiome. Nat. Ecol. Evol. 7, 725–731. doi: 10.1038/s41559-023-02040-w, PMID: 37055621

[ref31] Debray R. Herbert R. A. Jaffe A. L. Crits-Cristoph A. Power M. E. Koskella B. (2022). Priority effects in microbiome assembly. Nat. Rev. Microbiol. 20, 109–121. doi: 10.1038/s41579-021-00604-w 34453137

[ref32] Decaestecker E. De Gersem H. Michalakis Y. Raeymaekers J. A. M. (2013). Damped long-term host-parasite red queen coevolutionary dynamics: a reflection of dilution effects? Ecol. Lett. 16, 1455–1462. doi: 10.1111/ele.12186, PMID: 24118657

[ref29] de Haas Y. Pszczola M. Soyeurt H. Wall E. Lassen J. (2017). Invited review: phenotypes to genetically reduce greenhouse gas emissions in dairying. J. Dairy Sci. 100, 855–870. doi: 10.3168/jds.2016-11246, PMID: 27939541

[ref33] Dhakal S Minx JC Toth FL Abdel-Aziz A Figueroa MJ Hubacek IBC. In IPCC, 2022: Climate change 2022: Mitigation of climate change. Contribution of working group III to the sixth assessment report of the intergovernmental panel on climate change [Shukla P. R. Skea J. Slade R. Khourdajie A. Al Diemen R. van, (eds.)]. Cambridge University Press, Cambridge, UK and New York, NY, USA. (2022).

[ref34] Diamond J. G. (1999). Germs, and steel: The fates of human societies. New York, USA: W.W. Norton.

[ref35] Edenhofer O. (2015). Climate change 2014: Mitigation of climate change, vol. 3. Cambridge, MA, USA: Cambridge university press.

[ref36] Enzmann F. Mayer F. Rother M. Holtmann D. (2018). Methanogens: biochemical background and biotechnological applications. AMB Express 8:1. doi: 10.1186/s13568-017-0531-x, PMID: 29302756 PMC5754280

[ref37] Evans S. Martiny J. B. H. Allison S. D. (2017). Effects of dispersal and selection on stochastic assembly in microbial communities. ISME J. 11, 176–185. doi: 10.1038/ismej.2016.96, PMID: 27494293 PMC5315486

[ref38] Fernando S. C. Purvis H. T. Najar F. Z. Sukharnikov L. O. Krehbiel C. R. Nagaraja T. G.. (2010). Rumen microbial population dynamics during adaptation to a high grain diet. Appl. Environ. Microbiol. 76, 7482–7490. doi: 10.1128/AEM.00388-10, PMID: 20851965 PMC2976194

[ref39] Finn D. Ouwerkerk D. Klieve A. (2012). “Methanotrophs from natural ecosystems as biocontrol agents for ruminant methane” in Final report for Meat & Livestock Australia Limited The publisher is Meat & Livestock Australia Limited.

[ref40] Flint H. J. (1997). The rumen microbial ecosystem – some recent developments. Trends Microbiol. 5, 483–488. doi: 10.1016/S0966-842X(97)01159-1, PMID: 9447660

[ref9008] Flint H. J. Scott K. P. Duncan S. H. Louis P. Forano E. (2012). Microbial degradation of complex carbohydrates in the gut. Gut Microbes. 3:289–306. doi: 10.4161/gmic.19897 22572875 PMC3463488

[ref41] Fodelianakis S. Valenzuela-Cuevas A. Barozzi A. Daffonchio D. (2021). Direct quantification of ecological drit at the population level in synthetic bacterial communities. ISME J. 15, 55–66. doi: 10.1038/s41396-020-00754-4, PMID: 32855435 PMC7852547

[ref42] Fonty G. Gouet P. Jouany J. P. Senaud J. (1987). Establishment of the microflora and anaerobic fungi in the rumen of lambs. J. Gen. Microbiol. 133, 1835–1843. doi: 10.1099/00221287-133-7-1835

[ref43] Frazier AN. (2021). Application of biotechnology in agriculture: A bioinformatic and market analysis of novel intervention methods. Doctoral dissertation. Colorado State University.

[ref44] Frazier A. N. Belk A. D. Beck M. R. Koziel J. A. (2024). Impact of methane mitigation strategies on the native ruminant microbiome: a protocol for a systematic review and meta-analysis. PLoS One 19:e0308914. doi: 10.1371/journal.pone.0308914, PMID: 39172818 PMC11340963

[ref45] Frazier A. N. Yang H. (2023). Utilizing a CRISPR-Cas9 targeted foodborne pathogen antimicrobial within livestock feed: a market research perspective. J. Agricul. Food Res. 14:100758. doi: 10.1016/j.jafr.2023.100758

[ref46] Friedman N. Shriker E. Gold B. (2017). Diet-induced changes of redox potential underlie compositional shifts in the rumen archaeal community. Environ. Microbiol. 19, 174–184. doi: 10.1111/1462-2920.13551 27696646

[ref47] Fukami T. (2015). Historical contingency in community assembly: integrating niches, species pools, and priority effects. Annu. Rev. Ecol. Evol. Syst. 46, 1–23. doi: 10.1146/annurev-ecolsys-110411-160340

[ref48] Furman O. Shenhav L. Sasson G. Kokou F. Honig H. Jacoby S.. (2020). Stochasticity constrained by deterministic effects of diet and age drive rumen microbiome assembly dynamics. Nat. Commun. 11:1904. doi: 10.1038/s41467-020-15652-8, PMID: 32312972 PMC7170844

[ref49] Gerber P. J. Steinfeld H. Henderson B. Mottet A. Opio C. Dijkman J.. (2013). Tackling climate change through livestock – A global assessment of emissions and mitigation opportunities. Rome, Italy: Food and Agriculture Organization of the United Nations (FAO).

[ref50] Glasson C. R. K. Kinley R. D. de Nys R. King N. Adams S. L. Packer M. A.. (2022). Benefits and risks of including the bromoform containing seaweed Asparagopis in feed for the reduction of methane production from ruminants. Algal Res. 64:102673. doi: 10.1016/j.algal.2022.102673

[ref51] Gribaldo S. Brochier-Armanet C. (2006). The origin and evolution of Archaea: a state of the art. Philos. Trans. R. Soc. B 361, 1007–1022. doi: 10.1098/rstb.2006.1841, PMID: 16754611 PMC1578729

[ref52] Guerrero-Cruz S. Vaksmaa A. Horn M. A. Niemann H. Pijuan M. Ho A. (2021). Methanotrophs: discoveries, environmental relevance, and a perspective on current and future applications. Front. Virol. 12:678057. doi: 10.3389/fmicb.2021.678057, PMID: 34054786 PMC8163242

[ref53] Guzman C. E. Bereza-Malcolm L. T. De Groef B. Franks A. E. (2015). Presence of selected methanogens, fibrolytic bacteria, and proteobacteria in the gastrointestinal tract of neonatal dairy calves from birth to 72 hours. PLoS One 10:e0133048. doi: 10.1371/journal.pone.0133048, PMID: 26186002 PMC4505879

[ref54] Hackmann T. J. Ngugi D. K. Firkins J. L. (2017). Genomes of rumen bacteria encode atypical pathways for fermenting hexoses to short-chain fatty acids. Environmentalist 19, 4670–4683. doi: 10.1111/1462-2920.13929, PMID: 28892251

[ref56] Hanson C. A. Fuhrman J. A. Horner-Devine M. C. Martiny J. B. H. (2012). Beyond biogeographic patterns: processes shaping the microbial landscape. Nat. Rev. Microbiol. 10, 497–506. doi: 10.1038/nrmicro2795 22580365

[ref55] Han X. Li B. Wang S. Chen Y. Yang Y. (2019). Effect of dietary concentrate to forage ratios on ruminal bacterial and anaerobic fungal populations of cashmere goats. Anaerobe 59, 118–125. doi: 10.1016/j.anaerobe.2019.06.010, PMID: 31228671

[ref9006] Hassan F. Arshad M. A. Ebeid H. M. Rehman M. S. Khan M. S. Shahid S.. (2020). Phytogenic additives can modulate rumen microbiome to mediate fermentation kinetics and methanogenesis through exploiting diet-microbe interaction. Front. Vet. Sci. 7:575801. doi: 10.3389/fvets.2020.575801 33263013 PMC7688522

[ref9005] Henderson G. Cox F. Ganesh S. Jonker A. Young W. Collaborators Global Rumen Census. (2015). Rumen microbial community composition varies with diet and host, but a core microbiome is found across a wide geographical range. Nat. Sci. Rep. 5:14567. doi: 10.1038/srep14567 PMC4598811 26449758

[ref57] Hobson P. N. Stewart C. S. (2012). The rumen microbial ecosystem. New York, USA: Blackie Academic & Professional.

[ref58] Hook S. E. Wright A.-D. G. McBride B. W. (2010). Methanogens: methane producers of the rumen and mitigation strategies. Archaea 2010:945785. doi: 10.1155/2010/945785, PMID: 21253540 PMC3021854

[ref60] Huws S. A. Creevey C. J. Oyama L. B. Mizrahi I. Denman S. E. Popova M.. (2018). Addressing global ruminant agricultural challenges through understanding the rumen microbiome: past, present and future. Front. Microbiol. 9:2161. doi: 10.3389/fmicb.2018.02161 30319557 PMC6167468

[ref59] Hu X. Li S. Mu R. Guo J. Zhao C. Cao Y.. (2022). The rumen microbiota contributes to the development of mastitis in dairy cows. Microbiol. Spect. 10, e02512–e02521. doi: 10.1128/spectrum.02512-21, PMID: 35196821 PMC8865570

[ref61] IPCC. (2021). “Climate change 2021: the physical science basis.” *Contribution of working I to the sixth assessment report of the Intergovernmental Panel on Climate Change*.

[ref62] Iqbal M. W. Zhang Q. Yang Y. Li L. Zou C. Huang C.. (2018). Comparative study of rumen fermentation and microbial community differences between water buffalo and Jersey cows under similar feeding conditions. J. Appl. Anim. Res. 46, 740–748. doi: 10.1080/09712119.2017.1394859

[ref63] Jablonski D. Sepkoski J. J. Jr. (1996). Paleobiology, community ecology, and scales of ecological pattern. Ecology 77, 1367–1378. doi: 10.2307/2265534, PMID: 11539425

[ref64] Jami E. Israel A. Kotser A. Mizrahi I. (2013). Exploring the bovine rumen bacterial community from birth to adulthood. ISME J. 7, 1069–1079. doi: 10.1038/ismej.2013.2, PMID: 23426008 PMC3660679

[ref65] Janssen P. H. (2010). Influence of hydrogen on rumen methane formation and fermentation balances through microbial growth kinetics and fermentation thermodynamics. Anim. Feed Sci. Technol. 160, 1–22. doi: 10.1016/j.anifeedsci.2010.07.002

[ref66] Janssen P. H. Kirs M. (2008). Structure of the archaeal community of the rumen. Appl. Environ. Microbiol. 74, 3619–3625. doi: 10.1128/AEM.02812-07 18424540 PMC2446570

[ref67] Jeyanathan J. Martin C. Morgavi D. P. (2014). The use of direct-fed microbials for mitigation of ruminant methane emissions: a review. Animal 8, 250–261. doi: 10.1017/S1751731113002085, PMID: 24274095

[ref9004] Jones M. W. Peters G. P. Gasser T. Andrew R. M. Schwingshackl C. Gütschow J.. (2024). National contributions to climate change due to historical emissions of carbon dioxide, methane, and nitrous oxide. Scientific Data. Zenodo. doi: 10.5281/zenodo.10839859 PMC10060593 36991071

[ref68] Khibnik A. I. Kondrashove A. S. (1997). Three mechanisms of red queen dynamics. Proc. R. Soc. Lond. B 264, 1049–1056. doi: 10.1098/rspb.1997.0145

[ref69] Kim M. Morrison M. Yu Z. (2011). Status of the phylogenetic diversity census of ruminal microbiomes. FEMS Microbiol. Ecol. 76, 49–63. doi: 10.1111/j.1574-6941.2010.01029.x, PMID: 21223325

[ref70] Koeppel A. Perry E. B. Sikorski J. Krizanc D. Warner A. Ward D. M.. (2008). Identifying the fundamental units of bacterial diversity: a paradigm shift to incorporate ecology into bacterial systematics. Proc. Nati. Acad. Sci. U.S.A. 105, 2504–2509. doi: 10.1073/pnas.0712205105, PMID: 18272490 PMC2268166

[ref71] Koskella B. Hall L. J. Metcalf J. E. (2017). The microbiome beyond the horizon of ecological and evolutionary theory. Nat. Ecol. Evol. 1, 1606–1615. doi: 10.1038/s41559-017-0340-2, PMID: 29038487

[ref72] Kozlowska M. Cieślak A. Jóźwik A. El-Sherbiny M. Stochmal A. Oleszek W.. (2020). The effect of total and individual alfalfa saponins on rumen methane production. J. Sci. Food Agric. 100, 1922–1930. doi: 10.1002/jsfa.10204 31846083

[ref73] Leibold M. A. Holyoak M. Mouquet N. Amarasekare P. Chase J. M. Hoopes M. F.. (2004). The metacommunity concept: a framework for multi-scale community ecology. Ecol. Lett. 7, 601–613. doi: 10.1111/j.1461-0248.2004.00608.x

[ref74] Leng R. A. (2014). Interactions between microbial consortia in biofilms: a paradigm shift in rumen microbial ecology and enteric methane mitigation. Anim. Prod. Sci. 54, 519–543. doi: 10.1071/AN13381

[ref76] Li F. Li C. Chen Y. Liu J. Zhang C. Irving B.. (2019). Host genetics influence the rumen microbiota and heritable rumen microbial features associate with feed efficiency in cattle. Microbiome. 7:92. doi: 10.1186/s40168-019-0699-1, PMID: 31196178 PMC6567441

[ref75] Li R. W. Connor E. E. Li C. Baldwin R. L. Sparks M. E. (2012). Characterization of the rumen microbiota of pre-ruminant calves using metagenomic tools. Environ. Microbiol. 14, 129–139. doi: 10.1111/j.1462-2920.2011.02543.x 21906219

[ref77] Li Y. Q. Xi Y. M. Wang Z. D. Zeng H. F. Han Z. (2020). Combined signature of rumen microbiome and metabolome in dairy cows with different feed intake levels. J. Anim. Sci. 98, 1–15. doi: 10.1093/jas/skaa070, PMID: 32141506 PMC7098705

[ref78] Lowe W. H. McPeek M. A. (2014). Is dispersal neutral? Trends Ecol. Evol. 29, 444–450. doi: 10.1016/j.tree.2014.05.009 24962790

[ref79] Luckey T. D. (2012). Germfree life and Gnotobiology. London, United Kingdom: Academic Press Inc., 402–418.

[ref80] Maasakkers J. D. Jacob D. J. Sulprizio M. P. Scarpelli T. R. Nesser H. Sheng J.-X.. (2019). Global distribution of methane emissions, emission trends, and OH concentrations and trends inferred from an inversion of GOSAT satellite data for 2010-2015. Atmos. Chem. Phys. 19, 7859–7881. doi: 10.5194/acp-19-7859-2019

[ref81] Maman L. G. Palizban F. Atanaki F. F. Ghiasi N. E. Ariaeenejad S. Ghaffari M. R.. (2020). Co-abundance analysis reveals hidden players associated with high methane yield phenotype in sheep rumen microbiome. Nat. Sci. Rep. 10:4995. doi: 10.1038/s41598-020-61942-y, PMID: 32193482 PMC7081230

[ref82] Martinez-Álvaro M. Auffret M. D. Stewart R. D. Dewhurst R. J. Duthie C.-A. Rooke J. A.. (2020). Identification of complex rumen microbiome interaction within diverse functional niches as mechanisms affecting the variation of methane emissions in bovine. Front. Microbiol. 11:659. doi: 10.3389/fmicb.2020.00659, PMID: 32362882 PMC7181398

[ref83] Matte E. Forsberg C. W. Verrinder Gibbins A. M. (1992). Enzymes associated with metabolism of xylose and other pentoses by Prevotella (Bacteroides) ruminicola strains, *Selenomonas ruminantium* D, and *Fibrobacter succinogenes* S85. Can. J. Microbiol. 38, 370–S376. doi: 10.1139/m92-063, PMID: 1643581

[ref84] McGill B. J. Enquist B. J. Weiher E. Westoby M. (2006). Rebuilding community ecology from functional traits. Trends Ecol. Evol. 21, 178–185. doi: 10.1016/j.tree.2006.02.002, PMID: 16701083

[ref85] McGovern E. McGee M. Byrne C. J. Kenny D. A. Kelly A. K. Waters S. M. (2020). Investigation into the effect of divergent feed efficiency phenotype on the bovine rumen microbiota across diet and breed. Sci. Rep. 10:15317. doi: 10.1038/s41598-020-71458-0, PMID: 32948787 PMC7501277

[ref87] Meale S. J. Li S. Azevedo P. Derakhshani H. Plaizier J. C. Khafipour E.. (2016). Development of ruminal and fecal microbiomes are affected by weaning but not weaning strategy in dairy calves. Front. Microbiol. 7:582. doi: 10.3389/fmicb.2016.00582 27199916 PMC4853645

[ref86] Meale S. J. Li S. C. Azevedo P. Derakhshani H. DeVries T. J. Plaizier J. C.. (2017). Weaning age influences the severity of gastrointestinal microbiome shifts in dairy calves. Nat. Sci. Rep. 7:198. doi: 10.1038/s41598-017-00223-7, PMID: 28298634 PMC5428063

[ref88] Meale S. J. Popova M. Saro C. Martin C. Bernard A. Lagree M.. (2021). Early life dietary intervention in dairy calves results in a long-term reduction in methane emissions. Sci. Rep. Nat. 11:3003. doi: 10.1038/s41598-021-82084-9, PMID: 33542279 PMC7862406

[ref89] Mills L. S. Soulé M. E. Doak D. F. (1993). The keystone-species concept in ecology and conservation. Bioscience 43, 219–224. doi: 10.2307/1312122

[ref90] Min B. R. Parker D. Brauer D. Waldrip H. Lockard C. Hales K.. (2021). The role of seaweed as a potential dietary supplementation for enteric methane mitigation in ruminants: challenges and opportunities. Anim. Nutr. 7, 1371–1387. doi: 10.1016/j.aninu.2021.10.003, PMID: 34786510 PMC8581222

[ref91] Mitsumori M. Ajisaka N. Tajima K. Kajikawa H. Kurihara M. (2002). Detection of *Proteobacteria* from the rumen by PCR using methanotroph-specific primers. Lett. Appl. Microbiol. 35, 251–255. doi: 10.1046/j.1472-765X.2002.01172.x 12180951

[ref92] Mizrahi I. (2013). “Rumen Symbioses” in The prokaryotes. eds. Rosenberg E. DeLong E. F. Lory S. Stackebrandt E. Thompson F. (Berlin Heidelberg: Springer-Verlag), 533–544.

[ref93] Mizrahi I. Jami E. (2021). A method to the madness: disentangling the individual forces that shape the rumen microbiome. European Molecular Biol. Organization reports 22:e52269. doi: 10.15252/embr.202052269, PMID: 33528098 PMC7857420

[ref94] Mizrahi I. Wallace R. J. Moraïs S. (2021). The rumen microbiome: balancing food security and environmental impacts. Natl. Rev. 19, 553–566. doi: 10.1038/s41579-021-00543-6, PMID: 33981031

[ref95] Moraïs S. Mizrahi I. (2019). The road not taken: the rumen microbiome, functional groups, and community states. Trends Microbiol. 27, 538–549. doi: 10.1016/j.tim.2018.12.011 30679075

[ref9003] Morris J. J. Lenski R. E. Zinser E. R. (2012). The Black Queen Hypothesis: evolution of dependencies through adaptive gene loss. mBio. 3:e00036–12. doi: 10.1128/mbio.00036-12 22448042 PMC3315703

[ref96] Morvan B. Doré J. Rieu-Lesme F. Foucat L. Fonty G. Gouet P. (1994). Establishment of hydrogen-utilizing bacteria in the rumen of the newborn lamb. FEMS Microbiol. Lett. 117, 249–256. doi: 10.1111/j.1574-6968.1994.tb06775.x, PMID: 8200502

[ref97] Nemergut D. R. Schmidt S. K. Fukami T. O’Neill S. P. Bilinski T. M. Stanish L. F.. (2013). Patterns and processes of microbial community assembly. Microbiol Mol. Biol. Res. 77, 342–356. doi: 10.1128/MMBR.00051-12, PMID: 24006468 PMC3811611

[ref98] Newbold C. J. Ramos-Morales E. (2020). Review: ruminal microbiome and microbial metabolome: effects of diet and ruminant host. Animal 14, s78–s86. doi: 10.1017/S1751731119003252 32024572

[ref99] O’Hara E. Kenny D. A. McGovern E. Byrne C. J. McCabe M. S. Guan L. L.. (2020). Investigating the temporal microbial dynamics in the rumen of beef calves raised on two farms during early life. FEMS Mirobiol. Ecol. 96:fiz203. doi: 10.1093/femsec/fiz203, PMID: 31917419

[ref100] Ocko I. B. Sun T. Shindell D. Oppenheimer M. Hristov A. N. Pacala S. W.. (2021). Acting rapidly to deploy readily available methane mitigation measures by sector can immediately slow global warming. Environ. Res. Lett. 16:054042. doi: 10.1088/1748-9326/abf9c8

[ref101] OECD/FAO (2020). OECD-FAO agricultural outlook 2020-2029. United Nations: OECD publishing/food and agriculture Organization.

[ref102] Paine R. T. (1966). Food web complexity and species diversity. Am. Nat. 100, 65–75. doi: 10.1086/282400

[ref103] Pan Z. Ma T. Steele M. Guan L. L. (2024). Varied microbial community assembly and specialization patterns driven by early life microbiome perturbation and modulation in young ruminants. ISME Com. 4:ycae044. doi: 10.1093/ismeco/ycae044, PMID: 38650709 PMC11033733

[ref104] Pereira A. M. de Lurdes Nunes Enes Dapkevicius M. Borba A. E. S. (2022). Alternative pathways for hydrogen sink originated from the ruminal fermentation of carbohydrates: which microorganisms are involved in lowering methane emission? Anim. Microbiome. 4:5. doi: 10.1186/s42523-021-00153-w, PMID: 34991722 PMC8734291

[ref105] Pitta D. W. Indugu N. Melgar A. Hristov A. Challa K. Vecchiarelli B.. (2022). The effect of 3-nitrooxypropanol, a potent methane inhibitor, on ruminal microbial gene expression profiles in dairy cows. Microbiome. 10:146. doi: 10.1186/s40168-022-01341-9, PMID: 36100950 PMC9469553

[ref106] Prosser J. I. Bohannan B. J. M. Curtis T. P. Ellis R. J. Firestone M. K. Freckleton R. P.. (2007). The role of ecological theory in microbial ecology. Nat. Rev. Microbiol. 5, 384–392. doi: 10.1038/nrmicro1643 17435792

[ref107] Rabajante J. F. Tubay J. M. Uehara T. Morita S. Ebert D. Yoshimura J. (2015). Red queen dynamics in multi-host and multi-parasite interaction system. Sci. Rep. 5:10004. doi: 10.1038/srep10004 25899168 PMC4405699

[ref108] Rani A. Pundir A. Verma M. Joshi S. Verma G. Andjelković S.. (2024). Methanotrophy: a biological method to mitigate global methane emission. Microbiol. Res. 15, 634–654. doi: 10.3390/microbiolres15020042

[ref109] Rey M. Enjalbert F. Combes S. Cauquil L. Bouchez O. Monteil V. (2013). Establishment of ruminal bacterial community in dairy calves from birth to weaning is sequential. J. Appl. Microbiol. 116, 245–257. doi: 10.1111/jam.12405, PMID: 24279326

[ref110] Roehe R. Dewhurst R. J. Duthie C. A. Rooke J. A. McKain N. Ross D. W.. (2016). Bovine host genetic variation influences rumen microbial methane production with best selection criterion for low methane emitting and efficiently feed converting hosts based on metagenomic gene abundance. PLoS Genet. 12:e1005846. doi: 10.1371/journal.pgen.1005846, PMID: 26891056 PMC4758630

[ref112] Roque B. M. Salwen J. K. Kinley R. Kebreab E. (2019). Inclusion of *Asparagopsis armata* in lactating dairy cows diet reduces enteric methane emission by over 50 percent. J. Clean. Prod. 234, 132–138. doi: 10.1016/j.jclepro.2019.06.193

[ref111] Roque B. R. Brooke C. G. Ladau J. Polley T. Marsh L. J. Najafi N.. (2019). Effect of the macroalgae *Asparagopsis taxiformis* on methane production and rumen microbiome assemblage. Ani. Microbiome. 1:3. doi: 10.1186/s42523-019-0004-4, PMID: 33499933 PMC7803124

[ref113] Roughgarden J. Gilbert S. F. Rosenberg E. Zilber-Rosenberg I. Lloyd E. A. (2017). Holobionts as units of selection and a model of their population dynamics and evolution. Biol. Theory 13, 44–65. doi: 10.1007/s13752-017-0287-1

[ref114] Russell J. B. (1998). The importance of pH in the regulation of ruminal acetate to propionate ratio and methane production in vitro. J. Dairy Sci. 81, 3222–3230. doi: 10.3168/jds.S0022-0302(98)75886-2 9891267

[ref115] Sarkar A. Harty S. Johnson K. V.-A. Moeller A. H. Archie A. E. Schell L. D.. (2020). Microbial transmission in animal social networks and the social microbiome. Nat. Ecol. Evol. 4, 1020–1035. doi: 10.1038/s41559-020-1220-8, PMID: 32572221

[ref116] Schilde M. von Soosten D. Hüther L. Meyer U. Zeyner A. Dänicke S. (2021). Effects of 3-nitrooxypropanol and varying concentrate feed proportions in the ration on methane emission, rumen fermentation and performance of periparturient dairy cows. Arch. Anim. Nutr. 75, 79–104. doi: 10.1080/1745039X.2021.1877986, PMID: 33641544

[ref117] Shaani Y. Zehavi T. Eyal S. Miron J. Mizrahi I. (2018). Microbiome niche modification drives diurnal rumen community assembly, overpowering individual variability and diet effects. ISME J. 12, 2446–2457. doi: 10.1038/s41396-018-0203-0, PMID: 29921849 PMC6154959

[ref118] Shade A. Jones S. E. Caporaso J. G. Handelsman J. Knight R. Fierer N.. (2014). Conditionally rare taxa disproportionately contribute to temporal changes in microbial diversity. MBio 5:4. doi: 10.1128/mbio.01371-14 PMC4161262 25028427

[ref119] Smillie C. S. Smith M. B. Friedman J. Cordero O. X. David L. A. Alm E. J. (2011). Ecology drives a global network of gene exchange connecting the human microbiome. Nature 480, 241–244. doi: 10.1038/nature10571, PMID: 22037308

[ref120] Snelling T. J. Auffret M. D. Duthie C.-A. Stewart R. D. Watson M. Dewhurst R. J.. (2019). Temporal stability of the rumen microbiota in beef cattle, and response to diet and supplements. Anim. Microbiome. 1:16. doi: 10.1186/s42523-019-0018-y, PMID: 33499961 PMC7807515

[ref121] Söllinger A. Tveit A. T. Poulsen M. Noel S. J. Bengtsson M. Bernhardt J.. (2018). Holistic assessment of rumen microbiome dynamics through quantitative metatranscriptomics reveals multifunctional redundancy during key steps of anaerobic feed digestion. mSystems. 3, e00038–e00018. doi: 10.1128/mSystems.00038-18, PMID: 30116788 PMC6081794

[ref122] Song H.-S. Renslow R. S. Fredrickson J. K. Lindemann S. R. (2015). Integrating ecological and engineering concepts of resilience in microbial communities. Front. Microbiol. 6:1298. doi: 10.3389/fmicb.2015.01298, PMID: 26648912 PMC4664643

[ref123] Soren N. M. Malik P. K. Sejian V. (2015). “Methanotrophs in enteric methane mitigation” in Livestock production and climate change, no. 6. Eds. Malik P. K. Takahashi B. J. Kohn R. A. Prasad C. S. (CABI Digital Library). 360–375.

[ref124] Stein R. R. Bucci V. Toussaint N. C. Buffie C. G. Rätsch G. Pamer E. G.. (2013). Ecological modeling from time-series inference: insight into dynamics and stability of intestinal microbiota. PLoS Comput. Biol. 9:e1003388. doi: 10.1371/journal.pcbi.1003388, PMID: 24348232 PMC3861043

[ref125] Stewart C. J. Embleton N. D. Clements E. Luna P. N. Smith D. P. Fofanova T. Y.. (2017). Cesarean or vaginal birth does not impact longitudinal development of the gut microbiome in a cohort of exclusively preterm infants. Front. Microbiol. 8:1008. doi: 10.3389/fmicb.2017.01008 28634475 PMC5459931

[ref126] Storm E. Ørskov E. (1983). The nutritive value of rumen microorganisms in ruminants: 1. Large-scale isolation and chemical composition of rumen microorganisms. Br. J. Nutr. 50, 463–470. doi: 10.1079/BJN19830114, PMID: 6615774

[ref127] Strotz L. C. Simões M. Girard M. G. Breitkreuz L. Kimmig U. J. Lieberman B. S. (2018). Getting somewhere with the red queen: chasing a biologically modern definition of the hypothesis. Biol. Lett. 14:20170734. doi: 10.1098/rsbl.2017.0734, PMID: 29720444 PMC6012711

[ref9002] Sun Y. Allen M. S. Lock A. L. (2019). Culture pH interacts with corn oil concentration to affect biohydrogenation of unsaturated fatty acids and disappearance of neutral detergent fiber in batch culture. J. Dairy Sci. 102:9870–9882. doi: 10.3168/jds.2019-16581 31447159

[ref128] Tapio I. Snelling T. J. Strozzi F. Wallace R. J. (2017). The ruminal microbiome associated with methane emissions from ruminant livestock. J. Anim. Sci. Biotechnol. 8:7. doi: 10.1186/s40104-017-0141-0, PMID: 28123698 PMC5244708

[ref129] Taxis T. M. Wolff S. Gregg S. J. Minton N. O. Zhang C. Dai J.. (2015). The players may change but the game remains: network analyses of ruminal microbiomes suggest taxonomic differences mask functional similarity. Nucleic Acids Res. 43, gkv973–gkv9612. doi: 10.1093/nar/gkv973, PMID: 26420832 PMC4787786

[ref130] Thompson L. R. Beck M. R. Gunter S. A. Williams G. D. Place S. E. Reuter R. R. (2019). An energy supplement with monensin reduces methane emission intensity of stocker cattle grazing winter wheat. Appl. Anim. Sci. 35, 433–440. doi: 10.15232/aas.2018-01841

[ref131] Tseten T. Sanjorjo R. A. Kwon M. Kim S.-W. (2022). Strategies to mitigate enteric methane emissions from ruminant animals. J. Microbiol. Biotechnol. 32, 269–277. doi: 10.4014/jmb.2202.02019, PMID: 35283433 PMC9628856

[ref132] Ungerfeld E. M. (2020). Metabolic hydrogen flows in rumen fermentation: principles and possibilities of interventions. Front. Microbiol. 11:589. doi: 10.3389/fmicb.2020.00589, PMID: 32351469 PMC7174568

[ref133] United Nations. (2023). Global Issues: Population. https://www.un.org/en/global-issues/population (Accessed August 2, 2023)

[ref134] United Nations Environment Program and Climate and Clean Air Coalition. (2021). Global methane assessment: benefits and costs of mitigating methane emissions. United Nations Environment Program, Nairobi. Available at: https://www.unep.org/resources/report/global-methane-assessment-benefits-and-costs-mitigating-methane-emissions (Accessed August 2, 2023)

[ref135] Vallès Y. Artacho A. Pascual-García A. Ferrús M. L. Gosalbes M. J. Abellán J. J.. (2014). Microbial succession in the gut: directional trends of taxonomic and functional change in a birth cohort of Spanish infants. PLoS Genet. 10:e1004406. doi: 10.1371/journal.pgen.1004406, PMID: 24901968 PMC4046925

[ref136] Van Valen L. (1973). A new evolutionary law. Evol. Theory 1, 1–30. doi: 10.7208/9780226115504-022

[ref137] Vellend M. Srivastava D. S. Anderson K. M. Brown C. D. Jankowski J. E. Kleynhans E. J.. (2014). Assessing the relative importance of neutral stochasticity in ecological communities. Oikos 123, 1420–1430. doi: 10.1111/oik.01493

[ref138] Vollset S. E. Goren E. Yuan C.-W. Smith A. E. Hsiao T. Bisignano C.. (2020). Fertility, mortality, migration, and population scenarios for 195 countries and territories from 2017 to 2100: a forecasting analysis for the global burden of disease study. Lancet 396, 1285–1306. doi: 10.1016/S0140-6736(20)30677-2, PMID: 32679112 PMC7561721

[ref139] Wallace R. J. Sasson G. Garnsworthy P. C. Tapio I. Gregson E. Bani P.. (2019). A heritable subset of the core rumen microbiome dictates dairy cow productivity and emissions. Sci. Adv. 5:eaav8391. doi: 10.1126/sciadv.aav8391, PMID: 31281883 PMC6609165

[ref140] Wang L. Li Y. Zhang Y. Wang L. (2020). The effects of different concentrate-to-forage ratio diets on rumen bacterial microbiota and the structures of Holstein cows during the feeding cycle. Animals 10:957. doi: 10.3390/ani10060957 32486436 PMC7341334

[ref141] Weimer P. J. (2015). Redundancy, resilience, and host specificity of the ruminal microbiota: implications for engineering improved ruminal fermentations. Front. Microbiol. 6:296. doi: 10.3389/fmicb.2015.00296, PMID: 25914693 PMC4392294

[ref142] Weimer P. J. Cox M. S. Vieira de Paula T. Lin M. Hall M. B. Suen G. (2017). Transient changes in milk production efficiency and bacterial community composition resulting from near-total exchange of ruminal contents between high- and low-efficiency Holstein cows. J. Dairy Sci. 100, 7165–7182. doi: 10.3168/jds.2017-12746, PMID: 28690067

[ref143] Whiteson K. L. Bailey B. Bergkessel M. Conrad D. Delhaes L. Felts B.. (2014). The upper respiratory tract as a microbial source for pulmonary infections in cystic fibrosis. Am. J. Respir. Crit. Care Med. 189, 1309–1315. doi: 10.1164/rccm.201312-2129PP, PMID: 24702670 PMC4098084

[ref144] Whittaker R. H. (1972). Evolution and measurement of species diversity. Taxon 21, 213–251. doi: 10.2307/1218190

[ref145] Xiang R. McNally J. Rowe S. Jonker A. Pinares-Patino C. S. Oddy V. H.. (2016). Gene network analysis identifies rumen epithelial cell proliferation, differentiation and metabolic pathways perturbed by diet and correlated with methane production. Nat. Sci. Rep. 6:39022. doi: 10.1038/srep39022 PMC5155297 27966600

[ref146] Yáñez-Ruiz D. R. Abecia L. Newbold C. J. (2015). Manipulating rumen microbiome and fermentation through interventions during early life: a review. Front. Microbiol. 6:1133. doi: 10.3389/fmicb.2015.01133, PMID: 26528276 PMC4604304

[ref147] Yassour M. Vatanen T. Siljander H. Hämäläinen A.-M. Härkönen T. Ryhänen S. J.. (2016). Natural history of the infant gut microbiome and impact of antibiotic treatment on bacterial strain diversity and stability. Sci. Transl. Med. 8:343ra81. doi: 10.1126/scitranslmed.aad0917, PMID: 27306663 PMC5032909

[ref148] Yu G. Beauchemin K. A. Dong R. (2021). A review of 3-nitrooxypropanol for enteric methane mitigation from ruminant livestock. Animals 11:3540. doi: 10.3390/ani11123540, PMID: 34944313 PMC8697901

[ref9001] Yurkovetskiy L. Burrows M. Khan A. A. Graham L. Volchkov P. Becker L.. (2013). Gender bias in autoimmunity is influenced by microbiota. Immunity. 39:400–412. doi: 10.1016/j.immuni.2013.08.013 23973225 PMC3822899

[ref149] Zanevald J. R. McMinds R. Thurber R. V. (2017). Stress and stability: applying the Anna Karenina principle to animal microbiomes. Nat. Microbiol. 2:17121. doi: 10.1038/nmicrobiol.2017.121 28836573

[ref150] Zhou J. Ning D. (2017). Stochastic community assembly: does it matter in microbial ecology? Microbiol. Mol. Biol. Rev. 81, e00002–e00017. doi: 10.1128/mmbr.00002-17 29021219 PMC5706748

[ref151] Zilber-Rosenberg I. Rosenberg E. (2008). Role of microorganisms in the evolution of animals and plants: the hologenome theory of evolution. FEMS Microbiol. Rev. 32, 723–735. doi: 10.1111/j.1574-6976.2008.00123.x, PMID: 18549407

