# Supplementary figures and images for: Connecting the ruminant microbiome to climate change: insights from current ecological and evolutionary concepts
Source: Front Microbiol. 2024 Dec 2;15:1503315. doi: 10.3389/fmicb.2024.1503315 (PMC11646987; doi:10.3389/fmicb.2024.1503315)

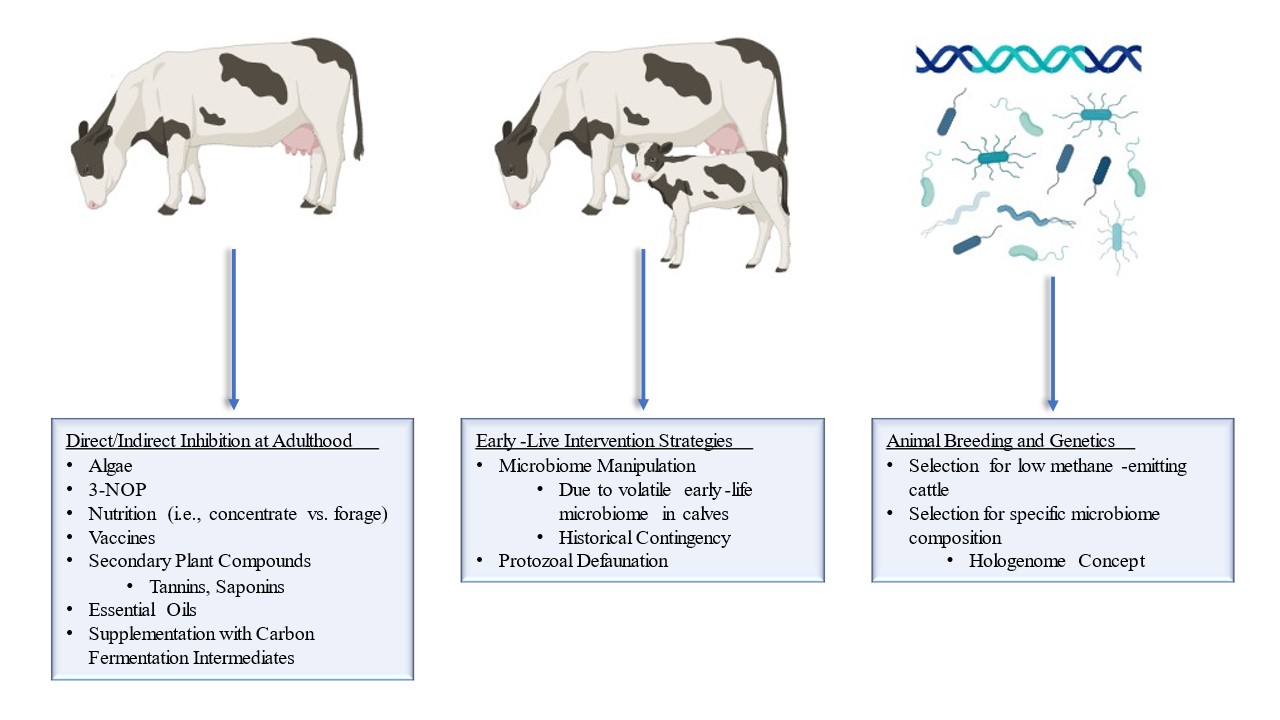

Supplement: Supplementary file 1 [file Image_1.JPEG]

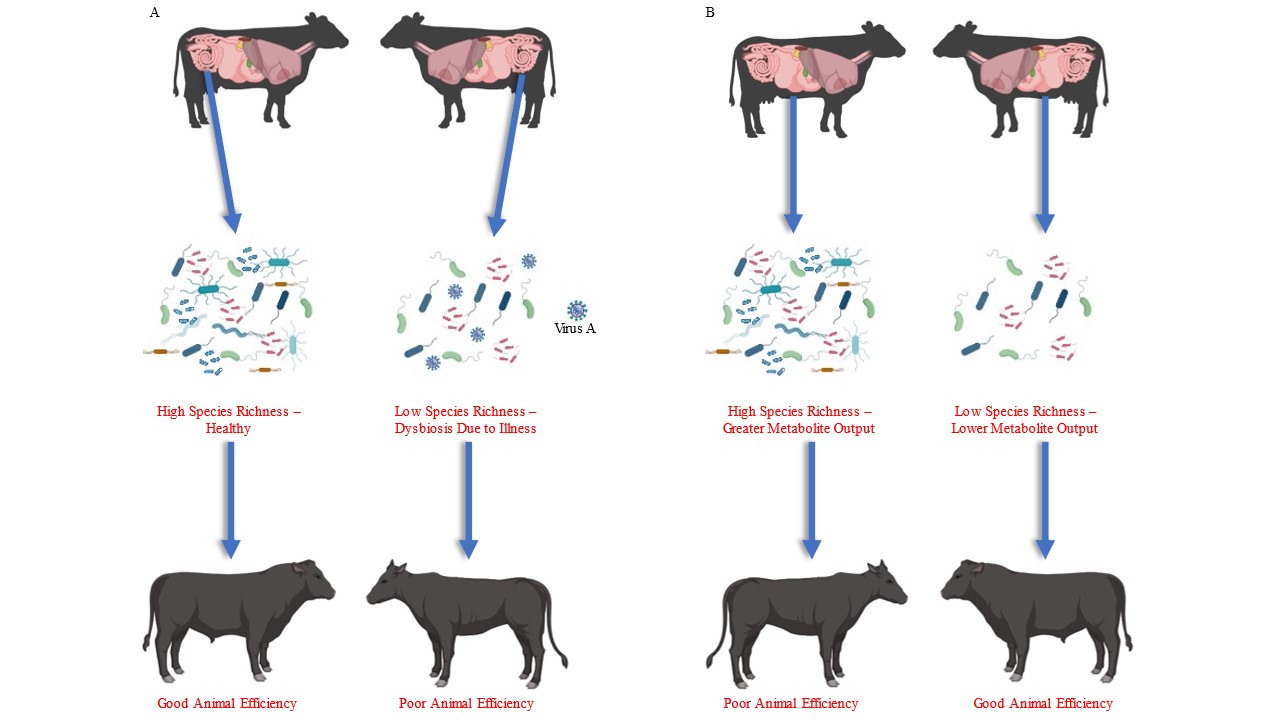

Supplement: Supplementary file 2 [file Image_2.JPEG]
