# Supplementary material for: Connecting the ruminant microbiome to climate change: insights from current ecological and evolutionary concepts
Source: Front Microbiol. 2024 Dec 2;15:1503315. doi: 10.3389/fmicb.2024.1503315 (PMC11646987; doi:10.3389/fmicb.2024.1503315)
Supplement: Supplementary file 3 [file Data_Sheet_1.DOCX]

Connecting the ruminant microbiome to climate change: Insights from current ecological and evolutionary concepts

**A. Nathan Frazier^1*^, Matthew R. Beck^1,2^, Heidi Waldrip^1^, Jacek A. Koziel^1^**

^1^United States Department of Agriculture – Agricultural Research Service, Conservation and Production Research Laboratory, Bushland, TX, 79012, USA.
^2^Department of Animal Science – Texas A&M University, College Station, TX, 77843, USA

*** Correspondence:**Corresponding Author
[anthony.frazier@usda.gov](mailto:anthony.frazier@usda.gov)

Keywords: Greenhouse Gases, Enteric Methane, Methanogenesis, Cattle, Inhibition, Rumen Microbiome, Evolution, Ecology

Supplementary Material

1. **Ruminant Microbiome Ecology**

The prokaryotic members, bacteria and archaea, are of great importance in ruminant viability and enteric CH_4_ emissions (Mizrahi, 2013). Both bacteria and archaea play integral roles in the enteric fermentation process. Bacteria form the most numerous and diverse utilizers of fiber, starch, protein, and sugar within the rumen (Stewart *et al*., 1997). At the phylum level, *Firmicutes*, *Bacteroidetes, and Proteobacteria* are the most numerous across several studies (Jami and Mizrahi, 2012; Henderson *et al*., 2015; Snelling and Wallace, 2017; Hart *et al*., 2018). Consistent with these findings, a recent study in beef cattle found the three most dominant phyla were *Firmicutes*, *Bacteroidetes*, and *Proteobacteria* with a relative abundance of 44.05%, 36.42%, and 4.61% respectively (Li *et al*., 2019). Members of the *Firmicutes* and *Bacteroidetes*, such as *Fibrobacter succinogenes*, *Ruminococcus flavefaciens*, and *Ruminococcus* *albus*, have been described as key cellulose degraders; additionally, species from the genera *Prevotella, Butyrivibrio* and *Pseudobutyrivibrio* have also been identified as key hemicellulose degraders (Mizrahi, 2013; Moraïs and Mizrahi, 2019b; Mizrahi *et al*., 2021). At the genus level, a “core ruminant bacterial microbiome” was identified by Henderson *et al*. (2015) who established that 67.1% of all bacterial sequence data from a wide range of ruminant species belonged to *Prevotella*, *Butyrivibrio*, *Ruminococcus*, unclassified *Lachnospiraceae*, unclassified *Ruminococcaceae*, unclassified *Bacteroidales*, and unclassified *Clostridiales*. This finding has been further corroborated by Wallace *et al*., (2019) who identified a core rumen microbiome in dairy cattle that could predict host phenotype. The core microbiome offers a unique opportunity for microbiome engineering research.

### 1.1 Bacteria

Members of the core bacterial microbiome are heavily involved in the ruminant fermentation process as either fibrolytic or cellulolytic species (Mizrahi, 2013; Moraïs and Mizrahi, 2019b; Mizrahi *et al*., 2021). Fibrolytic bacteria produce H_2_ during the fermentation process along with VFA and CO_2_. Cellulolytic species, such as those belonging to Bacteroidetes, are net H_2_ utilizers (Stewart *et al*., 1997). Hydrogen is the primary end-product for fibrolytic microorganisms which can result in H_2_ accumulation. Accumulation of H_2_ within the rumen headspace has been shown to have detrimental effects on the fermentation process by inhibiting certain microbial dehydrogenases (Janssen, 2010; Leng, 2014). Studies have demonstrated a link between bacterial abundances and CH_4_ emissions. For example, Wallace *et al*. (2015) found that Succinivibrionaceae (88% of Proteobacteria reads) was found in 97% in low CH_4_ emitting cattle and lower in high CH_4_ emitters (79%). Consistent with other studies (Pope *et al*., 2011; Kittelmann *et al*., 2014), low CH_4_ emitters compositionally shift towards higher abundances of H_2_-utilizing species and lower abundances of H_2_-producing species. As a result, enteric CH_4_ emissions will theoretically be lower in ruminants that have a lower abundance of H_2_-producing bacteria, such as *Ruminococcus* and *Eubacterium spp*., and higher abundances of H_2_-consuming bacteria, such as *Prevotella* and *Selenomonas spp.* (Denman *et al*., 2015; Tapio *et al*., 2017). Kittelmann *et al*. (2014) further demonstrated that low CH_4_-emitting sheep had higher abundances of propionate, lactate, and succinate producers such as *Quinell ovalis*, *Fibrobacter spp.*, *Kandleria vitulina*, *Olsenella spp*., *Prevotella bryantii*, and *Sharpea azabuensis*. Surprisingly, the genus *Desulfovibrio* – a known H_2_ utilizer – was found in significantly higher abundances in high CH_4_-emitting cattle leading to a possibility of being associated with greater H_2_ availability (Wallace *et al*., 2015). This finding, while in contradiction with other studies, could prove valuable in isolating *Delsulfovibrio* as a potential biomarker in determining H_2_ differences between low and high CH_4_ emitting ruminants. Ruminant bacterial composition and species abundance play a key role in understanding both ecological and CH_4_-emission dynamics. Therefore, it is necessary to elucidate how bacterial composition and diversity could be utilized in reducing CH_4_ emissions.

**1.2 Archaea**

Archaeal methanogens are solely responsible for the methanogenesis pathway, the primary metabolic process of removing H_2_ from the rumen produced by bacteria, protozoa, and fungi, which is then released from the host as CH_4_. Archaea represent a much smaller proportion of the total rumen microbial richness. The archaeal domain is home to the archaeal phylum of Euryarchaeota, which comprises seven orders, including the common ruminant community members: Methanobacteriales, Methanomicrobiales, Methanomassiliicoccales, and Methanosarcinales. Henderson *et al*., (2015) determined that nearly all archaeal members were methanogens with 74% of all archaea belonging to *Methanobrevibacter gottschalkii* and *Methanobrevibacter ruminantium* clades. This finding is in line with other studies as the genus *Methanobrevibacter* of the order Methanobacteriales accounted for 70% of the rumen archaeal community found in Holstein dairy cows (Friedman *et al*., 2017). While less diverse than bacteria (Henderson *et al*., 2015), evidence suggests that methanogens are highly conserved between ruminant animals (Wallace *et al*., 2019). Therefore, methanogens are considered to be part of the “core ruminant microbiome”.

Within the rumen, methanogens play a key role by acting as H_2_ sinks with CH_4_ production representing the largest H_2_ sink in the ruminant digestive tract (Beauchemin *et al*., 2020). The methanogenesis pathway occurs at the end of the ruminant electron flow and is activated by three different methanogenic functional groups (**Box 1**), all of which use differing input metabolites. These functional groups come from phylogenetic groups that span across different orders all belonging Euryarchaeota (Friedman *et al*., 2017). Methanogen functional groups include the hydrogenotrophs (Demirel and Scherer, 2008), methylotrophs (Mizrahi *et al*., 2021), and acetoclastic methanogens (Ferry, 1999; Morgavi *et al*., 2010). Hydrogenotrophs constitute most of the methanogens in mature animals and utilizes metabolic hydrogen ([H+]) to reduce CO_2_, CO, or formate to CH_4_ (Furman *et al*., 2020). Hydrogenotrophic methanogenesis is thermodynamically favorable to the other two pathways which results in lower abundances of other methanogenic organisms (Fenchel *et al*., 2012; Pereira *et al*., 2022). This understanding is consistent with *Methanobrevibacter* being the most dominant methanogenic genera. Methylotrophic methanogenesis produces CH_4_ using methylated substrates such as trimethylamine, dimethyl sulfate, and methanol (Lyu *et al*., 108). Methylotrophs represent the most abundant methanogens in newborn calves and utilize methylated compounds such as methanol or methyl amines to produce CH_4_ (Furman *et al*., 2020). It has been documented that the methylotrophic *Methanosphaera* was negatively correlated with CH_4_ emissions in both beef and dairy cattle (Cunha *et al*., 2017; Cunha *et al*., 2018; Ramayo-Caldas *et al*., 2020; Pereira *et al*., 2022). These findings could be explained by the variation of the stoichiometric properties between the hydrogenotrophic and methylotrophic pathways, which results in hydrogenotrophic methanogenesis being energetically favored (Cunha *et al*., 2017; Pereira *et al*., 2022). Finally, acetoclastic methanogens utilize acetate for the methanogenesis process. Currently, *Methanosaeta* and *Methanosarcina* are the only two methanogens known to perform acetoclastic methanogenesis (Kurade *et al*., 2020). It should be noted that *Methanosarcina* can use all three methanogenic pathways (Hobson and Stewart, 2013; Mizrahi *et al*., 2021). As methanogens are present in the calf rumen shortly after birth (Guzman *et al*., 2015), it can be theorized that changes to the metabolic environment as a result of development stage select for differing dominant methanogen functional groups. This knowledge may be critical in the development of CH_4_ reduction strategies involving microbial community manipulation, as well as the formulation of novel treatments.

**1.3 Protozoa and Fungi**

While less numerous than the bacterial community members, protozoa and fungi represent a significant portion of the rumen microbiome. First recognized in 1843 by Gruby and Delafond, protozoa have been found to make up anywhere from 20% to 50% of the microbial population within the rumen (Huws *et al*., 2018). Protozoa are introduced into the rumen of newborn animals via contact with their dam during the first two weeks of life (Becker and Hsiung, 1929; Yáñez-Ruiz *et al*., 2015). The role of ruminal protozoa is controversial, but recent evidence suggests that they play a role in organic matter degradation (Newbold *et al*., 2015). One key finding is that methanogens can colonize inside the protozoan residents and removal (i.e., defaunation) of protozoa can lead to a reduction in CH_4_ output at rates between 13-35% (Morgavi *et al*., 2012; Guyader *et al*., 2014; Mizrahi *et al*., 2021). Belanche *et al*. (2014) showed that protozoan hydrogenosomes produce H_2_ further indicating their potential role in CH_4_ production. Questions now arise as to which protozoan genera or species are linked to which archaeal methanogens and subsequent CH_4_ emissions. As with non-associated archaea, *Methanobrevibacter spp.* dominate the protozoan methanogenic populations (Tapio *et al*., 2017). Interestingly, differences have been observed in the abundance of protozoan methanogens and non-associated archaea (Tokura *et al*., 1997; Sharp *et al*., 1998; Newbold *et al*., 2015; Tapio *et al*., 2017). These observations indicate that protozoan methanogens and free-living methanogens perform distinct activities between the differing communities. Therefore, it can be speculated that the archaeal composition, not abundance, is more correlated with CH_4_ emissions. Compositional differences of archaea are also evident within protozoan associated methanogens. As discussed in previous reviews (Tapio *et al*., 2017), larger protozoan species are more heavily colonized by methanogens and bacteria compared to their smaller protozoan neighbors. Given this, evidence has shown that smaller protozoa are more correlated with CH_4_ production compared to larger species (Ranilla *et al*., 2007; Newbold *et al*., 1995). However, evidence to the contrary exists and coupled with other factors such as diet and feeding time, CH_4_ emissions were not found to be tightly associated with protozoan community dynamics (Stumm *et al*., 1982; Tapio *et al*., 2017). Complicating matters more, defaunation may result in less efficient animals and reintroduction of protozoa from the environment limit the usefulness of such a CH_4_ strategy. A better understanding of the relationship between protozoa-associated methanogens and the host ruminant is needed to understand the role they play in methanogenesis.

The other eukaryotic members of the ruminant microbiome are anaerobic fungi and studies estimate that fungal members comprise approximately 10-20% of the rumen microbial community (Rezaeian *et al*., 2004; Elekwachi *et al*., 2017; Huws *et al*., 2018). Paul *et al*. (2018) investigated the fungal members of the rumen and discovered that *Piromyces* was the most abundant culturable genera and *Buwchfawromcyes* was the least abundant. The study also concluded that there could be upwards of 25 additional genera which remain to be characterized (Paul *et al*. 2018). Anaerobic fungi, which thrive in the rumen environment, are effective fiber degraders due to their ability to efficiently produce a number of enzymes capable of degrading plant structural polymers as well as their rhizoids having plant structure penetration abilities (Solomon *et al*., 2016; Huws *et al*., 2018). The role of fungi in methanogenesis is less understood; however, it has been shown that their activity is enhanced in the presence of methanogens, leading to improved animal productivity (Huws *et al*., 2018). These findings suggest that fungi are important members of the rumen microbiome and while their role in CH_4_ production is not well understood, the crucial part they play in animal productivity maintains the need for their presence in the community. Further research is needed to fully understand the role ruminant fungi have in CH_4_ output and animal productivity.

**1.4 Drivers of Ruminant Microbial Composition and Diversity**

Microbial composition of the digestive tract is affected by many drivers and environmental influences. It has been established across varying species (e.g., humans, ruminants, etc.) that the primary deterministic driver of microbial community composition is diet. In humans, dietary variations result in both quantitative and qualitative changes in substrate availability to the microbial communities in the large intestine, which can result in altered microbial metabolism (Louis *et al*., 2007). Additionally, human diet can alter the relative abundance of taxa present in the gut microbiome (Turnbaugh *et al*., 2008; Turnbaugh *et al*., 2009; Spor *et al*., 2011). These findings also apply to ruminant microbial ecology and studies support this claim. For instance, Henderson *et al*. (2015) collected 742 samples from 32 species and sub-species of ruminants and determined that forage-based diets resulted in a higher abundance of unclassified *Bacteroidales* and *Ruminococcaceae* and concentrate-based diets revealed a higher abundance of *Prevotella* and unclassified *Succinivibrionaceae*. In addition, the microbiome structure of forage-fed animals was consistent and distinctly different from concentrate-fed animals. Other studies have shown that changes in dietary protein and carbohydrate content, supplementation of easily digestible carbohydrates, forage preservation, and different types of forage alter rumen microbial ecology (Fernando *et al*., 2010; Belanche *et al*., 2012; Huws *et al*., 2018; Newbold and Ramos-Morales, 2020).

Interestingly, a longitudinal study assessing the temporal changes during specific dietary periods showed that animal age, independent of diet or sex, had significant clustering effects on the rumen microbiome (Furman *et al*., 2020). Therefore, age of the animal is an important deterministic driver of microbial community assembly. Age can still be considered confounded as an independent driver due to the feeding nature of ruminant livestock operations. Overall, diet is the major driver for bacterial community structure in ruminants with age being a secondary determining factor. It should be noted that data suggests that dietary changes can be short-lived within adult animals because the rumen microbiome is highly resilient to perturbations as described by Weimer *et al*. (2017). Future studies should account for founder effects, a form of genetic drift where the frequency of a given genotype in a population changes due to stochastic sampling rather than due to selection, in which even a fraction of the original microbiome could re-establish itself and prevent the invasion of secondary community members (Moraïs and Mizrahi, 2019a). In this context, future studies on dietary interventions and diet-derived microbiome engineering could benefit by being implemented in early life ruminants. The early life ruminant microbiome is highly unstable and could allow for manipulation of microbial composition that may persist later in life (Yáñez-Ruiz *et al*., 2015; Huws *et al*., 2018). Early life dietary intervention steps could therefore result in a stable, healthy, and efficient microbiome that could continue throughout the growth of the animal.

Heritable genetic factors could result in co-selection mechanisms for microbial populations as well as microbial interactions that shape the development of the rumen. Therefore, heritability of the rumen microbiome should be investigated in the context of the holobiont concept. Within this theory of evolution, the hologenome is defined as the sum of all genetic material from the host and the microbiota, acting together as a unit, where symbiotic relationships between the host and microbes are essential for host fitness (Zilber-Rosenberg & Rosenberg, 2008). This concept has intriguing and ample supporting data within the context of heritability. Research has indicated that heritable species from two main orders, *Bacteroidetes* and *Firmicutes*, were represented in 50% to 100% of the 47 dairy cows examined with the majority being seen in 70% to 100% of all examined animals (Sasson *et al*., 2017). This same study also revealed that operational taxonomic units (OTU) from the genera *Bacteroidales*, *Prevotella*, *Clostridiales*, and *Flavefaciens* were highly correlated with productivity efficiency traits, including milk protein content and feed efficiency. Li *et al*. (2019) detailed that four unclassified bacterial genera were heritable keystone species that were linked to greater animal productivity and an increase in VFA production. In beef cattle, it was also reported that the rumen archaeal relative abundance is dictated by host genetic factors (Roehe *et al*., 2016). Wallace *et al*. (2019) investigated a cohort of 1,000 cows from different farms and determined that 39 microorganisms were indeed heritable and central in core microbiome networks with seven microorganisms being strongly correlated with CH_4_ production. The evidence from the studies discussed here not only display evidence for microbiome heritability, but they also provide key evidence that the host and its microbial populations are acting in sync and thus, suggests that the host drives evolutionary mechanisms upon the microbiota and vice versa. The long-lasting relationship that is established between the ruminant and its symbiotic microbes results in total dependance on one another (Zilber-Rosenberg, 2008). The ruminant system is an excellent candidate for holobiont co-evolution mechanisms research. It is therefore crucial that microbiome engineering studies for animal productivity as well as CH_4_ mitigation strategies consider the intertwined and important relationship between the animal and their microbial symbionts.

Microbiome heritability is not free of influence in and of itself. It was previously estimated that there are around 350 microbial species within the rumen (Edwards *et al*., 2004). With the advent of new sequencing technology, it is now estimated that there are more than 2,000 microbial species in the rumen microbial complex (Firkins, 2010; Cammack *et al*., 2018). Wallace *et al*. (2019) found that only 39 microbes, or < 2% of the total rumen microbial population, were heritable. Thus, most of the rumen microbial population is introduced through other means. Lack of inheritable microbes across taxa is not a surprising ecological perspective when considering there is evidence that the environment can shape microbial population structure and composition (Rashid *et al*., 2015; Koskella *et al*., 2017). Environmental factors affecting microbiota in ruminant livestock and animals include, but are not limited to, housing systems, rearing systems, geographical locations, and access to the dam. For example, Fonty *et al*. (1987) established that lambs fed a controlled diet kept in group housing had higher abundances of cellulolytic bacteria compared to lambs raised in solidarity. Weaning methods are important in rumen microbial composition, whereby the dam plays an essential role in microbial succession (Skillman *et al*., 2004). When young goats were allowed access to the doe, kids were found to have a different ruminal community structure and increased diversity, compared to kids reared in an artificial system (*i.e*., where the animals were separated from the doe immediately after birth; Abecia *et al*., 2017). Another important distinction between weaning methods was shown by Williams and Coleman (1992) where artificially reared animals demonstrated a near absence of protozoan colonization due to protozoa inoculation coming from direct contact with the saliva of the dam or other adult animals. In terms of methanogenesis reduction, this is an important finding in that defaunation of the protozoal population has resulted in a 13 to 35% reduction in methanogenesis (Morgavi *et al*., 2008; Morgavi *et al*., 2012; Belanche *et al*., 2015). Given these findings, environmental intervention strategies could prove viable in CH_4_ reduction techniques. However, mitigation strategies applied to young animals generally only results in weak persistence into adulthood: mitigation strategies conducted on animals after weaning may have a greater influence on rumen microbial ecology of mature animals (Newbold and Ramos-Morales, 2020). More research is needed to fully understand the influence of environmental drivers of microbial community composition and to elucidate new methods of enhanced CH_4_ reduction and productivity in livestock operations.

Other drivers of rumen microbial composition are breed and sex of the animal. Henderson *et al*. (2015) maintained that ruminant microbial composition is less effected by host biological factors; however, there is evidence to the contrary. For starters, in the same study unclassified *Veillonellaceae* were more abundant in sheep, deer, and camelids compared to cattle. It was speculated that differences in bacterial relative abundance were due to the size differences in foregut, animal anatomy, and frequency of feedings between the above-mentioned ruminant species compared with bovines (Hofmann, 1989; Henderson *et al*., 2015). Other studies found differences in microbial composition and diversity among different ruminant species. Host-derived mechanistic properties could alter rumen microbial ecology; the study detailed how when ruminal contents were swapped between cows, thereby exchanging microbial community structures, their bacterial composition reverted back to pre-exchange structures after a period of time. Founder effects could be responsible for the return to pre-exchange state where the remaining pre-exchange microbiome could create an alternative community state capable of self-reestablishment (Moraïs and Mizrahi, 2019). Recent evidence shows that animal breed and sex have a major role in rumen microbial composition. For example, when comparing the microbial ecology of water buffalo and Jersey dairy cattle under comparable feeding conditions, there were differences in bacterial, protozoan, and archaeal populations (Iqbal *et al*., 2018). It has also been shown that different breeds from the same species have distinct microbial diversities and compositions. Li *et al*. (2019) demonstrated that three different breeds of beef cattle (*i.e*., Angus, Charolais, and Kinsella composite hybrids) had differing bacterial and archaeal profiles. Rumen microbial diversity of Charolais cattle was lower than the other two breeds, with Angus being the most diverse.

Animal sex alters the rumen microbiome composition, as found in beef bulls, which have higher archaeal abundances and lower bacterial abundances than beef steers. In addition, bulls had higher bacterial alpha diversity levels (*i.e*., richness and evenness) and lower archaeal alpha diversity levels (Li *et al*., 2019). In humans, it was demonstrated using a mouse model that male castration eliminated the gut microbial differences between males and females and treatment with testosterone after castration prevented these microbial changes, further detailing the driving force of sex on microbial community structure (Yurkovetskiy *et al*., 2013). This supports the claim that sex is an important driver for rumen microbial ecology. While differences in sex influence microbial ecology, hormones do not have the same level of evidence. It has been well-established that hormone implants consisting of estradiol, progesterone, and/or testosterone can significantly improve beef cattle live and carcass weights (Parr, 2020). However, a recent study suggested that there were no differences in the microbial profiles between cattle subjected to a moderate hormone implant regime or an aggressive hormone implant regime (Henniger *et* al., 2022). The lack of estrogenic and androgenic receptors on the rumen epithelium could indicate that there is little opportunity for interaction between implant hormones and ruminant microbial life. Additionally, Henniger *et al*. (2022) revealed little difference between metabolites of the implant groups, pointing out that the differences that were seen were likely not due to bacterial and archaeal composition. It could be theorized that while no compositional differences were seen, taxonomic redundancy and masking could have hidden key metabolic differences between the implant groups. Therefore, future research should investigate metagenomic profiles to identify any differences in functional genes between moderate and aggressive implant strategies. Current and future microbiome engineering studies for CH_4_ mitigation and improved animal efficiency should consider animal breed, sex, and hormone differences in study design. However, researchers must consider diet as a potential confounder when discussing breed, sex, and hormone response.

1. **The Ruminant Microbiome and Methanogenesis**

**2.1 Methanogenesis**

The methanogenesis pathway occurs at the end of the ruminant electron flow and is activated by three different functional groups all using differing input metabolites. These functional groups come from phylogenetic groups that span across different orders all belonging to the same archaeal phylum, Euryarchaeota. Euryarchaeota is made up of seven different orders with the genus *Methanobrevibacter* of the order Methanobacteriales being the most dominant (Friedman *et al*., 2017). Methanogen functional groups include the hydrogenotrophs (Demirel and Scherer, 2008), methylotrophs (Mizrahi *et al*., 2021), and acetoclastic methanogens (Ferry, 1999; Morgavi *et al*., 2010). Hydrogenotrophs constitute most of the methanogens in mature animals and utilizes [H+] to reduce CO_2_, CO, or formate to CH_4_ (Furman *et al*., 2020). Hydrogenotrophic methanogenesis is thermodynamically favorable to the other two pathways which results in lower abundances of other methanogenic organisms (Fenchel *et al*., 2012; Pereira *et al*., 2022). This understanding is consistent with *Methanobrevibacter* being the most dominant methanogenic genera. Methylotrophic methanogenesis produces CH_4_ using methylated substrates such as trimethylamine, dimethyl sulfate, and methanol (Lyu *et al*., 2018). Methylotrophs represent the highest abundance of methanogens in newborn calves and utilize methylated compounds such as methanol or methyl amines to produce CH_4_ (Furman *et al*., 2020). It has been documented that the methylotrophic *Methanosphaera* was negatively correlated with CH_4_ emissions in both beef and dairy cattle (Cunha *et al*., 2017; Cunha *et al*., 2018; Ramayo-Caldas *et al*., 2020; Pereira *et al*., 2022). These findings could be explained by the variation of the stoichiometric properties between the hydrogenotrophic and methylotrophic pathways that result in hydrogenotrophic methanogenesis being energetically favored (Cunha *et al*., 2017; Pereira *et al*., 2022). Finally, acetoclastic methanogens utilize acetate for the methanogenesis process. Currently, *Methanosaeta* and *Methanosarcina* are the only two methanogens known to perform acetoclastic methanogenesis (Kurade *et al*., 2019). It should be noted that *Methanosarcina* can use all three methanogenic pathways (Hobson and Stewart, 2012; Mizrahi *et al*., 2021). As methanogens are present in the calf rumen shortly after birth (Guzman *et al*., 2015) it can be theorized that different metabolic environments present at different stages of development select for differing dominant methanogen functional groups. This knowledge may be critical in the development of CH_4_ reduction strategies involving microbiome community manipulation, as well as the formulation of novel treatments.

**2.2 Current Methane Mitigation Strategies**

Methane is a potent GHG with a global warming potential that is 28-34 times higher than CO_2_ per unit mass and on a 20-year time scale, it is 82 times higher than that of CO_2_ (Edenhofer, 2015; Carnachan *et al*., 2019). While only 3-5% of total GHG emissions are credited to livestock and agriculture, the increased demand for livestock protein products will inevitably increase CH_4_ emissions (Gerber *et al*., 2011; Resinger *et al*., 2021). While there are major concerns for greenhouse effect, CH_4_ inhibition has also been connected to improved animal productivity (Grainger *et al*., 2011; Hristov *et al*., 2015; Mizrahi *et al*., 2021). Past research has indicated that methanogenesis can result in a gross energy loss of 2-12% and reduced feed efficiency (Johnson and Johnson, 1995; Yu *et al*., 2021). Due to CH_4_’s environmental impact and potential reduction in animal production, there has been an increase in research into CH_4_ mitigation and reduction strategies (Supplementary Figure 1). These research efforts are imperative for the sustainability of livestock agriculture and to address global climate change. Many of these mitigation strategies and techniques have been thoroughly reviewed and therein, describe in detail the mode of action, affects, implementation routes, and complications associated with these methods (Huws *et al*., 2018; Abbott *et al*., 2020; Beauchemin *et al*., 2020; Min *et al*., 2021; Mizrahi *et al*., 2021; Yu *et al*., 2021; Bačėninaitė *et al*., 2022; Glasson *et al*., 2022; Beauchemin *et al*., 2022). The following sections aims to summarize current CH_4_ mitigation strategies within the context of how these techniques and tools affect the ruminant microbiome, to further expand on early life microbiome engineering techniques, and to examine the effects of microbiome changes to animal health and performance attributable to CH_4_ reduction strategies.

**2.3 Inhibitory Compounds**

Inhibitory compounds traditionally have targeted methanogens or CH_4_ production specifically by directly affecting one or more enzymatic steps of methanogenesis (Mizrahi *et al*., 2021). One such compound is 3-nitrooxypropanol (3-NOP) which disrupts methanogenesis by binding to the CH_4_-producing enzyme methyl-coenzyme M reductase (MCR) thereby inhibiting the formation of CH_4_ (Hristov *et al*., 2015; Duin *et al*., 2016). Yu *et al*. (2021) comprehensively details the use of 3-NOP for CH_4_ mitigation within ruminant livestock. One of the more unique aspects of 3-NOP is the high specificity it has for methanogens (Beauchemin *et al*., 2020). Several studies have maintained that while 3-NOP can reduce 16S rRNA gene copy numbers as well as the relative abundance of methanogens, the compound also decreased total microbial alpha diversity but with limited effects on bacteria (Haisan *et al*., 2016; Martinez-Fernandez *et al*., 2018). Zhang *et al*. (2020a) additionally demonstrated 3-NOP use in beef heifers resulted in a reduction in alpha diversity within the microbial community, specifically reducing methanogens, without significant changes to community composition. It is therefore reasonable to infer that while 3-NOP will reduce the alpha diversity and relative abundance of the microbial community members, the only major taxonomic group specifically negatively affected are methanogens. Given this understanding, it is also inferable that a reduction in methanogens may result in a loss of diversity of bacteria because distinct positive interactions between bacteria and archaea have been documented.

Henderson *et al*. (2015) identified positive associations between some less abundant bacteria and archaea; the study posited that this interaction was due to the ability of some bacteria, such as *Succinivibrio* spp. and *Lachnospiraceae* spp., to degrade pectin into methanol, a substrate required for the growth of the methanogenic species *Methanomassiliicoccaceae* and *Methanospaera*. Therefore, it is not surprising to see a reduced alpha diversity in 3-NOP treated animals because of the interplay between the differing domains of life. Recently, Gruninger *et al*. (2022) provided evidence that compositionality changes may indeed occur due to the use of 3-NOP. Their work indicated that within rumen fluid samples, control samples clustered separately from 3-NOP treated samples, indicating there are community composition effects when treating animals with the inhibitory compound. While these findings illustrate the diversity and compositional dynamics when using 3-NOP, it is important to understand the functional differences induced by 3-NOP supplementation. Studies have indicated that while MCR gene abundance was not different between control Holstein dairy cows and 3-NOP supplemented cows, gene copies were lower in the treated group (Pitta *et al*., 2022). This reduction in gene expression led to a clear methanogenesis inhibition. A previous study in sheep demonstrated the same effect on gene expression (Shi *et al*., 2014), demonstrating a clear shift in functionality when using 3-NOP. These studies, coupled with the understanding that different inhibitory doses are required for different methanogenic lineages (Duin *et al*., 2016; Pitta *et al*., 2021), provide evidence that 3-NOP-induced methanogenesis inhibition varies between individual methanogenic lineages (Pitta *et al*., 2022). When analyzing the findings of all these studies, it becomes necessary for future research to investigate the factors involved with lineage specific mechanisms induced by 3-NOP as well as continuing to elucidate metagenomic details involved in the microbial food web in 3-NOP-supplemented animals.

Another inhibitory compound is an algae-derived halogenated CH_4_ analogue (HMAs), particularly bromoform. The red-seaweed genus *Asparagopsis*, primarily *A. taxiformis* and *A. armata*, are of particular interest, especially considering that their mitigation potential of enteric CH_4_ is much greater than other seaweed species (e.g., brown seaweeds; Beck et al., 2022; Reyes et al., 2023). The *Asparagopsis* seaweeds have been documented for their ability to produce and encapsulate a small, yet sufficiently active quantity of HMAs leading to CH_4_ inhibition and have been extensively investigated (Li *et al*., 2019; Roque *et al*., 2019a; Kinley *et al*., 2020; Roque *et al*., 2019a; Roque 2019b; Stefenoni *et al*., 2021; Glasson *et al*., 2022). Additionally, the use of algae supplementation and the subsequent effects and consequences have been comprehensively reviewed elsewhere (Abbott *et al*., 2020; Min *et al*., 2021; Bačėninaitė *et al*., 2022). As with 3-NOP, bromoform from *Asparagopsis* spp. have high specificity for effecting methanogens and it has been further shown in an *ex vivo* study of the rumen microbiome that inhibition of methanogenesis precedes methanogenic relative abundance as well as having no measurable effect on SCFA production (Roque *et al*., 2019b; Mizrahi *et al*., 2021). Machado *et al*. (2018) indicated a significant decrease in methanogen relative abundance in an *in vitro* experiment when using rumen fluid from cannulated Brahman steers while demonstrating a clear reduction in CH_4_ production. However, Roque *et al*. (2019b) demonstrated a significant reduction in CH_4_ production yet, it was not in concert with the decrease in average methanogen abundance, albeit a reduction in Euryarchaeota was reported.

It has been recently shown that methanogenesis inhibition was accompanied by a decline in overall microbial activity and bacterial diversity (O’Hara *et al*., 2023). Because disrupting the core microbiome can lead to negative impacts for the ruminant, supplementation with *Asparagopsis* spp. could lead to a decrease in prominent fiber degraders and VFA producers which could result in negative animal performance (Neu *et al*., 2021; O’Hara *et al*., 2023). Roque *et al*. (2019b) additionally posits that the ‘Anna Karenina’ hypothesis (**Box 1**) is at play with algae supplementation wherein, the hypothesis theorizes that microbiome perturbations result in microbial composition differentiation (Zaneveld, *et al*., 2017). While studies have shown no effect on animal performance (Min *et al*., 2021) as well as studies indicating improved performance parameters (Abecia *et al*., 2012; Singh *et al*., 2015; Roque *et al*., 2019a), negative effects from algae supplementation should be further investigated given more recent findings (O’Hara *et al*., 2023). The findings above agree that algae supplementation results in marked CH_4_ reduction. However, there is no clear relationship between the algal-induced CH_4_ reduction and archaeal relative abundance as well as algae supplementation’s effect on animal health and performance. Because changes in microbial diversity and composition due to disturbances are a known phenomenon, Anna Karenina principles should be considered when evaluating animal performance and algae supplementation to potentially reveal the functional dynamics between the microbiome and methanogenesis inhibition. It has been suggested that large-scale changes to the microbiome are not a prerequisite for functional alterations in the ruminant microbial ecosystem (Roque *et al*., 2019b). While Choi *et al*. (2022) revealed no significant differences in predicted function between red seaweed extract supplementation and control groups, there is relatively little information regarding functionality between algae supplementation and the rumen microbiome. More extensive research is required to determine functional differences in algae-treated and steady-state ruminant microbiomes for the use of algae as a CH_4_ mitigation approach.

**2.4 Nutrition**

As previously mentioned, diet is the major driver for microbial community composition for ruminant microbial composition. Dietary manipulations have been investigated for CH_4_ mitigation and many reviews are available that provide detail on dietary interventions for methanogenesis inhibition (Beauchemin *et al*., 2008; Hristov *et al*., 2013; Knapp *et al*., 2014; Beauchemin *et al*., 2020; Beauchemin *et al*., 2022). The difference between forage-based and concentrate-based diets is a core concept for diet formulation in ruminants. The capability of ruminants to produce high-quality protein from forages reduces competition for grains between humans. Additionally, forage diets provide other ecologically important benefits, such as improving soil health, enhancing water quality, and promoting and conserving biodiversity within the environment (Guyader *et al*., 2016; Beauchemin *et al*., 2020). A major concern with forage-based ruminant systems is that 75% of global ruminant CH_4_ emissions are produced from forage diets of grazing animals (Beauchemin *et al*., 2020). The cellulosic material in forage enhances methanogenesis as fiber fermentation favors acetate production providing a hydrogen source and thereby increasing CH_4_ production. Improving forage quality and digestibility can to some extent reduce CH_4_ although an absolute reduction of CH_4_ (g/day/head) may not always be seen (Beauchemin *et al*., 2009; Beauchemin *et al*., 2020; Beauchemin *et al*., 2022). For instance, higher quality forages have greater ratios of non-fiber carbohydrates to neutral detergent fiber promoting organic matter degradation. This process results in more [H^+^] available for methanogenesis and an increase in DMI promotes greater absolute CH_4_ output. Interestingly, an increase in DMI is associated with a greater rate of passage within the rumen resulting in reduced CH_4_ yield (g per kg of DMI). Furthermore, greater DMI results in increased performance, thereby reducing CH_4_ emission intensity (g CH_4_ per unit of production) (Beauchemin *et al*., 2020). Overall, there is variation within the net CH_4_ reduction from enhanced forage quality.

Forage-based diets are commonly supplemented with concentrates (i.e., feeds that are rich in energy and protein and low in fiber). It is a common practice in commercial livestock settings to feed animals high concentrate-based diets as they are energetically more efficient. Oltjen (2019) demonstrated this as the efficiency of metabolized energy use for grain decreased with an increase in roughage inclusion. This efficiency is attributed to fermentation of starches from concentrate-diets resulting in more propionate and butyrate while cellulose fermentation results in more acetate. Acetate is converted to ketone bodies that can be used for energy or can enter the tricarboxylic acid (TCA) cycle via oxaloacetate condensation. For the latter process, oxaloacetate requires propionate, glucogenic amino acids, lactate, or glycerol. In high-concentrate diets, there is more proprionate available for acetate to enter the TCA cycle. Because the TCA cycle yields more ATP compared with other acetate metabolism pathways, acetate entering the TCA cycle results in greater animal efficiency (Van Soest, 1994; Johnson and Johnson, 1995; Kobayashi, 2010). Conveniently, this process represents an alternative H_2_ sink potentially resulting in decreased CH_4_ production. Theoretically, supplementing forage-based diets with concentrates could increase nutrient supply for use in more metabolically beneficial pathways for ruminant livestock to fulfill nutritional gaps and increasing performance as well as reducing CH_4_ emissions. For example, Thompson et al. (2019) reported an 8% reduction in enteric CH_4_ emissions per unit of ADG with increasing energy supplementation to beef cattle grazing winter wheat pastures. Snelling *et al*. (2019) additionally reported animals fed a high-concentrate diet had significantly lower CH_4_ emissions, an increase in propionate production, better animal productivity, and changes in bacterial community composition. Other studies have further pointed out this difference between forage and concentrate diets in Holstein cows (Wang *et al*., 2020). Interestingly, research has indicated that the use of other CH_4_ mitigation strategies coupled with concentrate diets is synergistic. Sunflower oil fed combined with a concentrate-based diet resulted in decreased daily CH_4_ emissions (g/d or g/kg OM digested) in lactating dairy cows (Bayat *et al*., 2017). The methanogenesis inhibitor 3-NOP showed increased CH_4_ reduction potential in dairy cows fed a high-concentrate diet while decreasing in effectiveness over time when added to a high-forage ration (Schilde *et al*., 2021). The incorporation of both concentrates and other CH_4_ mitigation strategies could provide a valuable on-farm management technique.

While it is known that concentrate-based diets reduce CH_4_ emissions and can improve animal performance, high concentrate diets can impact microbial composition, diversity, and functionality. Marked differences were seen in microbial composition (i.e., beta-diversity) in Holstein cattle fed a high forage-based diet or a high concentrate-based diet indicating vast differences in the bacterial communities between the two groups (Wang *et al*., 2020)HHols. Decreases in microbial alpha-diversity are often associated with dysbiosis which can result in negative outcomes for the host species. This was shown in research that indicated a significant decrease in Shannon’s diversity in goats fed a high concentrate diet resulting in abnormal fermentation patterns (Hua *et al*., 2017). These results indicate a pattern of significant diversity and compositional differences between cattle fed high concentrate-based diets resulting in dysbiotic microbial populations. Additional studies have reported that an increase in dietary concentrate inclusion resulted in dysbiosis within the microbial community with potential community functional loss (Russell and Rychlik, 2001; Khafipour *et al*., 2011; Li *et al*., 2012).This phenomenon was demonstrated in a study analyzing the bacterial communities of Angus cows where an increase in concentrates was proportional to an undesirable increase in the Firmicutes-to-Bacteroidetes ratio (Chen *et al*., 2021). Bacteroidetes are known to have a higher mean of glycoside hydrolase and polysaccharide lyase compared to Firmicutes, making Bacteroidetes spp. the primary degraders of complex polysaccharides (Meale *et al*., 2016). Moreover, Zhang *et al*. (2020b) reported a decrease in Bacteroidetes and an increase in Firmicutes at the phylum level in dairy cattle fed high concentrate diets. Therefore, based on these studies it can be inferred that an increase in concentrates could disrupt key bacterial species that would likewise be in a steady-state condition in a high-forage based diet, which could affect animal performance or health via functional changes within the microbiome. Additional evidence is required to confirm this theory.

Both the negative and positive effects of concentrate-based diets should be considered when formulating livestock diets due to the potential drawbacks on the rumen microbiome from concentrates. One solution could be the type or quality of forage used in feed for commercial livestock settings. A recent study compared the bacterial community compositions and animal productivity between three different forages all supplemented with concentrates and found that Holstein bulls fed oats haylage or vetch haylage had an increased bacterial diversity, unique community composition, and more robust microbial networks compared to animals fed forages such as barley straw. Robust microbial networks have been associated with fewer incidences of ruminal acidogenesis (González *et al*., 2012; Costa-Roura *et al*., 2020). Although not significantly different, the animals fed the oat haylage were also associated with higher animal performance metrics (primarily ADG). Interestingly, cattle fed vetch haylage resulted in a decreased ADG although demonstrating a far more robust microbial network compared to barley straw. Therefore, forage source must be considered along with a potential trade-off between microbial robustness and lower ADG when formulating livestock feeds.

Using a high-starch forage alongside concentrate supplementation could prove a viable strategy to inhibit methanogenesis as well as enhance animal performance. Forages such as corn silage or small-grain cereals increase starch and decrease fiber concentrations resulting in propionate production; as previously mentioned, propionate production competes with methanogenesis for [H+] inhibiting methanogens while also lowering rumen pH (Beauchemin *et al*., 2022). This principle was demonstrated by Gislon *et al*. (2020) wherein dairy cattle fed corn silage diets (49.3 % DM corn silage) produced higher propionate proportion compared to a diet comprised of Italian ryegrass hay and alfalfa hay (25.3 % DM each); the study also reported a significant decrease in CH_4_ production (g/d). These findings are supported by previous research indicating that increasing starch content of silages, such as corn silage, can reduce CH_4_ production (Hassanat *et al*., 2013). Functional analysis has revealed that there is some degree of functional redundancy seen between forage selection and concentrate supplementation (Taxis *et al*., 2015; Costa-Roura *et al*., 2020). Despite this, Cousta-Roura *et al*. (2020) demonstrated that quality forages presented unique lactate-producing populations, primarily *Sharpea, Bifidobacterium, Eubacterium, Selenomonas,* and *Olsenella*. These species could provide a functional means to reduce acidosis in the rumen, dictating microbial interactions that prove beneficial to the animal. For example, *Bifidobacterium pseudolongum* is a lactic acid-producing bacterial species that has been previously shown to improve body weight gain and feed efficiency along with reduced incidences of diarrhea in newborn calves (Abe *et al*., 1995; Seo *et al*., 2010; Ban and Guan, 2021). Future studies should investigate the functional understanding of these species and the underlying mechanisms in which they interact within microbial communities driven by higher concentrate-based diets. The findings therein could elucidate critical approaches in CH_4_ mitigation while maintaining animal performance or ruminal microbial composition for improved cost/profit for the livestock operation.

Another avenue for nutritionists to consider is supplementing fat sources within the diet. Most ruminant diets consist of 5% fat in total dry matter with linoleic acid and α-linolenic acid being the major fatty acids in forage and concentrate diets (Enjalbert *et al*., 2017). While fat supplementation was originally implemented to improve dietary energy requirements for cattle in intensive farming systems, it has been reported that dietary fatty acid supplementation can mitigate enteric CH_4_ emissions. Fat sources that have been studied for their anti-methanogenic properties include soybeans (Enjalbert *et al*., 2017), sunflower seeds (Beauchemin *et al*., 2007), cottonseed (Belk *et al*., 2018), linseed (Martin *et al*., 2016), and soybean oil (Beck et al., 2019). It has been posited that fat supplementation may reduce CH_4_ emissions via three mechanisms. As previously mentioned, research has demonstrated that saturation of long-chain polyunsaturated fatty acids can decrease methanogenesis by providing an alternative metabolic hydrogen acceptor in the reduction of CO_2_; however, methanogenesis inhibition has been attributed to a reduction in substrate availability for CH_4_ production using this mode of action (Czerkawski *et al*., 1966; Johnson and Johnson, 1995). Secondly, it has been hypothesized that fat supplementation might reduce DMI or fiber digestibility resulting in a net reduction of CH_4_ emissions (Eugène *et al*., 2008; Rabiee *et al*., 2012; Hristov *et al*., 2013; Beck *et al*., 2018). Thirdly, the rumen microbiome could be altered which impacts rumen function, mitigating CH_4_ production (Martin *et al*., 2016; Enjalbert *et al*., 2017; Belk *et al*., 2018). However, a primary concern with fat supplementation is the inhibitory effects of fat on bacterial growth. As previously discussed, (see Alternative H_2_ Sinks), unsaturated fatty acids are considered toxic to microbial cells. Biohydrogenation of unsaturated fatty acids is a detoxification process due to the negative effects of unsaturated fatty acids on bacterial cell growth (Maia *et al*., 2007; Jarvis and Moore, 2010; Yang *et al*., 2019). It has been reported that *Fibrobacter*, *Ruminococcus*, *Butyrivibrio*, and *Prevotella* are negatively affected *in vivo* by linoleic acid and α-linoleic acid (Enjalbert *et al*., 2017; Li *et al*., 2020). A recent study further hypothesized that accumulation of unsaturated fatty acids could decrease feed intake and fiber digestibility due to the inhibition of *Butyrivibrio* as high feed intake cattle had lower concentrations of linoleic acid and α-linolenic acid (Li *et al*., 2020; Arndt *et al*., 2022; Beauchemin *et al*., 2022). A main hurdle in understanding these inhibitory effects of unsaturated fatty acids can be attributed to the differing species, functions, and metabolic pathways of the various bacteria affected within the microbiome. Additionally, these inhibitory effects of dietary fat supplementation cannot be categorically defined as the root cause of a reduction in bacterial relative abundance because a change in diet could reflect alteration in nutrient and function distribution among the ruminant microbial members (Enjalbert *et al*., 2017). Therefore, there is a need for metagenomic mechanistic research to elucidate the properties of the microbiome that results in both CH_4_ reduction and biohydrogenation of fat supplements, as well as providing new insights into how fats improve animal performance.

While research efforts into the ecological effects on ruminant microbes is limited, studies have indicated the ability of certain fat sources, such as canola oil, in association with the inhibitory compound 3-NOP to significantly alter composition and diversity in ruminant microbial populations (Gruninger *et al*., 2022). Specifically, this study identified that there was a marked shift in composition between control samples, canola oil-only samples, and canola oil plus 3-NOP samples indicating that the addition of canola oil alone can alter microbial structure. The combination of 3-NOP and canola oil resulted in a dramatic reduction in the mean relative abundance of archaeal populations and not surprisingly, the CH_4_ emissions from these samples was substantially reduced. Additionally, it has been shown that supplementing a concentrate-based diet with a lipid source (i.e., rapeseed cake 190 g/kg DM) resulted in significantly lower CH_4_ emissions on a g/kg DMI basis (Troy *et al*., 2015; Snelling *et al*., 2019). The findings in these studies indicate the ability of added fat in combination with other CH_4_ intervention products to have an enhanced effect as opposed to using either method individually. This insight could prove extremely valuable for future researchers and producers alike in improving and innovating new synergistic CH_4_ reduction strategies.

**2.5 Alternative H_2_ Sinks**

A primary concept in CH_4_ reduction is directing [H+] away from methanogenesis to other beneficial H_2_ sinks. Metabolic hydrogen is a key molecule involved in CH_4_ production and overall rumen community dynamics. During plant fiber fermentation, [H+] is produced and is utilized by methanogens during the methanogenesis process (Mizrahi *et al*., 2021). Accumulation of H_2_ has been posited to have detrimental effects on the fermentation process due to high H_2_ partial pressure within the rumen inhibiting certain microbial dehydrogenases which reduce fermentation, dry matter intake (DMI), and the digestibility of feeds (Janssen, 2010; Leng, 2014). However, it appears as though the rumen is capable of H_2_ partial pressure maintenance through other pathways even when methanogens are inhibited (Martinez-Fernandez *et al*., 2018; Yang *et al*., 2019). While methanogenesis is the largest H_2_ sink in the rumen, there is evidence that [H+] can be redirected into other reduced end-products other than CH_4_ and H_2_ such as microbial mass (Ungerfeld, 2015; Martinez-Fernandez *et al*., 2018). Conversely, recent studies concluded that CH_4_ reduction strategies via direct alteration of methanogen diversity or abundance could result in ruminant H_2_ accumulation; given this, there could be negative effects on microbial species that are sensitive to H_2_ partial pressure (Mizrahi *et al*., 2021; Glasson *et al*., 2022). Consequently, CH_4_ reductions strategies should account for the redirection of [H+] into nutritionally beneficial H_2_ sinks (**Box 1**) for the improvement of animal productivity. Several of these alternative H_2_ sinks have been investigated including homoacetogenesis, fumarate reduction, nitrite reduction, sulfate reduction, and biohydrogenation of unsaturated fatty acids.

Alternative H_2_ sinks such as homoacetogenesis, fumarate reduction, nitrite reduction, and sulfate reduction have been investigated. Homoacetogens are bacteria that produce acetate through the acetyl-CoA pathway and are known to occur naturally within the rumen environment (Kelly *et al*., 2022). The primary acetogens belong to the families *Lachnospiraceae*, *Clostridiaceae*, and *Ruminococcaceae* and degrade multiple substrates including H_2_ (Pereira *et al*., 2022). While acetogens are thought to be the first hydrogenotrophs to colonize the early-life ruminant (Morvan *et al*., 1994), methanogens likely outcompete acetogens as methanogenesis is thermodynamically favorable compared to acetogenesis. However, recent evidence suggests that hydrogenotrophic acetogenesis may be more significant as an H_2_ sink than previously recognized. Greening *et al*. (2019) in a metagenomic and metatranscriptomics study of sheep rumen contents demonstrated that 74% of the reads were predicted to be hydrogenotrophic acetogens, mainly *Blautia, Acetitomaculum*, and *Oxobacter*. While acetogenesis has a higher threshold for H_2_ utilization, increasing the partial pressure within the rumen headspace could improve the thermodynamic favorability of acetogenesis (Ungerfeld, 2013; Pereira *et al*., 2022). As mentioned previously, an increase in H_2_ partial pressure can have adverse effects on the microbial communities and performance of the ruminant. However, acetogens can also degrade formate. While there is little known about the significance of formate as an electron carrier, it has been posited that formate synthesis could play an important role in the control of H_2_ partial pressure (Leng, 2014). When methanogens are slightly inhibited, formate is produced in bulk and organisms that possess formate dehydrogenases can then rapidly convert formate to H_2_ and CO_2_ (Doetsh *et al*., 1953; Thiele *et al*., 1988; Leng, 2014). Therefore, when methanogenesis is inhibited, the production of formate and its subsequent association with the ruminant microbial community could ensure a low H_2_ concentration (Leng, 2014). The role of acetogens in both H_2_ and formate degradation requires more thorough investigation to elucidate the practicality of acetogen supplementation for methanogenesis inhibition.

Additionally, increasing propionate levels offers potential as an effective H_2_ sink. Greening *et al*. (2019) reported that, second to only methyl-CoM reductase, the 1d [NiFe]-hydrogenase of Selenomonadales was the most expressed H_2_ uptake hydrogenase. The *Selenomonas* species has previously been shown to grow via hydrogenotrophic fumarate reduction and nitrate ammonification facilitated by the 1d [NiFe]-hydrogenase (Henderson, 1980; Martin and Park, 1996; Iwamoto *et al*., 2002; Greening *et al*., 2019). Fumarate is a precursor for propionate, which consumes H_2_ via the succinate and acrylate fermentation pathways resulting in significantly less H_2_ available for CH_4_ production (McAllister and Newbold, 2008; Kobayashi, 2010; Xue *et al*., 2021; Pereira *et al*., 2022). A recent study evaluated the effects of fumarate supplementation in rumen fluid and found that inclusion of fumarate significantly increased propionate production and decreased the acetate:propionate ratio (Xue *et al*., 2021). Decreasing acetate:propionate results in both decreased CH_4_ and increased energy retention within ruminants (Wolin, 1960; Russell, 1998). Choi *et al*. (2022) further demonstrated that red seaweed supplementation in the rumen fluid of non-lactating Hanwoo cows resulted in an increase in propionate proportions 24 h after supplementation. Other *in vitro* studies have indicated the addition of the fumarate-reducing bacteria *Mitsuokella jalaludinii* resulted in lower methanogen DNA copies and occurrence (Mamuad *et al*., 2014; Pereira *et al*., 2022). Additionally, it has been shown *in vivo* that supplementing the propionate-forming bacteria *Lactobacillus pentos* D31 as a probiotic resulted in a 13% reduction in CH_4_ production through two weeks of administration (Jeyanathan *et al*., 2016). Collectivley, the findings suggest that propionate production from fumarate-reducing bacteria or propionate-forming bacteria can outcompete methanogens for H_2_ availability.

Other alternative H_2_ sinks include nitrate and sulfate reduction and biohydrogenation of unsaturated fatty acids. Nitrate and sulfate reducing bacteria are native members of the ruminant microbiome and have been investigated for their potential as alternative H_2_ sinks. Both nitrate-reducing bacteria (*Wolinella succinogenes* and *Selenomonas ruminantium*) and sulfate-reducing bacteria (genera *Desulfovibrio* and *Desulfotomaculum*) offer alternative H_2_ sinks as both nitrate and sulfate reduction are more thermodynamically favorable than methanogenesis (Lan and Yang, 2019). The population of these bacteria have been reported to be at low relative abundances within the ruminant microbial population; however, their abundances increase as the availability of their perspective electron acceptors increases in concentration (Lan and Yang, 2019; Kelly *et al*., 2022). The inclusion of nitrate and sulfate in ruminant diets has previously been identified as a potential means to lower CH_4_ production (Leng, 2014). However, there is risk of toxicity associated with nitrate and sulfate dietary inclusion. The nitrate conversion to nitrite has been previously recognized as having toxic effects on ruminal microorganisms (Latham *et al*., 2016). Sulfate reduction also has adverse health effects as there is risk of polioencephalomalacia, which occurs from hydrogen sulfide (H_2_S) accumulation within the rumen headspace and inhalation of H_2_S (Gould, 1998; van Zijdervald *et al*., 2010). Therefore, considerable caution must be taken when considering the use of nitrate and sulfate supplementation for CH_4_ mitigation.

Biohydrogenation of unsaturated fatty acids is considered a detoxification process as unsaturated fatty acids are considered more toxic to microbial cells than saturated fatty acids (Maia *et al*., 2007; Jarvis and Moore, 2010; Yang *et al*., 2019). Biohydrogenation of unsaturated fatty acids results in conjugated linoleic acid and vaccenic acid that enter meat and milk products of ruminants and have been shown to provide general health benefits to humans (Givens and Shingfield, 2006; Yang *et al*., 2019). It has been previously demonstrated that long-chain polyunsaturated fatty acids provide an alternative metabolic hydrogen acceptor to the reduction of CO_2_ thus decreasing methanogenesis (Czerkawski *et al*., 1966; Johnson and Johnson, 1995). However, the total amount of [H+] used in the biohydrogenation of unsaturated fatty acids is small (1%) in comparison to that of methanogenesis and CH_4_ reduction of studies supplementing fat sources was attributed to a limitation in substrate available for methanogenesis (48%; Czerkawski *et al*., 1966; Johnson and Johnson, 1995). More recent research has indicated that all members of the genera *Butyrivibrio* and *Pseudobutyrivibrio* carry out biohydrogenation of unsaturated fatty acids (Paillard *et al*., 2007; Lourenço *et al*., 2010; Yang *et al*., 2019). Given this, supplementing both an unsaturated fatty acid source and *Butyrivibrio*/*Pseudobutyrivibrio* species as a direct-fed microbial could provide an alternative H_2_ sink and an indirect method of methanogenesis reduction. Previous research has indicated that providing *Butyrivibirio fibrisolvens* DSM 2071 increased total gas production while not increasing CH_4_ production *in vitro*, providing evidence that *Butyrivibrio* spp. can have anti-methanogenic properties (Jeyanathan *et al*., 2016). However, *Butyrivibrio* is highly affected by the toxicity of unsaturated fatty acids on bacteria and therefore, careful consideration must take place in formulating CH_4_ mitigation strategies as proposed above. One potential method is the inclusion of lactate in a proposed unsaturated fatty acid:*Butyrivibirio/Pseudobutyrivibirio* methanogenesis inhibition strategy. Lactate has previously been found to protect *Eubacterium ruminantium* from the toxic effects of linoleic acid indicating a possible metabolic link between lactate and fatty acid biohydrogenation (Maia *et al*., 2007). Further research is warranted to address these claims.

When discussing hydrogenation and alternative H_2_ sinks, interspecies H_2_ transfer must be considered. Interspecies H_2_ transfer typically refers to the transfer of H_2_ from fermenting organisms to methanogens allowing for the conversion of HCO_3_^-^ to CO_2_ and H_2_O, which is subsequently converted into CH_4_ by methanogens (Leng, 2014). This transfer of H_2_ between H_2_ producers and utilizers often occurs in tight, organized biofilms that optimize interspecies localization resulting in a rapid rate of CH_4_ production (McAllister *et al*., 1994; de Bok *et al*., 2004; Leng, 2014). Interestingly, alternative H_2_ pathways were shown to be upregulated in low CH_4_-emitting sheep when compared to high CH_4_-emitting sheep where methanogenesis predominated (Greening *et al*., 2019; Kelly *et al*., 2022). Additionally, Martínez-Álvaro *et al*. (2020) demonstrated that there are important differences in microbial diversity, abundance, and interspecies interactions between low CH_4_-emitting cattle and high CH_4_-emitting cattle. Primarily, high CH_4_ emitters had a lower number of hydrogenotrophic methanogenic species with limited interactions compared to low CH_4_ emitting animals, where methanogens were more diverse, were involved in all three methanogenic pathways, and had more interactions with other microbial communities. The differences in H_2_ sinks and microbial composition between low- and high-emitting animals could be due to the availability of substrates produced in the differing microbial consortia. The substrates produced coupled with the distance between organisms involved in interspecies transfer could explain which species is most successful in a given community. These interactions could also dictate the partial pressure of H_2_ influencing the fermenting reaction (e.g., methanogenesis, acetogenesis, etc.) that takes place (Leng, 2014). Taken together, there is evidence that interspecies H_2_ transfer and alternative H_2_ sinks are dependent on the microbial species within a given ruminant community or more specifically, the functionality of those microbes. As previously mentioned, there is significant functional redundancy and taxonomic masking that occurs in the rumen (Weimer, *et al*., 2015; Moraïs and Mizrahi, 2019a). Thus, there is difficulty in fully understanding the true mechanistic and metabolic processes that govern these reactions.

**2.6 Animal Breeding and Early Life Interventions**

One novel method in mitigating CH_4_ emission is breeding and genetic programs designed to select for low CH_4_-emitting animals. Because CH_4_ emission is a heritable trait (Herd *et al*., 2014; Mizrahi *et al*., 2021), breeding programs have been established that select for animals with small rumen sizes and thus, lower nutrient particle retention times (Ørskov *et al*., 1988; Mizrahi *et al*., 2021). However, these breeding programs are known to reduce animal feed efficiency making this selection strategy less desirable (NZAGRC, 2019; Mizrahi *et al*., 2021). Given that recent studies have identified that the heritable microbiome along with host genetic and immunological factors are connected to both rumen microbiome composition and function, it can be hypothesized that breeding programs should select for animals with desirable microbiomes (Xiang *et al*., 2016; Roehe *et al*., 2016; Martínez-Álvaro *et al*., 2020; Maman *et al*., 2020; Meale *et al*., 2021). For example, Wallace *et al*. (2015) and Roehe *et al*. (2016) demonstrated in two different studies that microbial genes involved in methanogenesis were significantly greater in high CH_4_-emitting animals. These two studies further showed that *Succinovibrionaceae* was a major player in low-emitting animals as these bacterial species produce succinate as their main fermentation product, which utilizes [H+] rather than releasing it as H_2_ for methanogenesis utilization (Wallace *et al*., 2015). Additionally, network analysis studies have shown that more robust and diverse microbial networks are associated with lower-emitting animals (Martínez-Álvaro *et al*., 2020). Therefore, using the rumen microbial profile for low CH_4_ emissions as a breeding selection criterion could prove a valuable resource in current and future breeding programs. Future studies should address the mechanistic aspects of the microbial networks involved in low-emitting ruminants for critical knowledge in developing these programs.

There has been a recent push for evaluating microbiome manipulation in early-life ruminants for CH_4_ mitigation. As previously discussed, early life microbiomes of ruminants are quite unstable, which allows for the potential for microbial engineering practices to be applied for methanogenesis inhibition. Interestingly, the initial colonizers of the rumen microbiome have recently been shown to have long-lasting effects on the assembly process in adult microbial populations (Furman *et al*., 2020). However, a primary question in early life intervention strategies is the window of time that would allow for microbial interventions to be most effective. Past research has indicated that ruminant bacterial structure reaches a level of stability between 3 and 4 weeks of age, suggesting a critical window of opportunity for CH_4_ mitigation (Rey *et al*., 2013; Abecia *et al*., 2014; Guzman *et al*., 2015; Yáñez-Ruiz ­*et al*., 2015). Additionally, it was shown that weaning and weaning age have dramatic effects on microbial diversity and composition regardless of weaning strategy further suggesting another critical window for intervention (Meale *et al*., 2016; Meale *et al*., 2017). Therefore, the age of the animal must be considered when designing novel microbiome engineering strategies. Early-life microbiome engineering offers a promising avenue of CH_4_ reduction as it provides an intervention method that can be applied infrequently (i.e., early in life) with long-term CH_4_ mitigation effects.

Microbiome engineering studies in early life are also concerned with long-term treatment effects as several studies have indicated a return to pre-treatment ecologies following a cessation of the treatment (Romero-Pérez *et al*., 2016). Although limited in the number of current studies, recent research has aimed to address these questions of temporal stability. Meale *et al*. (2021) demonstrated that 3-NOP supplemented from day of birth through 14 weeks persistently reduced CH_4_ emissions in dairy calves. The supplementation of 3-NOP at both transitional periods (i.e., microbial establishment and weaning) modulated the rumen environment such that long lasting (as much as 46-weeks after 3-NOP supplementation ended) effects were seen on CH_4_ output (Meale *et al*., 2021; see above discussion on 3-NOP). Coupled with this finding, it could be beneficial to enhance diet formulation in early life intervention techniques to select low CH_4_-producing microbiomes. Furman *et al*. (2020) saw an increase in Methanobacteriaceae and a decrease in the hydrogen-utilizing *Succinivibrionaceae* family when switching to a fiber-rich diet from a high-starch diet. This finding is in line with the understanding that concentrates can reduce methanogen abundance and the subsequent release of CH_4_ (see “Nutrition” section). Collectively, these findings suggest that microbiome manipulation in early life via inhibitory compound supplementation and proper diet formulation could be an innovative strategy for methanogenesis intervention. An increase in attention to the mechanistic knowledge of microbiome assembly and ruminant host-microbiome interactions could provide key evidence in the development of early life intervention strategies. As such, it is imperative for future research to increase studies focused on functionality of microbiome assemblage at early life stages. Such knowledge would prove beneficial to the understanding and development of microbiome engineering practices for CH_4_ inhibition.

Another strategy for early life intervention is targeting the protozoal populations in a process called defaunation. As previously discussed, protozoa play a major role in methanogenesis as they are an appreciable source of H and methanogens often colonize protozoa. Several studies have indicated the ability of defaunation to effectively reduce CH_4_ emissions by 13-43% (Hegarty *et al*., 1999 Morgavi *et al*., 2008; Hegarty *et al*., 2008; Morgavi *et al*., 2012; Guyader *et al*., 2014; Belanche *et al*., 2015). Moreover, clear differences in microbial community composition have been demonstrated in defaunated ruminants. Belanche *et al*. (2015) demonstrated a clear shift in compositionality between fauna-free sheep, holotrich protozoa inoculated sheep, total-faunated sheep with each treatment showing distinct separation. The study also demonstrated a positive correlation of methanogens with holotrich protozoa inoculated sheep and a negative correlation with CH_4_ emissions with fauna-free sheep. These results indicate that there is a solid correlation between methanogenesis and protozoa presence. One possible explanation given for the increase in methanogen numbers in holotrich protozoa inoculated sheep is due to no competition in colonization for the protozoa (Belanche *et al*., 2015). The effectiveness of defaunation has been challenged however, as a recent meta-analysis indicated only a 10% reduction in CH_4_ emission (Arndt *et al*., 2022). Additionally, the total removal of protozoa is challenging as reinoculation occurs rapidly via cross contamination in commercial livestock operations (Beauchemin *et al*., 2022). It is reasonable to infer that defaunation could be a viable method for CH_4_ reduction albeit, this process is highly challenging and the effects on total microbial ecology are contentious. Further research is warranted to elucidate the potential of defaunation’s viability and practicality in a commercial setting for CH_4_ emission reduction.

**2.7 Methanotrophs**
Another intriguing area of research is the use of other microorganisms to reduce CH_4_. There is evidence that some bacterial species, known as methanotrophs, can oxidize CH_4_. Since 1906, it was believed that methanotrophy was only present in the bacterial phylum Proteobacteria (Whittenbury *et al*., 1970; Hanson and Hanson, 1996; Guerrero-Cruz *et al*., 2021). Within the last two decades however, it has been determined that methanotrophs encompass both bacteria and archaea (Dedysh and Knief, 2018; Guerrero-Cruz *et al*., 2021). Methanotrophs can survive in both aerobic and anaerobic conditions. Bacterial methanotrophic species are classified as either Type-1 (Gammaproteobacteria) or Type-II (Alphaproteobacteria). It has been reported that Type-1 methanotrophs prefer environments higher in oxygen (O_2_) and lower in CH_4_ and vice verso for Type-2 methanotrophs (Smith and Murrell, 2009; Rani *et al*., 2024). Oxidation of CH_4_ by methanotrophs is reliant on the enzyme methane monooxygenase (MMO) to incorporate O_2_ molecules into CH_4_ as eloquently described elsewhere (Ahmadi and Lackner, 2024; Rani *et al*., 2024).

Interestingly, methanotrophs have been shown to be native to the rumen and thus, could provide a natural system in mitigating enteric CH_4_ emissions (Mitsumori *et al*., 2002; Finn *et al*., 2012). Within anaerobic conditions, methanotrophs utilize various terminal electron acceptors such as sulfate, nitrate, nitrite and metals (Soren *et al*., 2015). Interestingly little work has been done to elucidate the effectiveness of anaerobic methanotrophs to reduce CH_4_. Kajikawa *et al*. (2003) reported possible low observations of methanotrophy in ruminal fluid. The use of these methanotrophs as a direct-fed microbial for CH4 mitigation has been suggested by Jeyanathan *et al*. (2014). However, the lack of animal studies investigating this method leave much to be discerned. Additionally, it has been reported that the methanotrophic genus *Methylomonas* is negatively correlated with CH_4_ production (Auffret *et al*., 2018; Beauchemin *et al*., 2020). More recently, Sharifi *et al*. (2022) elucidated that methanotrophs increased when nitrate supplementation was applied to a high forage diet. The study suggests that CH_4_ emissions can be reduced by methanotrophs without drastically altering native microbial populations. The combined studies suggest that combining other means of CH_4_ mitigation techniques (specifically, nitrate and sulfate supplementation – see above) could prove a viable alternative strategy. However, more research is needed to confirm the effectiveness of methanotrophs in reducing enteric CH_4_.

**2.8 Other Methane Inhibitory Strategies**

Other techniques and strategies for CH_4_ mitigation have been investigated which include supplementation of carbon fermentation intermediates (Ungerfeld *et al*., 2003; Ungerfeld *et al*., 2007; Foley *et al*., 2009), dietary fat inclusion (Czerkawski et al., 1966; Grainger et al., 2008; Beauchemin et al., 2007; Beck et al., 2018; Beck et al., 2019), vaccines (Subharat *et al*., 2015; Zhang *et al*., 2015), essential oils (Benchaar and Greathead, 2011; Cobellis *et al*., 2016; Hristov *et al*., 2022), tannins (Aboagye and Beauchemin, 2019), saponins (Adegbeye *et al*., 2019), and ionophores (Appuhamy *et al*., 2013; Arikan *et al*., 2018). There are comprehensive reviews that detail in extensive fashion the mode of action and research related to effectiveness of these alternative strategies (Mizrahi *et al*., 2021; Beaucehmin *et al*., 2022) and therefore, these topics will not be discussed here.

Vaccination strategies are under development but have been met with a level of difficulty. While studies have identified antibodies in serum and saliva produced from methanogenic vaccines, the effect of these antibodies *in vivo* have shown little or no effect on CH_4_ production (Baca-González *et al*., 2020). Studies in sheep have demonstrated varied results in terms of methane reduction *in vitro* and *in vivo*. *In vitro* studies indicated that sheep inoculated with a methanogenic vaccine and a 28-day booster resulted in a 12.8% - 26.26% CH_4_ reduction (Baker and Perth, 2000). Wright (2004) vaccinated sheep with a mix of three methanogens resulting in a 7.7% - 12.8% reduction in CH_4_ *in vivo*. One of the key issues with vaccine development is the between-animal variation of the rumen microbiome as well as the need to produce a vaccine that targets all methanogenic species as to ensure no methanogenic species can proliferate and fulfill that ruminant niche (Beauchemin *et al*., 2022; Volmer *et al*., 2023). However, recent advances in the knowledge of the core microbiome’s heritability could prove a viable course of action in vaccine development. One plausible vaccine development method stems from the understanding that a core microbiome is inherited and begins colonizing the early life ruminant mere hours after birth. A hypothesis arising from this review is that vaccines targeting the early life archaeal populations could have long-lasting effects on microbial ecology (see above for discussion on early life interventions for further context). Additional research is needed to substantiate this supposition as well as to identify functional niches that would allow the rumen to be susceptible to vaccines.

Secondary plant compounds, such as tannins and saponins have been suggested to be a promising means to design animals for reduced environmental impacts (Beck and Gregorini, 2021), have been extensively evaluated and reviewed for their enteric CH_4_ reduction potential (Hassan *et al*., 2020; Dhanasekaran *et al*., 2020; Beauchemin *et al*., 2022). Tannins are secondary metabolites of plants and are chemically divided into either hydrolyzable or condensed. Hydrolyzable tannins are commonly found in low amounts within fruit seeds such as pomegranate, blackberries, and myrobalan. Condensed tannins are the most common tannin group and are found in trees and shrubs. A comprehensive list of tannins and their sources can be found in a recent review on tannins’ impact in animal nutrition (Besharati *et al*., 2022). Results from the supplementation of tannins have been variable concerning methanogenesis inhibition, animal performance, and overall microbial ecological effect. While several studies have indicated that tannin supplementation can reduce CH_4_ by up to 30% (Tavendale *et al*., 2005; Adejoro *et al*., 2019; Hassan *et al*., 2020), other studies have demonstrated adverse effects on animal performance (Faixova and Faix, 2005; Bodas *et al*., 2012; Dhanasekaran *et al*., 2020), making the use of tannins for CH_4_ mitigation controversial. However, there is likely an appropriate dose of tannins that have minimal negative impacts on animal performance, while still reducing enteric CH_4_ (Beck and Gregorini, 2021). Additionally, tannins source and their chemical composition affect animal performance and the ruminant microbiome differently. In fact, hydrolysable tannins are considered more suitable compared to that of condensed tannins. Previous research has indicated that gallic acid, a subunit of hydrolysable tannins, has the potential to reduce CH_4_ and NH_3_ emissions, maintain animal performance, and inhibit undesirable microorganisms such as *Clostridium, Listeria,* and *Escherichia coli* (Aboagye *et al*., 2019; He *et al*., 2020; Hassan *et al*., 2020). However, condensed tannins have been reported to inhibit both beneficial rumen bacteria (e.g., *Ruminococcus flavefaciens*) and methanogenic archaea (Bae *et al*., 1993; Bhatta *et al*., 2009; Hassan *et al*., 2020). In one recent study, tannin supplementation in lambs has been shown to increase the diversity and abundance of butyrate-producing and other beneficial bacteria such as *Bifidobacterium* and *Lactobacillusamino* (Corrêa *et al*., 2020). Studies have also revealed that there are no structural changes to the microbiome although richness was diminished in tannin-supplemented cattle (Díaz Carrasco *et al*., 2017). While it is clear that the use of tannins in the diet can reduce methanogenesis, further research is vital to increase our understanding of the effects tannins have on animal performance and microbial ecology. Primarily, the source and chemical structures of tannins must be considered when assessing their effects on ruminant microbial ecology. Additionally, little research has been done to date utilizing tannins in combination with other CH_4_ mitigation strategies and thus, these types of studies are needed.

Saponins have also been linked to variable results regarding methanogenesis inhibition. While saponins mediate rumen fermentation by decreasing protein degradation and urea and ammonia concentrations, there has been a weak correlation between saponin supplementation and CH_4_ reduction (Hassan *et al*., 2020). For example, Goel *et al*. (2008) investigated the effects of saponin supplementation in forage and concentrate based diets and determined that there was a decrease in protozoal populations and methanogen populations although there was no associated decrease in methanogenesis. It is posited that enhanced expression of genes from certain methanogenic species compensate for the reduction of methanogen relative abundance and therefore, methanogenesis does not decrease (Henderson *et al*., 2018). However, previous studies have indicated that tea saponins (3 g/d) resulted in a 10.6% - 27.7% reduction in CH_4_ production (Mao *et al*., 2010; Zhou *et al*., 2011). These studies demonstrated a significant reduction in protozoal populations which could account for the decrease in CH_4_. Additional studies have indicated the ability of tea saponins to reduce *Lachnospiraceae* in alfalfa and soybean hull-based fiber diets fed to cattle (Wang *et al*., 2019). *Lachnospiraceae* spp. have previously been linked to high-CH_4_ emitting sheep (Kittelmann *et al*., 2014; Tapio *et al*., 2017). Therefore, saponins have shown the ability to reduce methanogenesis by modulating various members of the ruminant microbial consortia. These studies highlight the importance of studies investigating gene expression and interactions within the microbiome to enhance nutrient utilization and animal efficiency when accounting for tannin or saponin supplementation for the control of methanogenesis (Hassan *et al*., 2020). In broader terms, future research should highlight methodologies in understanding functionality and gene expression within the rumen microbial community, especially protozoa and H_2_-producing bacteria, as diversity and composition might not account for complete CH_4_ inhibition or reduction.

1. **Animal Performance, Health, and Methane Inhibition**

**3.1 Alpha Diversity and Dysbiosis**

A key, yet perplexing concept in ruminant microbial ecology is that lower microbial alpha diversity (i.e., richness) has recently been linked to higher feed efficiency (Ben-Shabat *et al*., 2016). Traditionally, an increase in microbial diversity is associated with healthy gut microbiomes as a loss of diversity can lead to dysbiosis and can be indicative of disease states. Theoretical and empirical data have long suggested that a higher species diversity is associated with more stable microbial communities with greater pathogenic resistance (Loreau *et al*., 2003; Decaestecker *et al*., 2013; Downing *et al*., 2014; Karkman *et al*., 2017). An increase in richness (i.e., the number of observed microbial species) is further associated with more efficient resource utilization and an increase in functional redundancy (Fargione *et al*., 2008; Karkman *et al*., 2017). However, in the ruminant system, there appears to be a counter-intuitive process related to microbial richness (Supplementary Figure 2). An increase in feed efficiency, which is indicative of high-performing animals yielding higher grade product, is theorized to be correlated with lower richness thus, a simpler microbial network which leads to higher concentrations of specific nutrients that are used to support the host’s energy requirements (Ben-Shabat *et al*., 2016; Díaz Carrasco *et al*., 2017). Snelling *et al*., (2019) corroborated this thought process by modulating diets to higher amounts of concentrates which resulted in decreased CH_4_ emissions, increased animal performance, and a lower Shannon’s index. However, it was recently shown that an increase in high concentrate diets lead to an increase in mastitis in dairy cows (Hu *et al*., 2022). Indeed, in other gastrointestinal (GI) environments such as the lower GI tract, lower bacterial diversity has also been associated with dysbiosis and disease (Bartels *et al*., 2010; Ma *et al*., 2020; Kim *et al*., 2021). Recently, it was shown that in early-life dairy calves a decrease in richness, evenness, and Shannon’s Diversity was indicative of calf scour’s onset, an important economic disease of dairy calves (Frazier, 2021). A possible explanation for the variation between microbial environments could be differences in environment-specific microbial composition and specific host-mediated functions that account for microbial population structure. Ben-Shabat *et al*. (2016) characterized this claim by suggesting that the diversity decrease was due to the lower abundance of methanogens and thus, methanogenic pathways without significant changes to overall microbial composition. In communities subjected to perturbation via disease, significant changes were seen not only in alpha diversity, but also in community composition as a clear shift could be seen in early life dairy calf microbiomes between diseased and healthy animals (Frazier, 2021). Therefore, these findings suggest that from an ecological perspective, processes such as methanogenesis, which are performed by small taxonomic groups like methanogens, are more sensitive to changes in richness (Hooper *et al*., 1995; Ben-Shabat *et al*., 2016). However, Hu *et al*. (2022) demonstrated that dairy cow mastitis seen in cows fed a high-concentrate based diet was linked to a significant decrease in ruminant richness as well as milk and feces richness, raising a question of diet-derived CH_4_ reduction strategies using concentrates.

**3.2 Stress and Methanogenesis**

Taken together, the above experiments point to a significant debate in whether methanogenesis inhibition equates to a net positive for the host animal. As previously mentioned, a reduction in diversity due to methanogen-specific inhibitory compounds, such as 3-NOP, is not surprising due to the nature of interplay between archaea and bacteria within microbial networks (Henderson *et al*., 2015). The issue surrounding a decrease in microbial alpha diversity revolves around the significant correlation between disease state and decreased diversity and richness. Another significant issue is the microbial response to stress. Few studies have indicated that heat stress can alter microbial populations (Tajima *et al*., 2007; Uyeno *et al*., 2010; Chen *et al*., 2018; Zhao *et al*., 2019; Baek *et al*., 2020). Additional studies have indicated microbial population changes as well as higher cortisol levels associated with CH_4_ production in beef cattle following transport stress (Llonch *et al*., 2016; Deng *et al*., 2017). While these findings indicate that stress may have an impact on microbial communities, it is important to note that it is difficult to differentiate stress related changes from feed intake. Therefore, stress and disease state can alter microbial profiles; however, dietary influences could mask the effects seen in CH_4_ emissions. Indeed, future research should investigate not only diversity and composition effects of CH_4_ mitigation strategies, but also functional aspects of these techniques related to animal performance and health. The information that could be elucidated therein would be valuable to animal livestock operations and researchers alike as new understandings of how CH_4_ inhibition techniques and products affect both the host animal and the global environment.

**3.3 Ionophores**

The use of ionophores is another important line of investigation for animal health and CH_4_ mitigation. Given the public health concern over antibiotic use in livestock animals, there has been a shift away from non-therapeutic antibiotic use in production animals. In 2006 the European Union banned the use of antibiotics for any reason other than treating severe illness (Franz *et al*., 2010). Regardless, antibiotics, specifically ionophores, have shown the ability to increase animal performance and efficiency by shifting the microbial population toward gram-negative bacteria resulting in higher concentrations of propionate that can compete with methanogens for H_2_ (Russell and Houlihan, 2003; Almeida *et al*., 2021). Monensin is among the most used ionophore in ruminant animals to increase feed efficiency as feed intake is reduced by 5-6% while not hindering ADG (Johnson and Johnson, 1995). However, the use of monensin as a CH_4_ inhibitor appears to be a small co-benefit if beneficial at all. A recent meta-analysis showed minimal CH_4_ yield reduction (4%) and modest CH_4_ emission intensity reduction (0% - 14%; Almeida *et al*., 2021). Monensin acts as an indirect inhibitor of methanogenesis and does not directly inhibit methanogens; instead, monensin inhibits H_2_-producing bacteria and as a result CH_4_ output can be reduced (Russell and Houlihan, 2003; Shen *et al*., 2017). Nevertheless, using ionophores such as monensin might not be potent enough on their own to be used as CH_4_ mitigators. Additionally, as a consequence of continued use of monensin, the rumen microbiome can adapt and become resistant, reducing the ionophore response including any CH_4_ reduction seen (Callaway *et al*., 2003; Almeida *et al*., 2021). Therefore, using ionophores ass a primary means for CH_4_ reduction has limited capacity. The combination of ionophores and other mitigation techniques could prove beneficial and future research should investigate this claim.

1. **References**

Abbott, DW, Aasen, IM, Beauchemin, KA, Grondahl, F, Gruninger, R, Hayes, M, et al. Seaweed and seaweed bioactives for mitigation of enteric methane: Challenges and opportunities. *Animals*. 2020;10:2432. <https://doi.org/10.3390/ani10122432>

Abe, F, Ishibashi, N and Shimamura, S. Effect of administration of bifidobacteria and lactic acid bacteria to newborn calves and piglets. *J. Dairy Sci.* 1995;78. 2838-2846. <https://doi.org/10.3168/jds.S0022-0302(95)76914-4>

Abecia, L, Toral, PG, Martin-Garcia, AI, Martinez, G, Tomkins, NW, Molina-Alcaide, E, Newbold, C and Yanez-Rui, A. Effect of bromochloromethane on methane emission, rumen fermentation pattern, milk yield, and fatty acid profile in lactating dairy goats. *J. Dairy Sci*. 2012;95. 2027-2036. <https://doi.org/10.3168/jds.2011-4831>

Abecia, L, Ramos-Morales, E, Martínez-Fernandez, G, Arco, A, Martín-García, AI, Newbold, CJ and Yáñez-Ruiz, DR Feeding management in early life influences microbial colonisation and fermentation in the rumen of newborn goat kids. *Anim. Prod. Sci*. 2014;54. 1449-1454. <https://doi.org/10.1071/AN14337>

Abecia, L, Jiménez, E, Martínez-Fernandez, G, Martín-García, AI, Ramos-Morales, E, Pinloche, E, et al. Natural and artificial feeding management before weaning promote different rumen microbial colonization but not differences in gene expression levels at the rumen epithelium of newborn goats. *PLoS ONE*. 2017;12:e0182235. <https://doi.org/10.1371/journal.pone.0182235>

Aboagye, IA and Beauchemin, KA. Potential of molecular weight and structure of tannins to reduce methane emissions from ruminants: A review*. Animals* 2019a;9:856. <https://doi.org/10.3390/ani9110856>

Aboagye, IA, Oba, M, Koening, KM, Zhao, GY and Beauchemin, KA. Use of gallic acid and hydrolyzable tannins to reduce methane emission and nitrogen excretion in beef cattle fed a diet containing alfalfa silage. *J. Anim. Sci.* 2019b;97. 2230-2240. <https://doi.org/10.1093/jas/skz101>

Adegbeye, MJ, Elghandour, MM, Monroy, JC, Abegunde, TO, Salem, AZ, Barbabosa-Pliego, A and Faniyi, TO. Potential influence of Yucca extract as feed additive on greenhouse gases emission for a cleaner livestock and aquaculture farming – A review. *J. Clean Prod.* 2019;239:118074. <https://doi.org/10.1016/j.jclepro.2019.118074>

Adejoro, FA, Hassen, A and Akanmu, AM. Effect of lipid-encapsulated acacia tannin extract on feed intake, nutrient digestibility and methane emission in sheep. *Animals*. 2019;9:863. <https://doi.org/10.3390/ani9110863>

Ahmadi, F and Lackner, M. Recent findings in methanotrophs: genetics, molecular ecology, and biopotential. Appl. Microbiol. Biotech. 2024;108:60. <https://doi.org/10.1007/s00253-023-12978-3>

Almeida, AK, Hegarty, RS and Cowie, A. Meta-analysis quantifying the potential of dietary additives and rumen modifiers for methane mitigation in ruminant production systems. *Anim. Nutri.* 7. 2021;1219-1230. <https://doi.org/10.1016/j.aninu.2021.09.005>

Appuhamy, JADRN, Strathe, AB, Jayasundara, S, Wagner-Riddle, C, Dijkstra, J, France, J and Kebreab, E. Anti-methanogenic effects of monensin in dairy and beef cattle: A meta-analysis. *J. Dairy Sci*. 2013;96:5161-5173. <https://doi.org/10.3168/jds.2012-5923>

Arikan, OA, Mulbry, W, Rice, C and Lansing, S. The fate and effect of monensin during anaerobic digestion of dairy manure under mesophilic conditions. *PLoS ONE*. 2018;13:e0192080. <https://doi.org/10.1371/journal.pone.0192080>

Arndt, C, Hristov, AN, Price, WJ, McClelland, SC, Pelaez, AM, Cueva, SF, et al. Full adoption of the most effective strategies to mitigate methane emissions by ruminants can help meet the 1.5^o^C target by 2030 but not 2050. *Proc. Natl. Acad. Sci.* *USA*. 2022;119:e2111294119. <https://doi.org/10.1073/pnas.2111294119>

Auffret, MD, Stewart, R, Dewhurst, RJ, Duthie, C-A, Rooke, JA, Wallace, RJ, Freeman, TC, Snelling, TJ, Watson, M and Roehe, R. Identification, comparison, and validation of robust rumen microbial biomarkers for methane emissions using diverse *Bos taurus* breeds and basal diets. *Front. Microbiol*. 2018;9:2642. <https://doi.org//10.3389/fmicb.2017.02642>

Baca-González, V, Asensio-Calavia, P, Gonzalez-Acosta, S, Perez de la Lastra, JM and Morales de la Nuez, A. Are vaccines the solution for methane emissions from ruminants? A systematic review. *Vaccines*. 2020;8:460. <https://doi.org/10.3390/vaccines8030460>

Bačėninaitė, D, Džermeikaitė, K and Antanaitis, R. Global warming and dairy cattle: How to control and reduce methane emission. *Animals* 2022;12:2687. <https://doi.org/10.3390/ani12192687>

Bae, HD, McAllister, TA, Muir, AD, Yanke, LJ, Bassendowski, KA and Cheng, KJ. Selection of a method of condensed tannin analysis for studies with rumen bacteria. *J. Agri. Food Chem.* 1993;41:1256-1260. <https://doi.org/10/1021/jf00032a018>

Baek, YC, Choi, H, Jeong, J-Y, Lee, SD, Kim, MJ, Lee, S, Ji, S-Y and Kim, M. The impact of short-term acute heat stress on the rumen microbiome of Hanwoo steers. J. Ani. Sci. Technol. 2020;62:208-217. <https://doi.org/10.5187/jast.2020.62.2.208>

Baker, SK and Perth, W. Method for improving utilization of nutrients by ruminant or ruminant-like animals. U.S. Patent 6,036,950, 14 March 2000.

Ban, Y and Guan, LL. Implication and challenges of direct-fed microbial supplementation to improve ruminant production and health. *J. Anim Sci. Biotech.* 2021;12:109. <https://doi.org/10.1186/s40104-021-00630-x>

Bartels, CJ, Holzhauer, M, Jorritsma, R, Stewart, WA and Lam, TJ. Prevalence, prediction and risk factors of young Dutch dairy calves. *Prev. Vet. Med.* 2010;93:162-169. <https://doi.org/10.1016/j.prevetmed.2009.09.020>

Bayat, AR, Ventto, L, Kairenius, P, Stefański, T, Leskinen, H, Tapio, I, et al. Dietary forage to concentrate ratio and sunflower oil supplement alter rumen fermentation ruminal methane emissions, and nutrient utilization in lactating cows. *Transl. Anim. Sci.* 2017;1:277-286. <https://doi.org/10.2527/tas2017.0032>

Beauchemin, KA, McGinn, SM and Petit, HV. Methane abatement strategies for cattle: Lipid supplementation of diets. *Can. J. Anim. Sci.* 2007;87:431-440. <https://doi.org/10.4141/CJAS07011>

Beauchemin, KA, Kreuzer, MO, O’Mara, F and McAllister, T. Nutritional management for enteric methane abatement: A review. *Aust. J. Exp. Agric.* 2008;48:21-27. <https://doi.org/10.1071/EA07199>

Beauchemin, KA, Ungerfeld, EM, Eckard, RJ and Wang, M. Review: Fifty years of research on rumen methanogenesis: lessons learned and future challenges for mitigation. *Animals* 2020;14:S1:S2-S16. doi:10.1017/S1751731119003100

Beauchemin, KA, Ungerfeld, EM, Abdalla, AL, Alvarez, C, Arndt, C, Becquet, P., et al. Invited Review: Current enteric methane mitigation options. *J. Dairy Sci*. 2022;105:9297-9326. <https://doi.org/10.3168/jds.2022-22091>

Beck, MR, Thompson, LR, Williams, GD, Place, SE, Gunter, SA and Reuter, RR. Fat supplements differing in physical form improve performance but divergently influence methane emissions of grazing beef cattle. *Anim. Feed Sci. Technol*. 2019;254:114210. <https://doi.org/10.1016/j.anifeedsci.2019.114210>

Beck, MR and Gregorini, P. Animal design through functional dietary diversity for future productive landscapes. *Front. Sustain. Food Sys.* 2021;5:546581. <https://doi.org/10.3389/fsufs.2021.546581>

Beck, MR, Thompson, LR, Campbell, TN, Stackhouse-Lawson, KA and Archibeque, SL. Implied climate warming contributions of enteric methane emissions are dependent on the estimate source and accounting methodology. *Appl. Anim. Sci.* 2022a;38:639-647. <https://doi.org/10.15232/aas.2022-02344>

Beck, MR, Garrett, K, Fleming, AE, Maxwell, TMR, Greer, AW, Bunt, C, et al. Effects of *Lactobacillus* fermented plant products on dairy cow health, production, and environmental impact. *Anim. Feed Sci. Technol*. 2022b;294:115514. <https://doi.org/10.1016/j.anifeedsci.2022.115514>

Beck, MR, Thompson, LR, Rowntree, JE, Thompson, TN, Koziel, JA, Place, SE and Stackhouse-Lawson, KR. U.S. manure methane emissions represent a greater contribution to implied climate warming than enteric methane emissions using the global warming potential methodology. *Front. Sustain. Food Sys.* 2023;7:1209541. <https://doi.org/10.3389/fsufs.2023.1209541>

Becker, ER and Hsiung, TS. The method by which ruminants acquire their fauna of infusoria and remarks concerning experiments on host specificity of these protozoa. *Proc. Natl. Acad. Sci. U.S.A.* 1929;15:684-690. <https://doi.org/10.1073/pnas.15.8.684>

Belanche, A, Doreau, M, Edwards, JE, Moorby, JM, Pinloche, E and Newbold, CJ. Shifts in the rumen microbiota due to the type of carbohydrate and level of protein ingested by dairy cattle are associated with changes in rumen fermentation. *J. Nutr*. 2012;142:9:1684-1692. <https://doi.org/10.3945/jn.112.159574>

Belanche, A, de la Fuenta, G and Newbold, CJ. Study of methanogen communities associated with different rumen protozoal populations. *FEMS Microbiol. Ecol*. 2014;90:663-677. <https://doi.org/10.1111/1574-6941.12423>

Belanche, A, de la Fuente, G and Newbold, CJ. Effect of progressive inoculation of fauna-free sheep with holotrich protozoa and total-fauna on rumen fermentation, microbial diversity, and methane emissions. *FEMS Microbiol*. *Ecol*. 2015;91:fiu026. <https://doi.org/10.1093/femsec/fiu026>

Benchaar, C and Greathead, H. Essential oils and opportunities to mitigate enteric methane emissions from ruminants. *Anim. Feed Sci. Technol*. 2011;166-167:338-355. <https://doi.org/10.1016/j.anifeedsci.2011.04.024>

Ben-Shabat, SK, Sasson, G, Doron-Faigenboim, A, Durman, T, Yaacoby, S, Miller, MEB, et al. Specific microbiome-dependent mechanisms underlie the energy harvest efficiency of ruminants. *ISME J.* 2016;10:2958-2972. <https://doi.org/10.1038/ismej.2016.62>

Besharati, M, Maggiolino, A, Palangi, V, Kaya, A, Jabbar, M, Eseceli, H, et al. Tannin in ruminant nutrition: Review. *Molecules.* 2022. <https://doi.org/10.3390/molecules27238273>

Bhatta, R, Uyeno, Y, Tajima, K, Takenaka, A, Yabumoto, Y, Nonaka, I, Enishi, O and Kurihara, M. Difference in the nature of tannins on *in vitro* ruminal methane and volatile fatty acid production and on methanogenic archaea and protozoal populations. *J. Dairy Sci.* 2009;92:11:5512-5522. <https://doi.org/10.3168/jds.2008-1441>

Bodas, R, Prieto, N, Garcia-Gonzalez, R, Andres, S, Giraldez, FJ and Lopez, S. Manipulation of rumen fermentation and methane production with plant secondary metabolites. *Anim. Feed Sci. Technol*. 2012;176:78-93. <https://doi.org/10.1016/j.anifeedsci.2012.07.010>

Callaway, T, Edrington, T, Rychlik, J, Genovese, K, Poole, T, Jung, YS, et al. Ionophores: their use as ruminant growth promotants and impact on food safety. *Curr. Issues Intest. Microbiol.* 2003;4:43-51.

Cammack, KM, Austin, KJ, Lamberson WR, Conant, GC and Cunningham, HC. Ruminant Nutrition Symposium: Tiny but mighty: the role of the rumen microbes in livestock production. *J. Ani. Sci.* 2018;96:752-770. <https://doi.org/10.1093/jas/skx053>

Carnachan, SM, Bell, TJ, Hinkley, SF and Sims, IM. Polysaccharides from New Zealand native plants: a review of their structure, properties, and potential applications. *Plants*. 2019;8:163. <https://doi.org/10.3390/plants8060163>

Chen, S, Wang, J, Peng, D, Li, G, Chen, J and Gu, X. Exposure to heat-stress environment affects the physiology, circulation levels of cytokines, and microbiome in dairy cows. Sci. Rep. 2018;8:15606. <https://doi>.org/10.1038/s41598-018-32886-1

Chen, H, Wang, C, Huasai, S and Chen, A. Effects of dietary forage to concentrate ratio on nutrient digestibility, ruminal fermentation and rumen bacterial composition in Angus cows. 2021;11:17023. <https://doi.org/10.1038/s41598-021-96580-5>

Choi, Y, Lee, SJ, Kim, HS, Eom, JS, Jo, SU, Guan, LL, et al. Red seaweed extracts reduce methane production by altering rumen fermentation and microbial composition *in vitro*. *Front. Vet. Sci.* 2022;9:85824. <https://doi.org/10.3389/fvets.2022.985824>

Cobellis, G, Trabalza-Marinucci, M and Yu, Z. Critical evaluation of essential oils as rumen modifiers in ruminant nutrition: A review. *Sci Total Environ.* 2016;545-546 :556-568. <https://doi.org/10.1016/j.scitotenv.2015.12.103>

Corrêa, PS, Mendes, LW, Lemos, LN, Crouzoulon, P, Niderkorn, V, Hoste, H, et al. 2020. Tannin supplementation modulates the composition and function of ruminal microbiome in lambs infected with gastrointestinal nematodes. *FEMS Microbiol. Ecol*. 96:fiaa024. <https://doi.org/10.1093/femsec/fiaa024>

Costa-Roura, S, Balcells, J, de la Fuente, G, Mora-Gil, J, Llanes, N and Villalba, D. Nutrient utilization efficiency, ruminal fermentation and microbial community in Holstein bulls fed concentrate-based diets with different forage source. *Anim. Feed Sci. Technol.* 2020;269:114662. <https://doi.org/10.1016/j.anifeedsci.2020.114662>

Cunha, CS, Veloso, CM, Marcondes, MI, Mantovani, HC, Tomich, TR, Pereira, LGR, et al. Assessing the impact of rumen microbial communities on methane emissions and production traits in Holstein cows in a tropical climate. *Sys. Appl. Microbiol.* 2017;40:8:492-499. <https://doi.org/10.1016/j.syapm.2017.07.008>

Cunha, CS, Marcondes, MI, Veloso, CM, Mantovani, HC, Pereira, LGR, Tomich, TR, Dill-McFarland, KA and Suen, G. Compositional and structural dynamics of the ruminal microbiota in dairy heifers and its relationship to methane production. *Sci. Food Agri.* 2018;99:1:210-218. <https://doi.org/10.1002/jsfa.9162>

Czerkawski, JW, Blaxter, KL and Wainman, FW. The metabolism of oleic, linoleic, and linolenic acids by sheep with reference to their effects on methane production. *Br. J. Nutri.* 1966;20:349. <https://doi.org/10.1079/BJN19660035>

de Bok, FAM, Plugge, CM and Stams, AJM. Interspecies electron transfer in methanogenic propionate degrading consortia. *Water Res.* 2004;38:1368-1375. <https://doi.org/10.1016/j.watres.2003.11.028>

Decaestecker, E, De Gersem, H, Michalakis, Y and Raeymaekers, JAM. Damped long-term host-parasite Red Queen coevolutionary dynamics: a reflection of dilution effects? *Ecol. Lett.* 2013;16:1455-1462. <https://doi.org/10.1111/ele.12186>

Demirel, B and Scherer, P. The roles of acetotrophic and hydrogenotrophic methanogens during anaerobic conversion of biomass to methane: a review. *Rev. Environ. Sci. Technol*. 2008;7:173-190. <https://doi.org/10.1007/s11157-008-9131-1>

Deng, L, He, C, Zhou, Y, Xu, L and Xiong, H. Ground transport stress affects bacteria in the rumen of beef cattle: A real-time PCR analysis. *Ani. Sci. J.* 2017;88:790-797. <https://doi.org/10.1111/asj.12615>

Dhanasekaran, DK, Dias-Silva, TP, Filho, ALA, Sakita, GZ, Abdalla, AL, Louvandini, H and Elghandour, MMMY. Plants extract and bioactive compounds on rumen methanogenesis. *Agroforest. Syst.* 2020;94:1541-1553. <https://doi.org/10.1007/s10457-019-00411-6>

Díaz Carrasco, J.M., Cabral, C., Redondo, L.M., Pin Viso, N.D., Colombatto, D., Farber, M.D. and Miyakawa, M.E.F. 2017. Research article: Impact of chestnut and quebracho tannins on rumen microbiota of bovines. *Hindawi BioMed Res. Internatl.* 2017:9610810. <https://doi.org/10.1155/2017/9610810>

Doetsch, RN, Robinson, RQ, Brown, RE and Shaw, JC. Catabolic reactions of mixed suspensions of bovine rumen bacteria. *J. Dairy Sci.* 1953;36:825-831. <https://doi.org/10.3168/jds.S0022-0302(53)91568-9>

Downing, AL, Brown, BL and Leibold, MA. Multiple diversity-stability mechanisms enhance population and community stability in aquatic food webs. *Ecology*. 2014;95:173-184. <https://doi.org/10.1890/12-1406.1>

Duin, EC, Wagner, T, Shima, S, Prakash, D, Cronin, B, Yáñez-Ruiz, DR, et al. Mode of action uncovered for the specific reduction of methane emissions from ruminants by the small molecule 3-nitrooxypropanol. *Proc. Natl. Acad. Sci. USA.* 2016;113:22:6172-6177. <https://doi.org/10.1073/pnas.1600298113>

Edenhofer, O. Climate Change 2014: Mitigation of Climate Change; Cambridge University Press. Cambridge, MA, USA. 2015. Vol. 3.

Edwards, JE, McEwan, NR, Travis, AJ and Wallace, RJ. 16S rDNA library-based analysis of ruminal bacterial diversity. *Antonie van Leeuwenhoek*. 2004;86:263-281. <https://doi.org/10.1023/B:ANTO.0000047942.69033.24>

Elekwachi, CO, Wang, Z, Wu, X, Rabee, A and Forster, RJ. Total rRNA-seq analysis gives insight into bacterial, fungal, protozoal and archaeal communities in the rumen using an optimized RNA isolation method.  *Front. Microbiol*. 2017;8:1814. <https://doi.org/10.3389/fmicb.2017.01814>

Enjalbert, F, Combes, S, Zened, A and Meynadier, A. Rumen microbiota and dietary fat: a mutual shaping. *J. Appl. Microbiol.* 2017;123 :782-797. <https://doi.org/10.1111/jam.13501>

Eugène, M, Massè, D, Chiquette, J and Benchaar, C. Meta-analysis of lipid supplementation on methane production in lactating dairy cows. *Can. J. Anim. Sci.* 2008 ;88:331-334. <https://doi.org/10.4141/CJAS07112>

Faixova, Z and Faix, S. Manipulation of rumen nitrogen metabolism – a review. *Folia Vet.* 2005;49:215-219.

Fenchel, T, King, GM and Blackburn, TH. Bacterial Metabolism. In: Fenchel T, King GM, Blackburn THBT, editors. Bacterial biogeochemistry. 3rd ed. Boston: Academic Press; 2012. p. 1–34. <https://doi.org/10.1016/B978-0-​12-​415836-​8.00001-3>

Fargione, JE and Tilman, D. Diversity decreases invasion via both sampling and complementary effects. *Ecol. Lett*. 2005;8:604-611. <https://doi.org/10.1111/j.1461-0248.2005.00753.x>

Fernando, SC, Purvis, HT, Najar, FZ, Sukharnikov, LO, Krehbiel, CR, Nagaraja, TG, Roe, BA and DeSilva, U. Rumen microbial population dynamics during adaptation to a high grain diet. *Appl. Environ. Microbiol*. 2010;76:22:7482-7490. <https://doi.org/10.1128/AEM.00388-10>

Ferry, JG. Enzymology of one-carbon metabolism in methanogenic pathways. *FEMS Microbiol*. *Rev.* 1999;23:13-38. <https://doi.org/10.1111/j.1574-6976.1999.tb00390.x>

Finn, D, Ouwerkerk, D and Klieve, A. Methanotrophs from natural ecosystems as biocontrol agents for ruminant methane. 2012. Final Report for Meat & Livestock Australia Limited. Sydney, Australia. <https://era.daf.qld.gov.au/id/eprint/2290>

Firkins, JL. Reconsidering rumen microbial consortia to enhance feed efficiency and reduce environmental impact of ruminant livestock production systems. *Rev. Bras. Zootec*. 2010;39:445-457. <https://doi.org/10.1590/S1516-35982010001300049>

Foley, PA, Kenny, DA, Callan, JJ, Boland, TM and O’Hara, FP. Effect of dl-malic acid supplementation on feed intake, methane emission, and rumen fermentation in beef cattle. *J. Anim. Sci.* 2009;87:1048-1057. <https://doi.org/10.2527/jas.2008-1026>

Fonty, G, Gouet, P, Jouany, JP and Senaud, J. Establishment of the microflora and anaerobic fungi in the rumen of lambs. *J. Gen. Microbiol*. 1987;133:1835-1843. <https://doi.org/10.1099/00221287-133-7-1835>

Franz, C, Baser, KHC and Windisch, W. Essential oils and aromatic plants in animal feeding – a European perspective. A review. *Flavour Fragr. J.* 2010;25:327-340. <https://doi.org/10.1002/ffj.1967>

Frazier, AN. Application of biotechnology in agriculture: A bioinformatic and market analysis of novel intervention methods. Doctoral Dissertation. 2021. Colorado State University.

Friedman, N, Shriker, E and Gold, B. Diet-induced changes of redox potential underlie compositional shifts in the rumen archaeal community. *Environ*. *Microbiol*. 2017;19:174-184. <https://doi.org/10.1111/1462-2920.13551>

Furman, O, Shenhav, L, Sasson, G, Kokou, F, Honig, H, Jacoby, S, et al. Stochasticity constrained by deterministic effects of diet and age drive rumen microbiome assembly dynamics. *Nat. Com*. 2020;11:1904. <https://doi.org/10.1038/s41467-020-15652-8>

Gerber, P, Vellinga, T, Opio, C and Steinfeld, H. Productivity gains and greenhouse gas emissions intensity in dairy systems. *Livest. Sci.* 2011;139:100-108. <https://doi.org/10.1016/j.livsci.2011.03.012>

Gislon, G, Colombini, S, Borreani, G, Crovetto, GM, Sandrucci, A, Galassi, G, Tabacco, E and Rapetti, L. Milk production, methane emissions, nitrogen, and energy balance of cows fed diets based on different forage systems. *J. Dairy Sci.* 2020;103:8048-8061. <https://doi.org/10.3168/jds.2019-18134>

Givens, DJ and Shingfield, KJ. Optimizing dairy milk fatty acid composition. Improving fat content of foods. 2006;252-280.

Glasson, CRK, Kinley, RD, de Nys, R, King, N, Adams, SL, Packer, MA, et al. Benefits and risks of including the bromoform containing seaweed *Asparagopis* in feed for the reduction of methane production from ruminants. *Algal Res.* 2022;64:102673. <https://doi.org/10.1016/j.algal.2022.102673>

Goel, G, Makkar, HP and Becker, K. Effects of *Sesbani a sesban* and *Carduus pycnocephalus* leaves and Fenugreek (*Trigonella foenum-graecum L.*) seeds and their extracts on partitioning of nutrients from roughage- and concentrate-based feeds to methane. *Anim. Feed Sci. Technol*. 2008;147:72-89. <https://doi.org/10.1016/j.anifeedsci.2007.09.010>

González, LA, Manteca, X, Calsamiglia, S, Schwartzkopf-Genswein, KS and Ferret, A. Ruminal acidosis in feedlot cattle: interplay between feed ingredients, rumen fermentation and feeding behavior (a review). *Anim. Feed Sci. Technol*. 2012.172. 66-79. <https://doi.org/10.1016/j.anifeedsci.2011.12.009>

Gould, DH. Polioencephalomalacia. *J. Anim. Sci.* 1998;76:1:309-314. <https://doi.org/10.2527/1998.761309x>

Grainger, C, Clarke, T, Beauchemin, KA, McGinn, SM and Eckard, RJ. Supplementation with whole cottonseed reduces methane emissions and can profitably increase milk production of dairy cows offered a forage and cereal grain diet. *Aust. J. Experi. Agricul.* 2008;48:2:73-76. <https://doi.org/10.1071/EA07224>

Grainger, C and Beauchemin, KA. Can enteric methane emissions from ruminants be lowered without lowering their production? *Anim. Feed Sci. Technol.* 2011;166-167:308-320. <https://doi.org/10.1016/j.anifeedsci.2011.04.021>

Greening, C, Geier, R, Wang, C, Woods, LC, Morales, SE, McDonald, MJ, et al. Diverse hydrogen production and consumption pathways influence methane production in ruminants. *ISME J.* 2019;13:2617-2632. <https://doi.org/10.1038/s41396-019-0464-2>

Gruby, D and Delafond, HMO. Recherches ser des animalcules se de veloppant en grand nombre dans I’estomac et dans les intestins, pedant la digestion des animaux herbivores et carnivores. *Compt. Rend. Acad. Sci.* 1843;17 :1304-1308.

Gruninger, RJ, Zhang, XM, Smith, ML, Kung Jr., L, Vyas, D, McGinn, SM, et al. Application of 3-nitrooxypropanol and canola oil to mitigate enteric methane emissions of beef cattle results in distinctly different effects on the rumen microbial community. *Anim. Microbiome.* 2022 ;4:35. <https://doi.org/10.1186/s42523-022-00179-8>

Guerrero-Cruz, S, Vaksmaa, A, Horn, MA, Niemann, H, Pijuan, M and Ho, A. Methanotrophs: Discoveries, environmental relevance, and a future perspective on current and future applications. 2021;12:678057. <https://doi.org/10.3389/fmicb.2021.678057>

Guyader, J, Eugène, M, Nozière, P, Morgavi, D, Doreau, M, & Martin, C. Influence of rumen protozoa on methane emission in ruminants: A meta-analysis approach. *Animal*. 2014;8:11:1816-1825. <https://doi.org/10.1017/S1751731114001852>

Guyader, J, Janzen, HH, Kroebel, R and Beauchemin, KA. Forage utilization to improve environmental sustainability of ruminant production. *J. Anim. Sci.* 2016;94:3147-3158. <https://doi.org/10.2527/jas.2015-0141>

Guzman, CE, Bereza-Malcolm, LT, De Groef, B and Franks, AE. Presence of selected methanogens, fibrolytic bacteria, and proteobacteria in the gastrointestinal tract of neonatal dairy calves from birth to 72 hours. *PLoS ONE*. 2015;10:e0133048. <https://doi.org/10.1371/journal.pone.0133048>

Haisan, J, Sun, Y, Guan, L, Beauchemin, KA, Iwaasa, A, Duval, S, et al. The effects of feeding 3-nitrooxypropanol at two doses on milk production, rumen fermentation, plasma metabolites, nutrient digestibility, and methane emissions in lactating Holstein cows. *Anim. Prod. Sci*. 2017 ;57:282-289. <https://doi.org/10.1071/AN15219>

Hanson, RS and Hanson, TE. Methanotrophic bacteria. Microbiol. Rev. 1996;60:439-471. <https://doi.org/10.1128/mmbr.60.2.439-471.1996>

Hart, EH, Creevey, CJ, Hitch, T and Kingston-Smith, AH. Meta-proteomics of rumen microbiota indicates niche compartmentalization and functional dominance in limited number of metabolic pathways between abundant bacteria. *Sci. Rep*. 2018;8:10504. <https://doi.org/10.1038/s41598-018-28827-7>

Hassan, F, Arshad, MA, Ebeid, HM, Rehman, MS, Khan, MS, Shahid, S and Yang, C. Phytogenic additives can modulate rumen microbiome to mediate fermentation kinetics and methanogenesis through exploiting diet-microbe interaction. *Front. Vet. Sci*. 2020;7:575801. <https://doi.org/10.3389/fvets.2020.575801>

Hassanat, F, Gervais, R, Julien, C, Massé, DI, Lettat, A, Chouinard, PY, Petit, HV and Benchaar, C. Replacing alfalfa silage with corn silage in dairy cow diets: Effects on enteric methane production, ruminal fermentation, digestion, N balance, and milk production. *J. Dairy Sci*. 2013;96:4553-4567. <https://dx.doi.org/10.3168/jds.2012-6480>

He, L, Chen, N, Lv, H, Wang, C, Zhou, W, Chen, X and Zhang, Q. Gallic acid influencing fermentation quality, nitrogen distribution, and bacterial community of high-moisture mulberry leaves and stylo silage. *Bioresource Technol.* 2020;295:122255. <https://doi.org/10.1016/j.biortech.2019.122255>

Hegarty, RS. Reducing rumen methane emissions through elimination of rumen protozoa. *Aust. J. Agric. Res.* 1999;50:1321. <https://doi.org/10.1071/AR99008>

Hegarty, RS, Bird, SH, Vanselow, BA and Woodgate, R. Effects of the absence of protozoa from birth or from weaning on the growth and methane production in lambs. *Br. J. Nutr.* 2008;100:1220-1227. <https://doi.org/10.1017/S0007114508981435>

Hegarty, RS, Cortez Passetti, RA, Dittmer, KM, Wang, Y, Beauchemin, SK, Emet-Booth, J, et al. An evaluation of emerging feed additives to reduce methane emissions from livestock. Edition 1. A report coordinated by Climate Change, Agriculture and Food Security (CCAFS) and the New Zealand Agriculture Greenhouse Gas Research Centre (NZAGRC) initiative of the Global Research Alliance (GRA). 2021. <https://cgspace.cgiar.org/handle/10568/116489>

Henderson, C. The influence of extracellular hydrogen on the metabolism of *Bacteroides ruminicola*, *Anaerovibrio lipolytica*, and *Selenomonas ruminantium*. *Microbiol*. 1980;119:485-491. <https://doi.org/10.1099/00221287-119-2-485>

Henderson, G, Cox, F, Ganesh, S, Jonker, A, Young, W, Global Rumen Census Collaborators and Janssen, PH. Rumen microbial community composition varies with diet and host, but a core microbiome is found across a wide geographical range. *Nat. Sci. Rep.* 2015;5:14567. <https://doi.org/10.1038/srep14567>

Henniger, MT, Wells, JE, Hales, KE, Lindholm-Perry, AK, Freetly, HC, Kuehn, LA, et al. Effects of a moderate or aggressive implant strategy on the rumen microbiome and metabolome in steers. *Front. Anim. Sci.* 2022;3:889817. <https://doi.org/10.3389/fanim.2022.889817>

Herd, RM, Arthur, PF, Donoghue, KA, Bird, SH, Bird-Gardiner, T and Hegarty, RS. Measures of methane production and their phenotypic relationships with dry matter intake, growth, and body composition traits in beef cattle. *J. Anim. Sci*. 2014;92:5267-5274. <https://doi.org/10.2527/jas.2014-8273>

Hobson, PN and Stewart, CS. The Rumen Microbial Ecosystem. Blackie Academic & Professional. 2012. New York, USA.

Hofmann, RR. Evolutionary steps of ecophysiological adaption and diversification of ruminants: a comparative view of their digestive system. *Oecologia*. 1989;78:443-457. <https://doi.org/10.1007/BF00378733>

Hooper, DU, Hawksworth, D and Dhillion, S. Microbial diversity and ecosystem processes. Global biodiversity assessment *UNEP*. Cambridge University Press. Cambridge. 1995;433-443.

Hristov, AN, Oh, J, Firkins, JL, Dijkstra, J, Kebreab, E, Waghorn, G, et al. Mitigation of methane and nitrous oxide emissions from animal operations: A review of enteric methane mitigation options. *J. Anim. Sci.* 2013;91:5045-5069. <https://doi.org/10.2527/jas.2013-6583>

Hristov, A, Oh, J, Giallongo, F, Frederick, TW, Harper, MT, Weeks, HL, et al. An inhibitor persistently decreased enteric methane emission from dairy cows with no negative effect on milk production. *Proc. Natl. Acad. Sci.* *USA*. 2015;112:10663-10668. <https://doi.org/10.1073/pnas.1504124112>

Hristov, AN, Melgar, A, Wasson, D and Arndt, C. Symposium review: Effective nutritional strategies to mitigate enteric methane in dairy cattle. *J. Dairy Sci*. 2022;105:8543-8557. <https://doi.org/10.3168/jds.2021-21398>

Hu, X, Li, S, Mu, R, Guo, J, Zhao, C, Cao, Y, Zhang, N and Fu, Y. The rumen microbiota contributes to the development of Mastitis in dairy cows. *Microbiol. Spect.* 2022;10:1:e02512-02521. <https://doi.org/10.1128/spectrum.02512-21>

Hua, C, Tian, J, Tian, P, Cong, R, Luo, Y, Geng, Y, et al. Feeding a high concentration diet induces unhealthy alterations in the composition and metabolism of ruminal microbiota and host response in a goat model. *Front. Microbiol.* 2017;8:138. <https://doi.org/10.3389/fmicb.2017.00138>

Huws, SA, Creevey, CJ, Oyama, LB, Mizrahi, I, Denman, SE, Popova, M, et al. Addressing global ruminant agricultural challenges through understanding the rumen microbiome: past, present and future. *Front. Microbiol*. 2018;9:2161. <https://doi.org/10.3389/fmicb.2018.02161>

Iqbal, MW, Zhang, Q, Yang, Y, Li, L, Zou, C, Huang, C and Lin, B. Comparative study of rumen fermentation and microbial community differences between water buffalo and Jersey cows under similar feeding conditions. *J. Appl. Ani. Res.* 2018;46:1:740-748. <https://doi.org/10.1080/09712119.2017.1394859>

Iwamoto, M, Asanuma, N and Hino, T. *Selenomonas ruminantium*, *Veillonella parvula*, and *Wolinella succinogenes* to reduce nitrate and nitrite with special reference to the suppression of ruminal methanogenesis. *Anaerobe*. 2002;8:209-215.

Jami, E and Mizrahi, I. Composition and similarity of bovine rumen microbiota across individual animals. *PLoS One*. 2012;7:e33306. <https://doi.org/10.1371/journal.pone.0033306>

Janssen, PH and Kirs, M. Structure of the archaeal community of the rumen. *Appl. Environ. Microbiol*. 2008;74 :3619-3625. <https://doi.org/10.1128/AEM.02812-07>

Janssen, PH. Influence of hydrogen on rumen methane formation and fermentation balances through microbial growth kinetics and fermentation thermodynamics. *Anim. Feed Sci. Technol*. 2010;160:1-22. <https://doi.org/10.1016/j.anifeedsci.2010.07.002>

Jarvis, GN and Moore, ER. Lipid metabolism and the rumen microbial ecosystem. Handbook of Hydrocarbon and Lipid Microbiology, Springer. 2010:2245-2257.

Jeyanathan, J, Martin, C and Morgavi, DP. The use of direct-fed microbials for mitigation of ruminant methane emissions: a review. Animal. 2014;8:2:250-261. <https://doi.org/10.1017/S1751731113002085>

Jeyanathan, J, Martin, C and Morgavi, DP. Screening of bacterial direct-fed microbials for their antimethanogenic potential *in vitro* and assessment of their effect on ruminal fermentation and microbial profiles in sheep. *J. Anim. Sci*. 2016;94:739-750. <https://doi.org/10.2527/jas2015-9682>

Johnson, KA and Johnson, DE. Methane emissions from cattle. *J. Anim. Sci.* 1995;73:2483-2492. <https://doi.org/10.2527/1995.7382483x>

Kajikawa, H, Valdes, C, Hillman, K, Wallace, RJ and Newbold, CJ. Methane oxidation and its coupled electron-sink reactions in ruminal fluid. *Lett. Appl. Microbiol.* 2003;36:354-357. <https://doi.org/10.1046/j.1472-765X.2003.01317.x>

Karkman, A, Lehtimäki, J and Ruokolainen, L. Th ecology of human microbiota: dynamics and diversity in health and disease. *Ann. N.Y. Acad. Sci.* 2017;1399:78-92. <https://doi.org/10.1111/nyas.13326>

Kelly, WJ, Mackie, RI, Attwood, GT, Janssen, PH, McAllister, TA and Leahy, SC. Hydrogen and formate production and utilization in the rumen and the human colon. *Anim. Microbiome*. 2022;4:22. <https://doi.org/10.1186/s42523-022-00174-z>

Khafipour, E, Li, S, Plaizier, JC, Dowd, SE and Krause, DO. Microbiome analysis of the rumen, cecum, and feces of dairy cows with subacute ruminal acidosis. *J. Anim. Sci.* 2011;89:489.

Kim, E-T, Lee, S-J, Kim, T-Y, Lee, H-G, Atikur, RM, Gu, B-H, et al,. Dynamic changes in fecal microbial communities of neonatal dairy calves by aging and diarrhea. *Animals*. 2021;11:1113. <https://doi.org/10.3390/ani11041113>

Kittelmann, S, Pinares-Patiño, CS, Seedorf, H, Kirk, MR, Ganesh, S, McEwan, JC and Janssen, PH. Two different bacterial community types are linked with the low-methane emission trait in sheep. *PLoS One*. 2014;9:7:e103171. <https://doi.org/10.1371/journal.pone.0103171>

Knapp, JR, Laur, GL, Vadas, PA, Weiss, WP and Tricarico, JM. Invited review: Enteric methane in dairy cattle production: Quantifying the opportunities and impact of reducing emissions. *J. Dairy Sci*. 2014;97:3231-3261. <https://doi.org/10.3168/jds.2013-7234>

Kobayashi, Y. Abatement of methane production from ruminants: trends in the manipulation of rumen fermentation. *Asian Austral. J. Anim. Sci.* 2010;23:410-416. <https://doi.org/10.5713/ajas.2010.r.01>

Koskella, B, Hall, LJ and Metcalf, JE. The microbiome beyond the horizon of ecological and evolutionary theory. *Nat. Ecol. Evol.* 2017;1:1606-1615. <https://doi.org/10.1038/s41559-017-0340-2>

Kurade, MB, Saha, S, Salama, E-S, Patil, SM, Govindwar, SP and Jeon, B-H. Acetoclastic methanogenesis led by *Methanosarcina* in anaerobic co-digestion of fats, oil and grease for enhanced production of methane. *Biores. Technol*. 2019;272:351-359. <https://doi.org/10.1016/j.biortech.2018.10.047>

Lan, W and Yang, C. Ruminal methane production: Associated microorganisms and the potential of applying hydrogen-utilizing bacteria for mitigation. *Sci. Total Environ.* 2019;654:1270-1283. <https://doi.org/10.1016/j.scitotenv.2018.11.180>

Latham, EA, Anderson, RC, Pinchak, WE and Nisbet, DJ. Insights on alterations to the rumen ecosystem by nitrate and nitrocompounds. *Front. Microbiol*. 2016;7:228. <https://doi.org/10.3389/fmicb.2016.00228>

Leng, RA. Interactions between microbial consortia in biofilms: a paradigm shift in rumen microbial ecology and enteric methane mitigation*. Anim. Prod. Sci.* 2014;54:519-543. <https://doi.org/10.1071/AN13381>

Li, RW, Connor, EE, Li, C, Baldwin, RL and Sparks, ME. Characterization of the rumen microbiota of pre-ruminant calves using metagenomic tools. *Environ*. *Microbiol*. 2012;14:129-139.  <https://doi.org/10.1111/j.1462-2920.2011.02543.x>

Li, F, Li, C, Chen, Y, Liu, J, Zhang, C, Irving, B, et al. Host genetics influence the rumen microbiota and heritable rumen microbial features associate with feed efficiency in cattle. *Microbiome*. 2019;7:92. <https://doi.org/10.1186/s40168-019-0699-1>

Li, YQ, Xi, YM, Wang, ZD, Zeng, HF and Han, Z. Combined signature of rumen microbiome and metabolome in dairy cows with different feed intake levels. *J. Anim. Sci.* 2020;98:3:1-15. <https://doi.org/10.1093/jas/skaa070>

Llonch, P, Somarriba, M, Duthie, C-A, Haskell, MJ, Rooke, JA, Troy, S, et al. Association of temperament and acute stress responsiveness with productivity, feed efficiency, and methane emissions in beef cattle: An observational study. *Front. Vet. Sci.* 2016;3:43. <https://doi.org/10.3389/fvets.2016.00043>

Loreau, M, Mouquet, N and Gonzalez, A. Biodiversity as spatial insurance in heterogeneous landscapes. *Proc. Natl. Acad. Sci. U.S.A.* 2003;100:12765-12770. <https://doi.org/10.1073/pnas.2235465100>

Louis, P, Scott, KP Duncan, SH and Flint, HJ. Understanding the effects of diet on bacterial metabolism in the large intestine. *J. Appl. Microbiol.* 2007;102:1197-1208. <https://doi.org/10.1111/j.1365-2672.2007.03322.x>

Lourenço, M, Ramos-Morales, E and Wallace, RJ. The role of microbes in rumen lipolysis and biohydrogenation and their manipulation. *Animal*. 2010;4:7:1008-1023. <https://doi.org/10.1017/S175173111000042X>

Lyu, Z, Shao, N, Akinyemi, T and Whitman, WB. Methanogenesis. *Curr. Biol.* 2018;28:13:R727-R732. <https://www.cell.com/current-biology/pdf/S0960-9822(18)30623-7.pdf>

Ma, T, Villot, C, Renaud, D, Skidmore, A, Chevaux, E, Steele, M and Guan, LL. Linking perturbations to temporal changes in diversity, stability, and compositions of neonatal calf gut microbiota: prediction of diarrhea. *ISME. J.* 2020;14:2223-2235. <https://doi.org/10.1038/s41396-020-0678-3>

Maia, MRG, Chaudhary, LC, Figueres, L and Wallace, RJ. Metabolism of polysaturated fatty acids and their toxicity to the microflora of the rumen. *Ant. Van. Leeuw.* 2007;91:303-314. <https://doi.org/10.1007/s10482-006-9118-2>

Maman, LG, Palizban, F, Atanaki, FF, Ghiasi, NE, Ariaeenejad, S, Ghaffari, MR, et al. Co-abundance analysis reveals hidden players associated with high methane yield phenotype in sheep rumen microbiome. *Nat. Sci. Rep*. 2020;10:4995. <https://doi.org/10.1038/s41598-020-61942-y>

Mamuad, L, Kim, SH, Jeong, CD, Choi, YJ, Jeon, CO and Lee, S-S. Effect of fumarate reducing bacteria on *in vitro* rumen fermentation, methane mitigation, and microbial diversity. *J. Microbiol*. 2014;52:120-128. <https://doi.org/10.1007/s12275-014-3518-1>

Mao, H-L, Wang, J-K, Zhou, Y-Y and Liu, J-X. Effects of addition of tea saponins and soybean oil on methane production, fermentation and microbial population in the rumen of growing lambs. 2010;129:56-62. <https://doi.org/10.1016/j.livsci.2009.12.011>

Martin, SA and Park, C-M. Effect of extracellular hydrogen on organic acid utilization by the ruminal bacterium *Selenomonas ruminantium*. *Curr. Microbiol*. 1996:32;327-331. <https://doi.org/10.1007/s002849900058>

Martin, C, Ferlay, A, Mosoni, P, Rochette, Y, Chilliard, Y and Doreau, M. Increasing linseed supply in dairy cow diets based on hay or corn silage: effect on enteric methane emission, rumen microbial fermentation, and digestion. *J. Dairy Sci.* 2016;99:3445-3456. <https://doi.org/10.3168/jds.2015-10110>

Martinez-Fernandez, G, Duval, S, Kindermann, M, Schirra, HJ, Denman, SE and McSweeney, CS. 3-NOP vs. halogenated compound: Methane production, ruminal fermentation and microbial community response in forage fed cattle. *Front. Microbiol*. 2018;9:1582. <https://doi.org/10.3389/fmicb.2018.01582>

McAllister, TA, Bae, HD, Jones, GA and Cheng, KJ. Microbial attachment and feed digestion in the rumen. *J. Anim. Sci.* 1994;72:3004-3018. <https://doi.org/10.2527/1994.72113004x>

McAllister, TA and Newbold, CJ. Redirecting rumen fermentation to reduce methanogenesis. *Aust. J. Exp. Agricul.* 2008;48:7-13. <https://doi.org/10.1071/EA07218>

Meale, SJ, Li, S, Azevedo, P, Derakhshani, H, Plaizier, JC, Khafipour, E and Steele, MA. Development of ruminal and fecal microbiomes are affected by weaning but not weaning strategy in dairy calves. *Front. Microbiol.* 2016;7:582. <https://doi.org/10.3389/fmicb.2016.00582>

Meale, SJ, Li, SC, Azevedo, P, Derakhshani, H, DeVries, TJ, Plaizier, JC, Steele, MA and Khafipour, E. Weaning age influences the severity of gastrointestinal microbiome shifts in dairy calves. *Nat. Sci. Rep.* 2017;7:198. <https://doi.org/10.1038/s41598-017-00223-7>

Meale, SJ, Popova, M, Saro, C, Martin, C, Bernard, A, Lagree, M, et al. Early life dietary intervention in dairy calves results in a long-term reduction in methane emissions. *Sci. Rep. Nat.* 2021;11:3003. <https://doi.org/10.1038/s41598-021-82084-9>

Min, BR, Parker, D, Brauer, D, Waldrip, H, Lockard, C, Hales, K, et al. The role of seaweed as a potential dietary supplementation for enteric methane mitigation in ruminants: Challenges and opportunities. *Anim. Nutr.* 2021;7:1371-1387. <https://doi.org/10.1016/j.aninu.2021.10.003>

Mitsumori, M, Ajisaka, N, Tajima, K, Kajikawa, H and Kurihara, M. Detection of Proteobacteria from the rumen by PCR using methanotroph-specific primers. Let. Appl. Microbiol. 2002;35:3:251-255. <https://doi.org/10.1046/j.1472-765X.2002.01172.x>

Mizrahi, I. Rumen Symbioses. In *The Prokaryotes*. Eds. Rosenberg, E., DeLong, E.F., Lory, S., Stackebrandt, E. and Thompson, F. Springer-Verlag Berlin Heidelberg. 2013;533-544. <https://doi.org/10.1007/978-3-642-30194-0>

Mizrahi, I, Wallace, RJ and Moraïs, S. The rumen microbiome: balancing food security and environmental impacts. *Nat. Rev*. 2021;19. <https://doi.org/10.1038/s41579-021-00543-6>

Moraïs, S and Mizrahi, I. The road not taken: the rumen microbiome, functional groups, and community states. *Trends Microbiol*. 2019a;27:6. <https://doi.org/10.1016/j.tim.2018.12.011>

Moraïs, S and Mizrahi, I. Islands in the stream: from individual to communal fiber degradation in the rumen ecosystem. *FEMS Rev. Microbiol*. 2019b;43:362-379. <https://doi.org/10.1093/femsre/fuz007>

Morgavi, DP, Jounay, JP and Martin, C. Changes in methane emission and rumen fermentation parameters induced by refaunation in sheep. *Aust. J. Exp. Agric.* 2008;48:69-72. <https://doi.org/10.1071/EA07236>

Morgavi, DP, Forano, E, Martin, C and Newbold, CJ. Microbial ecosystem and methanogenesis in ruminants. *Animal*. 2010;4:1024-1036. <https://doi.org/10.1017/S1751731110000546>

Morgavi, DP, Martin, C, Jouany, J-P, and Ranilla, MJ. Rumen protozoa and methanogenesis: not a simple cause-effect relationship. *Br. J. Nutr.* 2012;107:388-397. <https://doi.org/10.1017/S0007114511002935>

Morvan, B, Doré, J, Rieu-Lesme, F, Foucat, L, Fonty, G and Gouet, P. Establishment of hydrogen-utilizing bacteria in the rumen of the newborn lamb. *FEMS Microbiol*. *Lett*. 1994 ;117:249-256. <https://doi.org/10.1111/j.1574-6968.1994.tb06775.x>

Neu, AT, Allen, EE and Roy, K. Defining and quantifying the core microbiome: challenges and prospects. *Proc. Natl. Acad. Sci. U.S.A*. 2021;118:e2104429118. <https://doi.org/10.1073/pnas.2104429118>

Newbold, CJ, de la Fuente, G, Belanche, A, Ramos-Morales, E and McEwan, NR. The role of ciliate protozoa in the rumen. *Front. Microbiol*. 2015;6:1313. <https://doi.org/10.3389/fmicb.2015.01313>

Newbold, CJ and Ramos-Morales, E. Review: Ruminal microbiome and microbial metabolome: effects of diet and ruminant host. *Animal*. 2020;14:S1:s78-s86. <https://doi.org/10.1017/S1751731119003252>

New Zealand Agricultural Greenhouse Gas Research Centre (NZAGRC). Annual Report. 2019;51-59. <https://www.nzagrc.org.nz/annualreport,listing,598,annual-report-2019.html>

O’Hara, E, Kenny, DA, McGovern, E, Byrne, CJ, McCabe, MS, Guan, LL and Waters, SM. Investigating the temporal microbial dynamics in the rumen of beef calves raised on two farms during early life. *FEMS Mirobiol. Ecol*. 2020;96:fiz203. <https://doi.org/10.1093/femsec/fiz203>

O’Hara, E, Terry, SA, Moote, P, Beauchemin, KA, McAllister, TA, Abbott, DW and Gruninger, RJ. Comparative analysis of macroalgae supplementation on the rumen microbial community: *Asparagopsis taxiformis* inhibits major ruminal methanogenic, fibrolytic, and volatile fatty acid-producing microbes *in vitro*. *Front. Microbiol*. 2023;14:1104667. <https://doi.org/10.3389/fmicb.2023.1104667>

Oltjen, JW. How did Lofgreen and Garrett do the math? *Transl. Anim. Sci.* 2019;3:1011-1017. <https://doi.org/10.1093/tas/txz072>

Ørskov, ER, Ojwang, I and Reid, GW. A study in consistency of differences between cows in rumen outflow rate of fibrous particles and other substrates and consequences for digestibility and intake of roughages. *Anim. Sci.* 1988;47:45-51. <https://doi.org/10.1017/S000335610003703X>

Paillard, D, McKain, N, Chaudary, LC, Walker, ND, Pizette, F, Koppova, I, et al. Relation between phylogenetic position, lipid metabolism and butyrate production by different *Butyrivibrio*-like bacteria from the rumen. *Ant. Van Leeuw.* 2007;91:417-422. <https://doi.org/10.1007/s10482-006-9121-7>

Parr, SL. "Anabolic Implant Strategies in Beef Production", in: American Association of Bovine Practitioners Proceedings of the Annual Conference) 2020;20–24. <https://doi.org/10.21423/aabppro20207957>

48. Paul SS, Bu D, Xu J, Hyde KD and Yu Z. A phylogenetic census of global diversity of gut anaerobic fungi and a new taxonomic framework. *Fung. Divers.* 2018;89:253-266. <https://doi.org/10.1007/s13225-018-0396-6>

Pereira, A.M., de Lurdes Nunes Enes Dapekevicius, M. and Borba, A.E.S. Alternative pathways for hydrogen sink originated from the ruminal fermentation of carbohydrates: Which microorganisms are involved in lowering methane emission? *Anim. Microbiome*. 2022;4:1:5. <https://doi.org/10.1186/s42523-021-00153-w>

Pitta, DW, Melgar, A, Hristov, AN, Indugu, N, Narayan, KS, Pappalardo, C, et al. Temporal changes in total and metabolically active ruminal methanogens in dairy cows supplemented with 3-nitrooxypropanol. *J. Dairy. Sci*. 2021;104:8:8721-8735. <https://doi.org/10.3168/jds.2020-19862>

Pitta, DW, Indugu, N, Melgar, A, Hristov, A, Challa, K, Vecchiarelli, B, et al. The effect of 3-nitrooxypropanol, a potent methane inhibitor, on ruminal microbial gene expression profiles in dairy cows. *Microbiome*. 2022;10:146. <https://doi.org/10.1186/s40168-022-01341-9>

Pope, PB, Smith, W, Denman, SE, Tringe, SG, Bary, K, Hugenholtz, P, et al. Isolation of *Succinivibrionaceae* implicated in low methane emissions from Tammar wallabies. *Sci*. 2011;333:646-648. <https://doi.org10.1126/science.1205760>

Rabiee, AR, Breinhild, K, Scott, W, Golder, HM, Block, E and Jean, IJ. Effect of fat additions to diets of dairy cattle on milk production and components: A meta-analysis and meta-regression. *J. Dairy Sci.* 2012;95:3225-3247. <https://doi.org/10.3168/jds.2011-4895>

Ramayo-Caldas, Y, Zingaretti, L, Popova, M, Estellé, J, Bernard, A, Pons, N, et al. Identification of rumen microbial biomarkers linked to methane emission in Holstein dairy cows. *J. Anim. Breed Genet.* 2020;137:49-59. <https://doi.org/10.1111/jbg.12427>

Rani, A, Pundir, A, Verma, M, Joshi, S, Verma, G, Andjelković, S, Babić, S, Milenković and Mitra, D. Methanotrophy: a biological method to mitigate global methane emission. Microbiol. Res. 2024;15:634-654. <https://doi.org/10.3390/microbiolres15020042>

Rashid, M-U, Zaura, E, Buijs, MJ, Keijser, BJF, Crielaard, W, Nord, CE and Weintraub, A. Determining the long-term effect of antibiotic administration on the human normal intestinal microbiota using culture and pyrosequencing methods. *Clin. Infect. Dis.* 2015;60:S77-S84. <https://doi.org/10.1093/cid/civ137>

Rey, M, Enjalbert, F, Combes, S, Cauquil, L, Bouchez, O and Monteil, V. Establishment of ruminal bacterial community in dairy calves from birth to weaning is sequential. *J. Appl. Microbiol*. 2013;116:245-257. <https://doi.org/10.1111/jam.12405>

Reyes, DC, Meredith, J, Puro, L, Berry, K, Kersbergen, R, Soder, KJ, et al. Maine organic dairy producers’ receptiveness to seaweed supplementation and effect of *Chondrus crispus* on enteric methane emissions in lactating cows. *Front. Vet. Sci.* 2023;10:1153097. <https://doi.org/10.3389/fvets.2023.1153097>

Rezaeian, M, Beakes, GW and Parker, DS. Distribution and estimation of anaerobic zoosporic fungi along the digestive tracts of sheep. *Mycol. Res.* 2004;108:10:1227-1233. doi:10.1017/S0953756204000929

Roehe, R, Dewhurst, RJ, Duthie, CA, Rooke, JA, McKain, N, Ross, DW, et al. Bovine host genetic variation influences rumen microbial methane production with best selection criterion for low methane emitting and efficiently feed converting hosts based on metagenomic gene abundance. *PLoS Genetics*. 2016;12:e1005846. <https://doi.org/10.1371/journal.pgen.1005846>

Romero-Pérez, A, Okine, EK, Guan, LL, Duval, SM, Kindermann, M and Beauchemin, KA. Effects of 3-nitrooxypropanol and monensin on methane production using a forage-based diet in Rusitec fermenters. *Anim. Feed Sci. Technol*. 2016;220:67-72. <https://doi.org/10.1016/j.anifeedsci.2016.07.013>

Roque, BR, Brooke, CG, Ladau, J, Polley, T, Marsh, LJ, Najafi, N, et al. Effect of the macroalgae *Asparagopsis taxiformis* on methane production and rumen microbiome assemblage. *Ani. Microbiome*. 2019a;1:3. <https://doi.org/10.1186/s42523-019-0004-4>

Roque, BM, Salwen, JK, Kinley, R and Kebreab, E. Inclusion of *Asparagopsis armata* in lactating dairy cows diet reduces enteric methane emission by over 50 percent. *J. Clean. Prod.* 2019b;234:132-138. <https://doi.org/10.1016/j.jclepro.2019.06.193>

Russell, JB. The importance of pH in the regulation of ruminal acetate to propionate ratio and methane production *in vitro*. *J. Dairy Sci.* 1998;81:12:3222-3230. <https://doi.org/10.3168/jds.S0022-0302(98)75886-2>

Russell, JB and Houlihan, AJ. Ionophore resistance of ruminal bacteria and its potential impact on human health. *FEMS Microbiol*. *Rev*. 2003;27:65-74. <https://doi.org/10.1016/S0168-6445(03)00019-6>

Russell, JB and Rychlik, JL. Factors that alter rumen microbial ecology. *Science*. 2001;292:5519:1119-1122. <https://doi.org/10.1126/science.1058830>

Sasson, G, Ben-Shabat, SK, Seroussi, E, Doron-Faigenboim, A, Shterzer, N, Yaacoby, S, et al. Heritable bovine rumen bacteria are phylogenetically related and correlated with the cow’s capacity to harvest energy from its feed. *mBio*. 2017;8:e00703-17. <https://doi.org/10.1128/mbio.00703-17>

Schilde, M, von Soosten, D, Hüther, L, Meyer, U, Zeyner, A and Dänicke, S. Effects of 3-nitrooxypropanol and varying concentrate feed proportions in the ration on methane emission, rumen fermentation and performance of periparturient dairy cows. *Arch. Anim. Nutri.* 2021;75:2:79-104. <https://doi.org/10.1080/1745039X.2021.1877986>

Seo, JK, Kim, S-W, Kim, MH, Upadhaya, SD, Kam, DK and Ha, JK. Direct-fed microbials for ruminant animals. *Asian-Aust. J. Anim. Sci.* 2010;23:12:1657-1667. <https://doi.org/10.5713/ajas.2010.r.08>

Sharifi, M, Taghizadeh, A, Hosseinkham, A, Palangi, V, Macit, M, Salem, AZM, Elghndour, MMMY and Abachi, S. Influence of nitrate supplementation on *in vitro* methane emission, milk production, ruminal fermentation, and microbial methanotrophs in dairy cows fed two forage levels. *Ann. Ani. Sci.* 2022;22:3:1015-1026. <https://doi.org/10.2478/aoas-2021-0087>

Shen, J, Liu, Z, Yu, Z and Zhu, W. Monensin and Nisin affect rumen fermentation and microbiota differently *in vitro*. *Front. Microbiol*. 2017;8:1111. <https://doi.org/10.3389/fmicb.2017.01111>

Shi, W, Moon, CD, Leahy, SC, Kang, D, Froula, J, Kittelmann, S, et al. Methane yield phenotypes linked to differential gene expression in the sheep rumen microbiome. *Genome Res.* 2014;24:9:1517-1525. <https://doi.org/10.1101/gr.168245.113>

Singh, BK, Chopra, RC, Rai, SN, Verma, MP and Mohanta, RK. Nutritional evaluation of seaweed on nutrient digestibility, nitrogen balance, milk production and composition in Sahiwal cows. *Proc. Natl. Acad. Sci. India*. 2015;87:437-443. <https://doi.org/10.1007/s40011-015-0616-8>

Skillman, LC, Evans, PN, Naylor, GE, Morvan, B, Jarvis, GN and Joblin, KN. 16S ribosomal DNA-directed PCR primers for ruminal methanogens and identification of methanogens colonizing young lambs. *Anaerobe*. 2004;10:277-285. <https://doi.org/10.1016/j.anaerobe.2004.05.003>

Smith, TJ and Murrell, JC. Methanotrophy/methane oxidation. *Encycl. Microbiol*. 2009;3:293-298. <https://doi.org/10.1016/B978-012373944-5.00054-7>

Snelling, TJ and Wallace, RJ. The rumen microbial metaproteome as revealed by SDS-PAGE. *BMC Microbiol*. 2017;17:9. <https://doi.org/10.1186/s12866-016-0917-y>

Snelling, TJ, Auffret, MD, Duthie, C-A, Stewart, RD, Watson, M, Dewhurst, RJ, et al. Temporal stability of the rumen microbiota in beef cattle, and response to diet and supplements. *Anim. Microbiome*. 2019;1:16. <https://doi.org/10.1186/s42523-019-0018-y>

Solomon, KV, Haitjema, CH, Henske, JK, Gilmore, SP, Borges-Riveria, D, Lipzen, A, et al. Early-branching gut fungi possess a large, comprehensive array of biomass-degrading enzymes. *Science*. 2016;351:6278. <https://doi.org/10.101126/science.aad1431>

Soren, NM, Malik, PK and Sejian, V. Chapter 23: Methanotrophs in Enteric Methane Mitigation. In: Livestock production and Climate Change, No. 6. Eds. P.K. Malik *et al*. 2015:360-375. <https://doi.org/10.1079/9781780644325.0360>

Spor A, Koren O and Ley R. Unravelling the effects of the environment and host genotype on the gut microbiome. *Nat. Rev. Microbiol.* 2011;9:279-290. <https://doi.org/10.1038/nrmicro2540>

Stefenoni, HA, Räisänen, SE, Cueva, SF, Wasson, DE, Lage, CFA, Melgar, A, et al. Effects of the macroalga *Asparagopsis taxiformis* and oregona leaves on methane emission, rumen fermentation, and lactational performance of dairy cows. *J. Dairy Sci*. 2021;10:4:4157-4173. <https://doi.org/10.3168/jds.2020-19686>

Stewart, CS, Flint, HJ and Bryant, MP. The rumen bacteria. In: Hobson PN, Stewart CS. Eds. The rumen microbial ecosystem. London: Chaman & Hall. 1997;pp 10-72

Storm, E and Ørskov, E. The nutritive value of rumen microorganisms in ruminants: 1. Large-scale isolation and chemical composition of rumen microorganisms. *Br. J. Nutri.* 1983;50:2:463-470. <https://doi.org/10.1079/BJN19830114>

Subharat, S, Shu, D, Zheng, T, Buddle, BM, Janssen, PH, Luo, D and Wedlock, DN. Vaccination of cattle with a methanogen protein produces specific antibodies in the saliva which are stable in the rumen. *Vet. Immunol. Immunopathol*. 2015;164:201-207. <https://doi.org/10.1016/j.vetimm.2015.02.008>

Tapio, I, Snelling, TJ, Strozzi, F and Wallace, RJ. The ruminal microbiome associated with methane emissions from ruminant livestock. *J. Anim. Sci. Biotechnol*. 2017;8:7. <https://doi.org/10.1186/s40104-017-0141-0>

Tajima, K, Nonaka, I, Higuchi, K, Takusari, N, Kurihara, M, Takenaka, A, et al. Influence of high temperature and humidity on rumen bacterial diversity in Holstein heifers. *Anaerobe*. 2007;13:57-64. <https://doi.org/10.1016/j.anaerobe.2006.12.001>

Tavendale, MH, Meagher, IP, Pacheco, D, Walker, N, Atwood, GT and Subathira, S. Methane production from *in vitro* rumen incubations with *Lotus pedunculatus* and *Medicago sativa* and effects of extractable condensed tannin fractions on methanogenesis. *Anim. Feed Sci. Technol*. 2005;123:124:403-419. <https://doi.org/10.1016/j.anifeedsci.2005.04.037>

Taxis, TM, Wolff, S, Gregg, SJ, Minton, NO, Zhang, C, Dai, J, et al. The players may change but the game remains: network analyses of ruminal microbiomes suggest taxonomic differences mask functional similarity. *Nucleic Acids Res.* 2015;43:20:9600-9612. <https://doi.org/10.1093/nar/gkv973>

Thiele, JH, Chartrain, M and Zeikus, JG. Control of interspecies electron flow during anaerobic-digestion – role of floc fermentation in syntrophic methanogenesis. *Appl. Environ. Microbiol*. 1988;54:10-19. <https://doi.org/10.1128/aem.54.1.10-19.1988>

Thompson, LR, Beck, MR, Gunter, SA, Williams, GD, Place, SE and Reuter, RR. An energy supplement with monensin reduces methane emission intensity of stocker cattle grazing winter wheat. *Appl. Anim. Sci.* 2019;35:433-440. <https://doi.org/10.15232/aas.2018-01841>

Troy, SM, Duthie, C-A, Hyslop, JJ, Roehe, R, Ross, DW, Wallace, RJ, et al. Effectiveness of nitrate addition and increased oil content as methane mitigation strategies for beef cattle fed two contrasting basal diets. *J. Anim. Sci.* 2015;93:1815-1823. <https://doi.org/10.2527/jas2014-8688>

Turnbaugh, PJ, Backhed, F, Fulton, L and Gordon, JI. Diet-induced obesity is linked to marked but not reversible alterations in the mouse distal gut microbiome. *Cell Host Microbe*. 2008;3:213-223. <https://doi.org/10.1016/j.chom.2008.02.015>

Turnbaugh, PJ, Ridaura, VK, Faith, JJ, Rey, FE, Knight, R and Gordon, JI. The effect of diet on the human gut microbiome: a metagenomic analysis in humanized gnotobiotic mice. *Sci. Transl. Med.* 2009;1:6. <https://doi.org/10.1126/scitranslmed.3000322>

Ungerfeld, EM, Rust, SR and Burnett, R. Use of some novel alternative electron sinks to inhibit ruminal methanogenesis. *Reprod. Nutr. Dev.* 2003;43:189-202. <https://doi.org/10.1051/rnd:2003016>

Ungerfeld, EM, Kohn, RA, Wallace, RJ and Newbold, CJ. A meta-analysis of fumarate effects on methane production in ruminal batch cultures. *J. Anim. Sci.* 2007;85:2556-2563. <https://doi.org/10.2527/jas.2006-674>

Ungerfeld, EM. A theoretical comparison between two ruminal electron sinks. *Front. Microbiol*. 2013;4:319. <https://doi.org/10.3389/fmicb.2013.00319>

Ungerfeld, EM. Shifts in metabolic hydrogen sinks in the methanogenesis-inhibited ruminal fermentation: a meta-analysis. *Front. Microbiol.* 2015;6:37. <https://doi.org/10.3389/fmicb.2015.00037>

Uyeno, Y, Sekiguchi, Y, Tajima, K, Takenaka, A, Kurihara, M and Kamagata, Y. An rRNA-based analysis for evaluating the effect of heat stress on the rumen microbial composition of Holstein heifers. *Anaerobe*. 2010;16:27-33. <https://doi.org/10.1016/j.anaerobe.2009.04.006>

Van Soest, PJ. Nutritional Ecology of the Ruminant. 2^nd^ Ed. Cornell University. 1994. <http://www.jstor.org/stable/10.7591/j.ctv5rf668>

Van Zijderveld, SM, Gerrits, WJJ, Apajalahti, JA, Newbold, JR, Dijkstra, J, Leng, RA and Perdok, HB. Nitrate and sulfate: Effective alternative hydrogen sinks for mitigation of ruminal methane production in sheep. *J. Dairy Sci.* 2010;93:12:5856-5866. <https://doi.org/10.3168/jds.2010-3281>

Volmer, JG, McRae, H and Morrison, M. The evolving role of methanogenic archaea in mammalian microbiomes. *Front. Microbiol*. 2023;14:1268451. <https://doi.org/10.3389/fmicb.2023.1268451>

Wallace, RJ, Rooke, JA, McKain, N, Duthie, C-A, Hyslop, JJ, Ross, DW, et al. The rumen microbial metagenome associated with high methane production in cattle. *BMC Genomics*. 2015;16:839. <https://doi.org/10.1186/s12864-015-2032-0>

Wallace, RJ, Sasson, G, Garnsworthy, PC, Tapio, I, Gregson, E, Bani, P, et al. A heritable subset of the core rumen microbiome dictates dairy cow productivity and emissions. *Sci. Adv.* 2019;5:eaav8391. <https://doi.org/10.1126/sciadv.aav8391>

Wang, B, Ma, MP, Diao, QY and Tu, Y. Saponin-induced shifts in the rumen microbiome and metabolome of young cattle. *Front. Microbiol*. 2019;10:356. <https://doi.org/10.3389/fmicb.2019.00356>

Wang, L, Li, Y, Zhang, Y and Wang, L. The effects of different concentrate-to-forage ratio diets on rumen bacterial microbiota and the structures of Holstein cows during the feeding cycle. *Animals*. 2020;10:957. <https://doi.org/10.3390/ani10060957>

Weimer, PJ. Redundancy, resilience, and host specificity of the ruminal microbiota: implications for engineering improved ruminal fermentations. *Front. Microbiol*. 2015;6:296. <https://doi.org/10.3389/fmicb.2015.00296>

Weimer, PJ, Cox, MS, Vieira de Paula, T, Lin, M, Hall, MB and Suen, G. Transient changes in milk production efficiency and bacterial community composition resulting from near-total exchange of ruminal contents between high- and low-efficiency Holstein cows. *J. Dairy Sci.* 2017;100:7165-7182. <https://doi.org/10.3168/jds.2017-12746>

Whittenbury, R, Phillips KC and Wilkinson, JF. Enrichment, isolation and some properties of methane-utilizing bacteria. *J. Gen. Microbiol*. 1970;61:205-218. <https://doi.org/10.1099/00221287-61-2-205>

Williams, AG and Coleman, GS. The rumen protozoa. In: Hobson, P.N., Stewart, C.S. (eds) The Rumen Microbial Ecosystem. 1997. Springer, Dordrecht. <https://doi.org/10.1007/978-94-009-1453-7_3>

Wolin, MJ. A theoretical rumen fermentation balance. *J. Dairy Sci.* 1960;43:10:1452-1459. <https://doi.org/10.3168/jds.S0022-0302(60)90348-9>

Wright, ADG, Kennedy, P, O’Neil, CJ, Toovey, AF, Popovski, S, Rea, SM, et al. Reducing methane emissions in sheep by immunization against rumen methanogens. *Vaccine*. 2004;22:3976-3985. <https://doi.org/10.1016/j.vaccine.2004.03.053>

Xiang, R, McNally, J, Rowe, S, Jonker, A, Pinares-Patino, CS, Oddy, VH, et al. Gene network analysis identifies rumen epithelial cell proliferation, differentiation and metabolic pathways perturbed by diet and correlated with methane production. *Nat. Sci. Rep.* 2016;6:39022. <https://doi.org/10.1038/srep39022>

Xue, D, Chen, H, and Luo, X. Methane emissions regulated by microbial community response to the addition of monensin and fumarate in different substrates. *Appl. Sci.* 2021;11:6282. <https://doi.org/10.3390/app11146282>

Yáñez-Ruiz, DR, Abecia, L and Newbold, CJ. Manipulating rumen microbiome and fermentation through interventions during early life: a review. *Front. Microbiol*. 2015;6:1133. <https://doi.org/10.3389/fmicb.2015.01133>

Yang, C, McKain, N, McCartney, CA and Wallace, RJ. Consequences of inhibiting methanogenesis on the biohydrogenation of fatty acids in bovine ruminal digest. *Anim. Feed Sci. Technol.* 2019;254:114189. <https://doi.org/10.1016/j.anifeedsci.2019.05.012>

Yu, G, Beauchemin, KA and Dong, R. A review of 3-nitrooxypropanol for enteric methane mitigation from ruminant livestock. *Animals*. 2021;11:3540. <https://doi.org/10.3390/ani11123540>

Yurkovetskiy, L, Burrows, M, Khan, AA, Graham, L, Volchkov, P, Becker, L, et al. Gender bias in autoimmunity is influenced by microbiota. *Immunity*. 2013;39:400-412. <http://dx.doi.org/10.1016/j.immuni.2013.08.013>

Zaneveld, JR, McMinds, R and Thurber, RV. Stress and stability: applying the Anna Karenina principle to animal microbiomes. *Nat. Microbiol.* 2017;2:17121. <https://doi.org/10.1038/nmicrobiol.2017.121>

Zhang, L, Huang, X, Xue, B, Peng, Q, Wang, Z, Yan, T and Wang, L. Immunization against rumen methanogenesis by vaccination with a new recombinant protein. *PLoS ONE*. 2015;10:e0140086. <https://doi.org/10.1371/journal.pone.0140086>

Zhang, XM, Gruninger, RJ, Alemu, AW, Wang, M, Tan, ZL, Kindermann, M and Beauchemin, KA. 3-Nitrooxyproponal supplementation had little effect on fiber degradation and microbial colonization of forage particles when evaluated using the in situ ruminal incubation technique. *J. Dairy Sci*. 2020a;103:10:8986 – 8997. <https://doi.org/10.3168/jds.2019-18077>

Zhang, R, Liu, J, Jiang, L and Mao, S. Effect of high-concentrate diets on microbial composition, function, and the VFAs formation process in the rumen of dairy cows. *Anim. Feed Sci. Technol.* 2020b;269:114619. <https://doi.org/10.1016/j.anifeedsci.2020.114619>

Zhao, S, Min, L, Zheng, N and Wang, J. Effect of heat stress on bacterial composition and metabolism in the rumen of lactating dairy cows. *Animals*. 2019;9:11:925. <https://doi.org/10.3390/ani9110925>

Zhou, YY, Mao, HL, Jiang, F, Wang, JK, Liu, JX and McSweeney, CS. Inhibition of rumen methanogenesis by tea saponins with reference to fermentation pattern and microbial communities in Hu sheep. *Anim. Feed Sci. Technol.* 2011;166-167:93-100. <https://doi.org/10.1016/j.anifeedsci.2011.04.007>

Zilber-Rosenberg, I and Rosenberg, E. Role of microorganisms in the evolution of animals and plants: the hologenome theory of evolution. *FEMS Microbiol. Rev.* 2008;32:723-735. <https://doi.org/10.1111/j.1574-6976.2008.00123.x>

**5.** **Supplementary Figure Captions**

Supplementary Figure 1. Examples of current CH4 mitigation strategies for both adults and early-life animals are currently being investigated. In adult animals, using strategies such as inhibitory compounds (i.e., halogenated compounds from algae, 3-NOP), nutrition formulations, vaccines, secondary plant compounds, essential oils and supplementation for alternative electron sinks have shown promise by directly or indirectly inhibiting methanogens and thereby, methanogenesis. A far more promising and long-term solution is the use of mitigation strategies at early-life and then using current animal breeding programs to select for low methane-emitting animals and/or a specific microbiome composition favorable to low CH4 emissions. Adapted from Mizrahi et al. 2021.

Supplementary Figure 2. The issue of species richness and animal performance. In most host-associated microbial ecosystems, a higher alpha diversity (i.e., richness) has often been linked to more efficient resource utilization and more stable microbial communities. An increase in community stability and resource efficiency primarily results in better host performance. However, in the rumen ecosystem, an increase in microbial richness has been linked to lower animal performance. The contrast between high and low microbial richness in the bovine gut is demonstrated above. In panel A, virus A leads to dysbiosis and a diseased lower gastrointestinal microbiome and poor animal performance while a healthy, more efficient host has greater microbial diversity. In the rumen (panel B), the exact opposite process takes place. One theory posited by Ben Shabat et al. (2016) is that the functional redundancy seen in a highly diverse microbiome produces a larger range of output metabolites from their corresponding food webs. This overabundance of metabolites leads to many performance-hindering pathways (i.e., methanogenesis) becoming necessary to utilize the plethora of resources. On the other hand, the less diverse ecosystem produces a smaller range, yet more relevant group of metabolites used for better resource efficiency and thus, animal performance.
